# Explainable artificial intelligence (xAI) in neuromarketing/consumer neuroscience: an fMRI study on brand perception

**DOI:** 10.3389/fnhum.2024.1305164

**Published:** 2024-03-22

**Authors:** José Paulo Marques dos Santos, José Diogo Marques dos Santos

**Affiliations:** ^1^Department of Business Administration, University of Maia, Maia, Portugal; ^2^Unit of Experimental Biology, Faculty of Medicine, University of Porto, Porto, Portugal; ^3^LIACC – Artificial Intelligence and Computer Science Laboratory, University of Porto, Porto, Portugal; ^4^NECE-UBI, Research Centre for Business Sciences, University of Beira Interior, Covilhã, Portugal; ^5^Faculty of Engineering, University of Porto, Porto, Portugal; ^6^Abel Salazar Biomedical Sciences Institute, University of Porto, Porto, Portugal

**Keywords:** xAI (explainable artificial intelligence), neuromarketing, consumer neuroscience, brand perception, fMRI, machine learning, path-weights, Shapley values

## Abstract

**Introduction:**

The research in consumer neuroscience has identified computational methods, particularly artificial intelligence (AI) and machine learning, as a significant frontier for advancement. Previously, we utilized functional magnetic resonance imaging (fMRI) and artificial neural networks (ANNs) to model brain processes related to brand preferences in a paradigm exempted from motor actions. In the current study, we revisit this data, introducing recent advancements in explainable artificial intelligence (xAI) to gain insights into this domain. By integrating fMRI data analysis, machine learning, and xAI, our study aims to search for functional brain networks that support brand perception and, ultimately, search for brain networks that disentangle between preferred and indifferent brands, focusing on the early processing stages.

**Methods:**

We applied independent component analysis (ICA) to overcome the expected fMRI data’s high dimensionality, which raises hurdles in AI applications. We extracted pertinent features from the returned ICs. An ANN is then trained on this data, followed by pruning and retraining processes. We then apply explanation techniques, based on path-weights and Shapley values, to make the network more transparent, explainable, and interpretable, and to obtain insights into the underlying brain processes.

**Results:**

The fully connected ANN model obtained an accuracy of 54.6%, which dropped to 50.4% after pruning. However, the retraining process allowed it to surpass the fully connected network, achieving an accuracy of 55.9%. The path-weights and Shapley-based analysis concludes that, regarding brand perception, the expected initial participation of the primary visual system is followed. Other brain areas participate in early processing and discriminate between preferred and indifferent brands, such as the cuneal and the lateral occipital cortices.

**Discussion:**

The most important finding is that a split between processing brands|preferred from brands|indifferent may occur during early processing stages, still in the visual system. However, we found no evidence of a “decision pipeline” that would yield if a brand is preferred or indifferent. The results suggest the existence of a “tagging”-like process in parallel flows in the extrastriate. Network training dynamics aggregate specific processes within the hidden nodes by analyzing the model’s hidden layer. This yielded that some nodes contribute to both global brand appraisal and specific brand category classification, shedding light on the neural substrates of decision-making in response to brand stimuli.

## 1 Introduction

Foretold as one out of three “fronts” of consumer neuroscience advances, computational methods, more specifically, artificial intelligence, and, even more specifically, machine learning methods may be applied to build mathematical models relating to brain and consumer behaviors (Smidts et al., 2014). Nonetheless, the first attempts were taking place at that time, using functional magnetic resonance imaging (fMRI) and artificial neural networks (ANNs) to model brand preference (Santos and Moutinho, 2011; Marques dos Santos et al., 2014). In the present article, the authors revisit one decade-old data to re-analyze it in light of recent advancements in explainable artificial intelligence (xAI), aiming to extract knowledge from data and model brain processes related to brand preference.

ANNs have the ability to extract information from data, yielding models with high predicting accuracy if adequately trained and generalizable if the input data are sufficiently representative (Haykin, 2009). Pereira et al. (2009) went through several machine learning methods for fMRI classification. However, they raised some concerns about using ANNs for such purpose, namely that (1) it was unclear that a classification performance increase compensates for the classifiers’ increased complexity, and (2) the black-box nature of the yielded models, which precludes their explanation and interpretation. Regarding the former concern, the authors raised the point of the imbalance between training instances and the relationship among features, which limits complex models, such as the dominating approach of deep learning networks (DNNs). Regarding the latter concern, nonetheless, one may take the example of Hanson et al. (2004), who analyzed the hidden layer nodes to interpret how the classifier decides among seven different categories (face, cat, house, chair, scissor, shoe, and bottle), making clear, for example, the distinction between animate and inanimate. Du et al. (2018) surveyed extensively the application of machine learning classifiers for fMRI-based functional connectivity in brain disorders. They explored the types of classifiers more often employed, model-driven and data-driven methods, and feature selection, signaling some common challenges. One advantage they stated is that machine learning classifiers help find biomarkers, a strategy also adopted in the present study. Hence, although some cautions should be adopted, ANNs are suited for fMRI data analysis and classification.

Pereira et al. (2009) also highlighted the importance of the non-linear nature of ANNs, which deserves consideration. Decision-making in the human brain should be assumed as a non-linear process (Grabenhorst and Rolls, 2011). For example, intertemporal choice is markedly non-linear (Kable, 2014). Ten monetary units between today and tomorrow are not perceived to be the same as between 1 month from today and the next day. Therefore, if one intends to model decisions in the brain, a non-linear method, such as ANNs, should intrinsically better approximate the target problem.

Among the diverse architectures of ANNs, the option here is for a feedforward backpropagation shallow neural network (SNN). It is easy to train because it encompasses a frugal number of hyperparameters and connection weights. Moreover, its simple architecture favors its explainability and interpretability. The strategy is to resort to explainable artificial intelligence (xAI) methods (Montavon et al., 2018; Holzinger et al., 2022) to extract knowledge and understand the calculations of the “black box model” (Adadi and Berrada, 2018; Samek and Müller, 2019; Tjoa and Guan, 2021). Even though the SNN is frugal, it would be difficult to understand for the human mind with its 1,290 connections (as in the present case). XAI is a recent field aiming to solve four difficulties with dense ANNs, namely “opaque systems that offer no insight into its algorithmic mechanisms; interpretable systems where users can mathematically analyze its algorithmic mechanisms; and comprehensible systems that emit symbols enabling user-driven explanations of how a conclusion is reached. (…) truly explainable systems, where automated reasoning is central to output crafted explanations without requiring human post processing as final step of the generative process” (Doran et al., 2018). Hence, the present study uses xAI methods to explain and interpret the ANN, aiming to extract a comprehensible model of brand perception (Roscher et al., 2020).

Chen et al. (2015) published a paradigmatic study combining fMRI and MVPA-based (multi-voxel pattern analysis) data analysis (Mumford et al., 2012) to study brand associations in the consumers’ brain under the brand personality framework (Aaker, 1997). Other recent studies have been combining machine learning data analysis methods with neuroscientific or biometric acquisition methods, such as facial coding (Filipović et al., 2020), electroencephalography (EEG) for preference detection (Aldayel et al., 2021), in this case, comparing distinct methods for feature extraction and classification, labeling with “buy” and “not buy” and testing with an ensemble classifier over EEG data (Georgiadis et al., 2022), or using support vector machine (SVM) over t-statistics fMRI images to study the more effective way to present apparel goods in an online shop (Jai et al., 2021). The approach in the present study is to re-analyze fMRI data searching for functional brain neural networks—because brain functions emerge from networks (Yuste, 2015)—that support brand perception and, ultimately, search for brain networks that disentangle between preferred and indifferent brands. ANNs are used to construct a testable method, which accuracy is accessed in out-of-sample data. Finally, xAI procedures are applied to improve the model’s performance and make the model transparent, explainable, and interpretable for knowledge extraction. In sum, the present study aims to contribute answers to the “what” question raised by Chen et al. (2015): “what is the set of associations that goes through consumers’ minds when they are presented with a particular brand?”

## 2 Method

**Figure 1:**
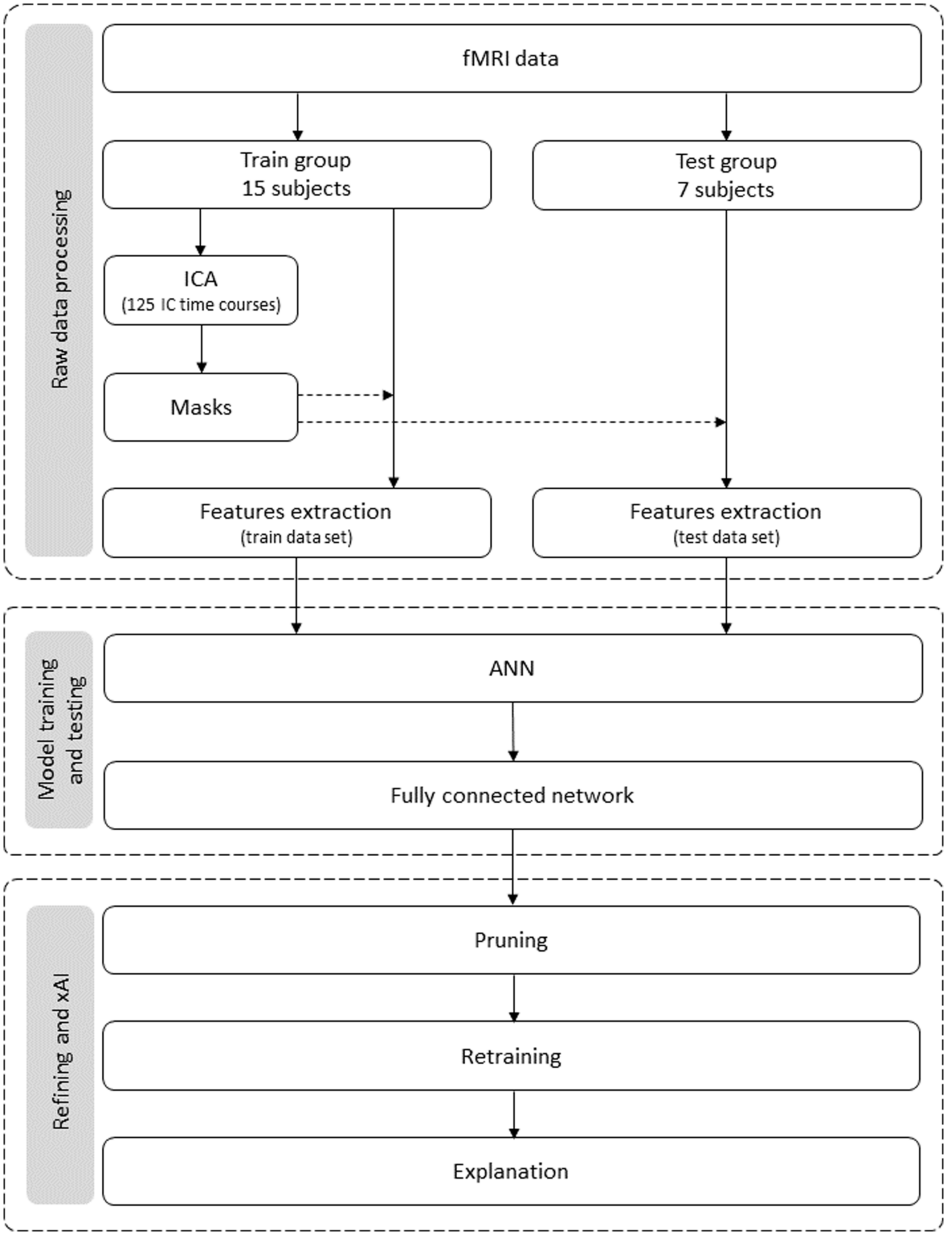
Flowchart of the data analysis process. It encompasses three stages: data preparation, model building, and model refinement and explanation. Data leakage is prevented from the first stage, i.e., each subject’s data belongs to the training dataset or (exclusive; xor) the test set.

The method comprises two stages: the initial fMRI data acquisition and the actual data analysis. Although the initial data acquisition is already described elsewhere (Marques dos Santos et al., 2014), it is again replicated with increased detail. The data analysis method is summarized in Figure 1 and encompasses three stages: (1) feature selection, where the raw data are processed; (2) the construction of the model, which involves its training and testing; and (3) the model refinement and application of xAI procedures for the explanation and interpretation of the model.

### 2.1 fMRI data

Data acquisition is comprised of two sessions: the first is behavioral for stimuli selection and the second is to collect the fMRI data.

#### 2.1.1 First session: stimuli selection

Subjects viewed and rated on a computer screen 200 brands’ logos, one at a time. A five-point version of the self-assessment manikin (SAM) (Morris, 1995; Bradley and Lang, 2007) and the pleasure–arousal–dominance (PAD) scales (Russell and Mehrabian, 1977; Mehrabian and de Wetter, 1987; Mehrabian, 1995) were used for rating purposes. Dominance was discarded because it correlates with the pleasure dimension for still images (Bradley and Lang, 2007). The five-point rates in the pleasure dimension were − 2, −1, 0, +1, and + 2, and for the arousal dimension were 1, 2, 3, 4, and 5. Therefore, each brand logo received a pair of ratings, one for pleasure and another for the arousal dimension.

The 200 brands’ logos were screened according to the criteria:

- Preferred brand: (pleasure +1 or + 2) and (arousal ≥3)
- Indifferent brand: (pleasure 0) and (arousal ≤3)

**Figure 2:**
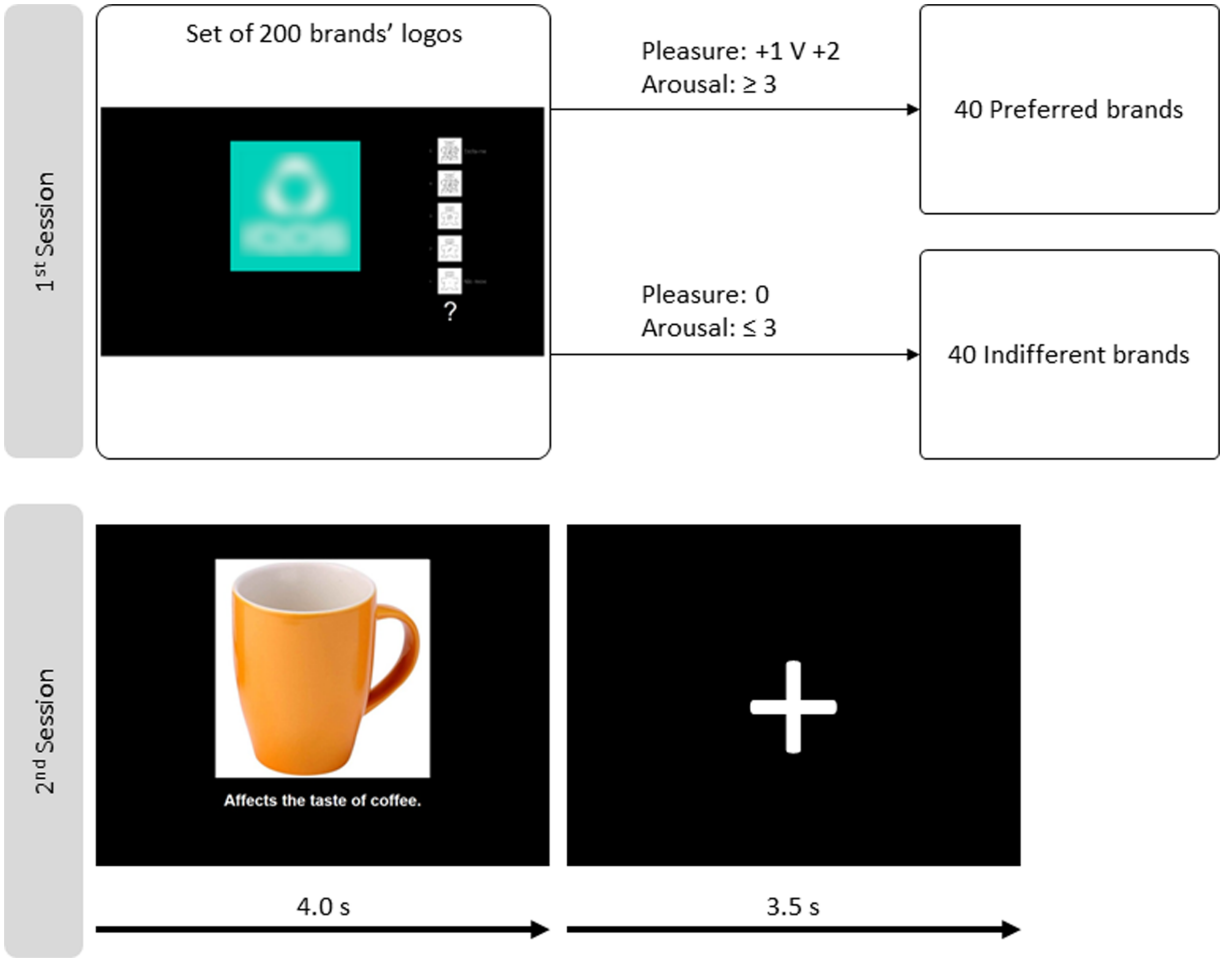
Schemas of the two sessions. The first session, behavioral, on top, is for brand stimuli selection. The self-assessment manikin and the pleasure–arousal–dominance scale are used to rate each brand logo. Two groups of brands are built according to the criteria depicted based on the ratings. The second session schema is in the bottom line, consisting of one exemplary event for fMRI data acquisition. One image depicting a brand logo, a face, or an object is in the centre of the slide. Below, there is a caption that attributes one action to the brand/person/object depicted. This visual stimulation takes 4.0 s and is followed by a fixation cross for 3.5 s.

The logos falling outside the two groups are discarded. Forty logos of each kind were randomly selected per subject for the fMRI session. The top line of Figure 2 portrays the schema.

The first session takes approximately 40 min, including the time needed to complete the brand screening (approximately 10 min) and the verification of including and excluding criteria, sign the informed consent, answer all subjects questions, and pass the instructions.

#### 2.1.2 Paradigm for subjects’ stimulation in the fMRI session

The paradigm used to stimulate subjects was inspired by the one Mitchell et al. (2005) created. In that paradigm, subjects make impressions about people and inanimate objects while their brains are scanned. Subjects view a photograph of one person or an object (a computer or a car). At the same time, the slide includes a statement. The statement describes a situation involving the person or object. For example, “promised not to smoke in his apartment since his roommate was trying to quit” for a person, and “recently had new fog lights installed” for an object (car). Half the statements are positive, and the other half are negative per stimulus category.

In the study by Marques dos Santos et al. (2014), preferred and indifferent brand logos are included, in addition to the images of people and objects. Each image is similarly paired with a statement, half positive and half negative, but these statements start with an action verb. The target in the image is supposedly responsible for that action. For example, “beats his wife (person / negative),” “erases any kind of line” (object: rubber/positive), “uses children labor to make dresses” (brand: garment manufacturer/negative), and “prevents accidents with a sound system” (brand: car manufacturer/positive).

Four categories of stimuli compose the paradigm: brands|preferred (BP), brands|indifferent (BI), objects (O), and people (P). There are 40 examples in each category, 20 paired with a positive statement and 20 with a negative. Each slide is presented for 4.0 s, followed by a fixation cross for 3.5 s. The sequence is depicted in the bottom line of Figure 2. Subjects make impressions of the stimulus passively (without motor participation).

#### 2.1.3 Subjects and fMRI scanning

Twenty-two subjects have complete fMRI acquisitions, 13 male and nine female participants. While they passively made impressions about preferred and indifferent brands, people, and objects, their brains were being scanned. The TR was 2,500 ms, which originated 485 volumes per subject. T1 images were also acquired for co-registration purposes.

The fMRI session takes approximately 1 h. This time includes the reception of the subjects, answering questions, passing the instructions, simulating the answers in the paradigm outside the scanner, the acquisition in the scanner, and finally, debriefing the subjects.

#### 2.1.4 Image pre-processing

The blood-oxygen-level dependent (BOLD) raw files are pre-processed. This stage includes motion correction, slice-timing correction, non-brain removal, spatial smoothing, intensity normalization, and temporal filtering.

The data pre-processing was implemented with FMRI Expert Analysis Tool (FEAT), version 5.98, part of FMRIB’s Software Library (FSL; https://fsl.fmrib.ox.ac.uk/fsl/fslwiki) (Smith et al., 2004; Woolrich et al., 2009; Jenkinson et al., 2012). The following statistics pre-processing was applied: motion correction using MCFLIRT (Jenkinson et al., 2002); slice-timing correction using Fourier-space time-series phase-shifting; non-brain removal using BET (Smith, 2002); spatial smoothing using a Gaussian kernel of FWHM (full width at half maximum) 5 mm; grand-mean intensity normalization of the entire 4D dataset by a single multiplicative factor; and high pass temporal filtering (Gaussian-weighted least-squares straight line fitting, with sigma = 15.0 s). Finally, the “filtered_func_data” data files are registered to the MNI152 space to ensure subjects comparability.

### 2.2 Data processing

The 22 subjects were divided into the training group (15) and the test group (7). This is approximately 2/3 and 1/3, a common split in machine learning.

The voxel count per subject reaches hundreds of thousands. Such a number of inputs would compromise the model training because it is much larger than the instances count per subject, which order is 160 per subject. Such an attempt would lead to an overfitted model. Hence, dimensionality reduction is imposed.

Aiming to reduce the data dimensionality while keeping the information pertinent to the tasks, the data processing consists of two stages: dimensionality reduction with independent component analysis (ICA) and feature extraction. Dimensionality reduction with ICA is, in turn, split into two stages: first, there is the generation of spatial masks, one per IC (independent component), and then the pre-processed data files are screened (with the masks) for time course extraction.

#### 2.2.1 ICA

ICA is a model-free data analysis method extensively applied to fMRI data, for example, used to identify the resting state networks (Beckmann et al., 2005). Although other methods may be suitable for fMRI data dimensionality reduction, ICA was already employed, keeping the information important for model building and proving its effectiveness (Marques dos Santos and Marques dos Santos, 2022, 2023).

The dimensionality reduction process starts with Tensorial Independent Component Analysis (Beckmann and Smith, 2005) as implemented in MELODIC (multivariate exploratory linear decomposition into independent components), version 3.15, part of FSL. The following data pre-processing was applied to the input data: masking of non-brain voxels, voxel-wise de-meaning of the data, and normalization of the voxel-wise variance.

The pre-processed data are whitened and projected into a 125-dimensional subspace using probabilistic principal component analysis (PCA), where the number of dimensions is estimated using the Laplace approximation to the Bayesian evidence of the model order (Minka, 2000; Beckmann and Smith, 2004). The whitened observations are decomposed into sets of vectors that describe signal variation across the temporal domain (time courses), the session/subject domain, and the spatial domain (maps) by optimizing for non-Gaussian spatial source distributions using a fixed-point iteration technique (Hyvärinen, 1999). Estimated component maps are divided by the standard deviation of the residual noise and thresholded by fitting a mixture model to the histogram of intensity values (Beckmann and Smith, 2004). The 125 ICs explain 72.2% of the total variance.

The 125 ICs’ spatial maps screen the voxels in the pre-processed data files. Per subject, the time courses of the surviving voxels are averaged. By the end of the procedure, each IC has its time course containing 485 points. Each IC’s spatial map represents a statistically independent collection of voxels, which are a source of information, sometimes supporting tasks interesting to the study and other times representing study-unrelated tasks.

The spatial images of the main ICs were produced in FSLeyes, version 1.0.13 (McCarthy, 2021). The probabilistic Harvard-Oxford Cortical Structural Atlas deployed in FSLeyes identifies the brain regions. The coordinate system is the MNI 152, corresponding to the “152 non-linear 6th generation” atlas.^1^

#### 2.2.2 Feature extraction

The BOLD signal measured in fMRI scanners varies non-linearly with the neural activity evoked by stimuli and is lagged, peaking between 5 and 7 s after the onset (Martindale et al., 2003; Yeşilyurt et al., 2008). After peaking and the subsequent undershoot, the hemodynamic response returns to a baseline state. Hence, the assumption is that the baseline signal does not carry information pertinent to the interesting tasks, and, on the contrary, the signal acquired near the hemodynamic response peak may contain information pertinent to the task.

The operationalization of this strategy passes by selecting one time point per event. The selected time point is the third after the stimulus onset, corresponding to 6.5 s. Because the event duration (7.5 s) is multiple of the scanner TR (2.5 s), the difference between the selected time point and the onset is fixed.

The procedure is applied for the training and testing datasets. It yields 2,399 instances in the training set (one per event, stimulus category, and subject, i.e., 600 + 600 + 600 + 599, respectively, corresponding to BP, BI, O, and P) and 1,119 instances in the testing set (280 + 280 + 280 + 279, respectively, corresponding to BP, BI, O, and P). Both datasets are standardized. While the output dimension is four (BP, BI, O, and P), the input is 125, i.e., each input node corresponds to one IC.

### 2.3 ANN’s architecture, training, and testing

Deep networks encompass a myriad of hyperparameters that require tuning. However, the number of training instances in the present study is short. Such a complex network would be undertrained or could exhibit overfitting, i.e., it would be trained just for the training dataset, limiting its generalizability. In addition, the purpose of the study is not to achieve high accuracies but to extract pertinent knowledge from the neural network. Intuitively, the more complex the network, the more difficult it will be to extract knowledge from it due to the multiplication of hyperparameters. Conversely, more parsimonious models are always more easily explainable, independently of the xAI techniques that one may have at hand. Thus, the strategy is to start with a network architecture that is as frugal as possible. The exploration of the ANN’s architecture is detailed in section 1 (Exploration of the ANN’s Architecture) of the Supplementary materials. The exploration yields a fully connected shallow neural network (SNN) with a single hidden layer containing 10 hidden nodes.

The network architecture is implemented through the library AMORE, version 0.2–15 (Limas et al., 2014), in R, version 4.3.1 (R Development Core Team, 2010), and RStudio, version 2023.03.0.386 (Posit Team, 2023).

The specific architecture is a backpropagation feedforward artificial neural network. The initial weights and biases are a random field. The training method is the adaptive gradient descent with momentum, and the error criterion is the least mean squares. The activation function in the hidden nodes is “tansig” (hyperbolic tangent). Tansig outputs in the [−1, 1] range with a mean of 0. Because the fMRI data results from a whole brain grand-mean normalization and then is standardized, which turns the baseline values negative, the activation function must account for such cases, and tansig does. In addition, tansig has zero mean, which is important for minimizing the contribution of irrelevant processes. The activation function in the output nodes is “sigmoid,” restraining the outputs to the [0, 1] range. The consideration for a softmax was discarded because it does not yield better information.

The best combination of the hyperparameters’ learning rate and momentum is explored by grid search. The exploration is logarithmic (base 10) for the learning rate from 1e-07 until 0.9. For the momentum, the exploration is linear from 0.05 to 1.00. However, the range between 0.95 and 1.00 is explored in more detail. The combination that maximizes accuracy is 1e-05 for the learning rate and 0.975 for the momentum. In addition, several epoch presentations were explored, and 500 is the number that led to accuracy maximization tested in a different out-of-sample cohort. Finally, 50,000 random fields were generated to test all these parameters and select the network with the highest accuracy. The “best network” accuracy is 54.6%.

### 2.4 Pruning and retraining

After obtaining the model and the best network, the next step is to calculate their path-weights. The $\mathrm{path}\;\mathrm{weigh}t_{ijk}$ is the product of the weights found in the path from input *I_i_* to output *O_k_*, passing by the hidden node *H_j_*:

$$\mathrm{path}\;\mathrm{weigh}t_{ijk}=w_{I_{i}H_{j}}\times w_{H_{j}O_{k}}$$

where $w_{I_{i}H_{j}}$ is the weight between the input node *I_i_* and the hidden node *H_j_*, and $w_{H_{j}O_{k}}$ is the weight between the hidden node *H_j_* and the output node *O_k_*. The analysis of the path-weights aims to identify the connections favored due to magnitudes that are further from zero. Their detailed calculation is explained elsewhere (Marques dos Santos and Marques dos Santos, 2022, 2023).

**Figure 3:**
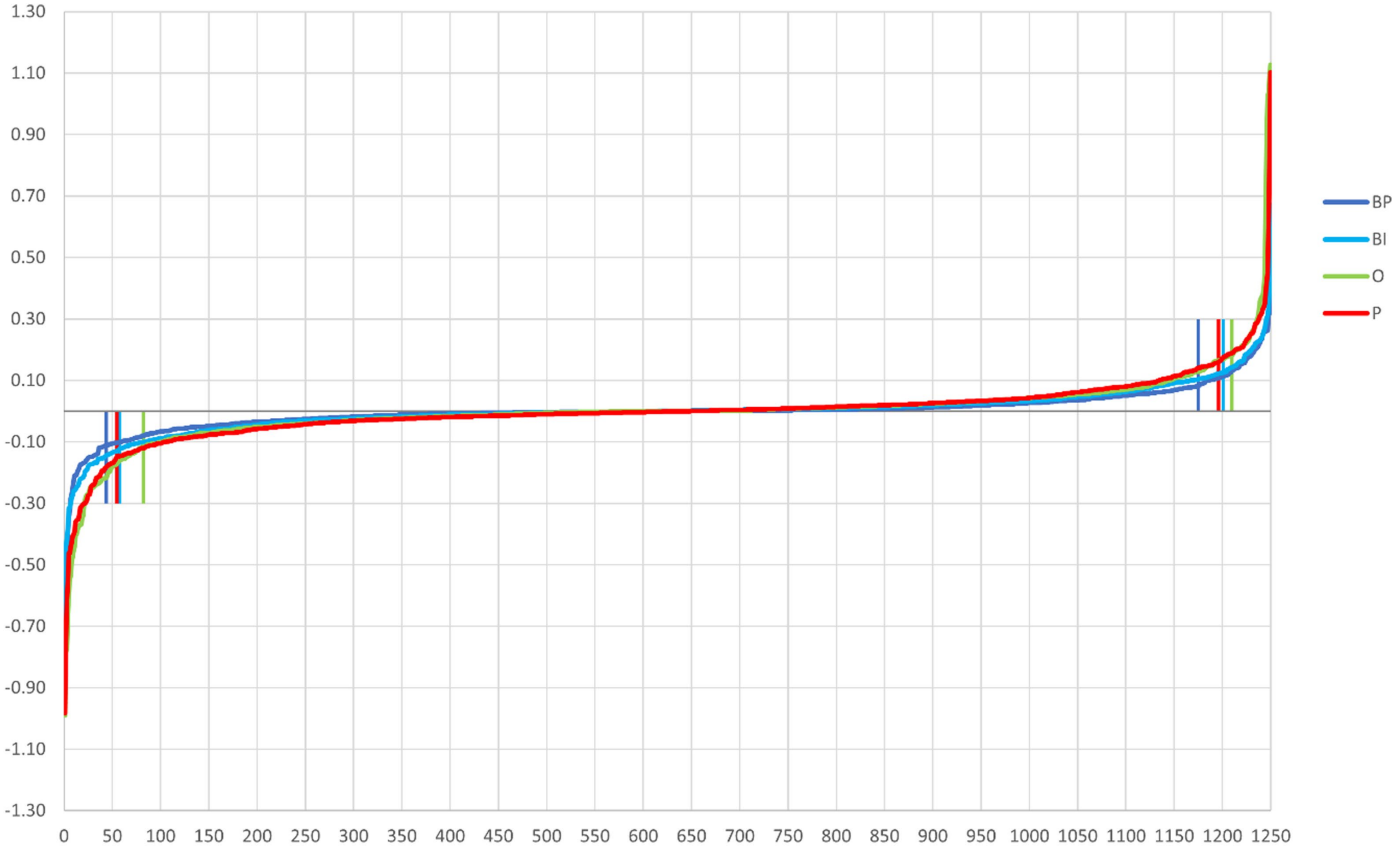
Path-weights plotted per output in increasing order for each stimulus category. All four plots have a tilde shape, meaning that most of the paths have little influence on the computation (because they are close to zero and then tend to cancel the signal in the multiplication), and just a few have weights that move away from nullity. The latter are retained in the pruning process. Vertical lines signal the elbow points, which correspond to BP (dark blue) 44 and 1,175, BI (light blue) 58 and 1,201, O (green) 82 and 1,210, and P (red) 55 and 1,196.

The path-weights are calculated per output. Figure 3 depicts the 1,250 path-weights per output (125 weights input → hidden node × 10 weights hidden node → output node). All the graphs have two elbows, meaning that few paths leverage the information from the input to the output, i.e., weight more in the decision process. The elbow points are calculated with the “pathviewer” R package (Baliga et al., 2023). The elbow points are calculated separately for positive and negative path-weight values. The elbow point is the abscissa of the point in the curve that maximizes the distance to the chord that connects the curve’s two extreme points. The vertical lines in Figure 3 correspond to the calculation of the elbow points for each curve: path-weights 44 and 1,175 for BP, 58 and 1,201 for BI, 82 and 1,210 for O, and 55 and 1,196 for P.

The eight elbow points serve as a threshold for pruning the best network. The complete list of path-weights that survived pruning is reported in Supplementary Tables S1–S4. The respective table cells are color-coded to enhance the path-weights’ importance, either negative or positive.

The pruned network is then retrained. The first step is to explore the best hyperparameter combination by grid search. The learning rate is explored on a logarithmic scale (base 10) ranging from 1e-09 to 1e-04. The exploration is linear for the momentum from 0.700 to 1.000 in 0.025 steps. Each hyperparameter combination is tested 1,000 times (epochs). The combination that maximizes accuracy is 9e-08 for the learning rate and 0.850 for the momentum. The pruned network is retrained with these hyperparameters and the same training dataset for weight tuning. By the end, there is a retrained network with the same architecture as the pruned network (sparsed) but with improved weights now.

### 2.5 Explaining the retrained network

The path-weights analysis is used to explain the retrained network, as shown by Marques dos Santos and Marques dos Santos (2022) and Marques dos Santos and Marques dos Santos (2023). Through the path-weights, this method orders the inputs by their importance, i.e., their role in influencing the final model predictions. In addition, it also helps in interpreting the hidden nodes’ roles in the process.

The calculation of the Shapley values is done based on the retrained network in Python, making use of the SHAP library.^2^ SHAP is SHapley Additive exPlanations. The original network was created in R using the AMORE package, so it must be translated to the PyTorch library.^3^ For this, first, a custom equation that mimics AMORE’s tansig is created, followed by the creation of the model class and model initialization. The weights of the retrained network are then converted to tensors and used to update PyTorch’s model. Then, using the SHAP library, an explainer is created using the DeepExplainer function, an enhanced DeepLIFT algorithm (Shrikumar et al., 2017). As shown by Lundberg and Lee (2017), it can approximate the conditional expectations of Shapley values by integrating over a large number of samples, such that their sum is the difference between the expected and the actual model output. The SHAP values are then calculated for the test set. To complement the standard input analysis using SHAP, the model is then adapted to take the value of the hidden nodes of the original model as input. In this way, it is then possible to obtain the SHAP values not only for the input features but also for the hidden nodes to assess their contribution to the model predictions.

## 3 Results

### 3.1 Networks’ performance

The performances of the three networks are represented in Table 1. It contains the confusion matrix, precisions (local), and accuracies (local and global) of the best network and the pruned and retrained networks. The total number of correct predictions is 611, 564, and 625. Thus, the pruned network shows some loss, which recovers after retraining. The initial global accuracy is 54.6%, which drops to 50.4% after pruning. The retraining recovers the global accuracy to 55.9%. In this case, the retraining leads to outperforming the initial fully connected network.

**Table 1:** Confusion matrices, precisions, and accuracies of the best network and the pruned and retrained networks.

		BP	BI	O	P	Total
Best network	BP	104	82	54	40	280
	BI	94	108	48	30	280
	O	30	43	177	30	280
	P	24	12	21	222	279
	Total	252	245	300	322	1,119
	Precision	41.3%	44.1%	59.0%	68.9%	
	Accuracy	37.1%	38.6%	63.2%	79.6%	54.6%

		BP	BI	O	P	Total
Pruned	BP	116	94	40	30	280
	BI	85	134	44	17	280
	O	66	67	119	28	280
	P	25	39	20	195	279
	Total	292	334	223	270	1,119
	Precision	39.7%	40.1%	53.4%	72.2%	
	Accuracy	41.4%	47.9%	42.5%	69.9%	50.4%

		BP	BI	O	P	Total
Retrained	BP	114	73	53	40	280
	BI	86	111	53	30	280
	O	34	44	171	31	280
	P	18	12	20	229	279
	Total	252	240	297	330	1,119
	Precision	45.2%	46.3%	57.6%	69.4%	
	Accuracy	40.7%	39.6%	61.1%	82.1%	55.9%

Considering the local accuracies, i.e., the number of correct predictions over the number of trials per category, people (P) and objects (O) are tendentially higher than brands|preferred (BP) and brands|indifferent (BI). The only exception is in the pruned network, where BI’s accuracy (47.9%) is higher than O (42.5%). Another remarkable aspect is that pruning improves BP and BI accuracy. After retraining, their accuracies decrease slightly (BP) or more significantly (BI). The pattern exhibited by O and P is similar and more common. Pruning affects both local accuracies, which recover with retraining and are also important. In the case of P, the accuracy surpasses the original.

Local precision is the ratio between correct predictions per category over the total number of predictions the model calculated for that category. The patterns do not change significantly with pruning and retraining. BP, BI, and O decrease with pruning and recover similarly with retraining. P is the opposite because precision increases with pruning and decreases after retraining. However, the magnitudes of the changes are not significant.

### 3.2 Path-weights analysis

The amount of results yielded by the path-weights analysis is enormous, even focusing only on those that survive pruning. The qualitative results presentation that follows aims to emphasize and retain the path-weights, ICs (inputs), and hidden nodes contribute most to the model’s performance measured by accuracy. However, one should consider that the complete model is much broader.

Another aspect that should be considered is that ICA yielded 125 ICs. Some are related to the four categories, the targets of the study, whereas others are unrelated (e.g., related to the paradigm’s secondary aspects, such as the fixation cross visualization, or to physiology, such as breathing and blood circulation). For the sake of space, such results are not reported here, but they justify ICs that highly explain variance (i.e., ICs higher in the rank of importance) and even hidden nodes.

**Figure 4:**
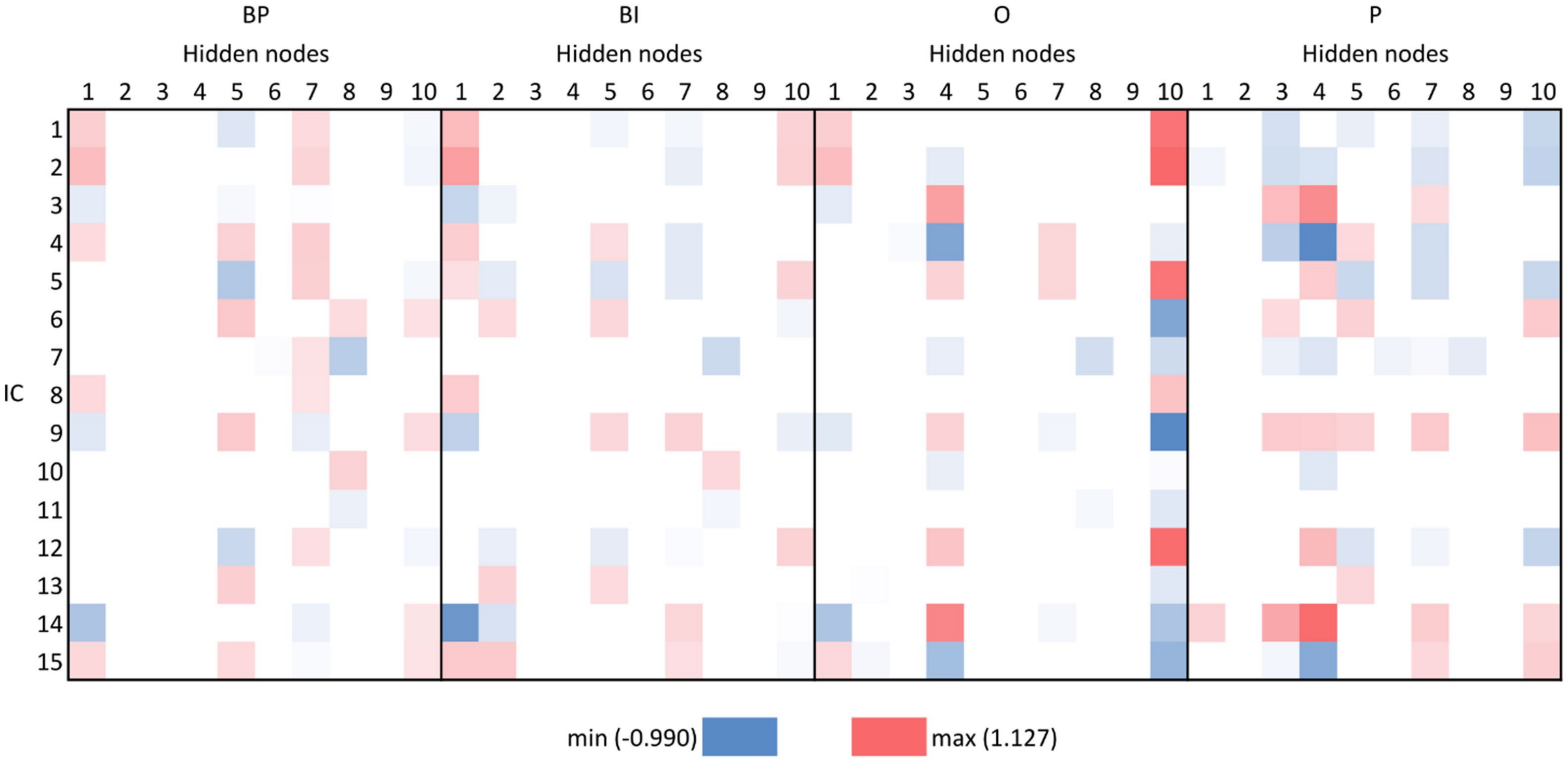
Fifteen first IC’s path-weights per output in the pruned network. White cell means pruned path-weight. The colored cells mean the combination of the input and respective hidden node is retained in the process, either with a positive signal (red) or negative (blue), i.e., that path-weight has a remarkable influence on the output computation either positively or negatively.

Figure 4 depicts the path-weights of the first 15 ICs (inputs) for the four categories: preferred brands (BP), indifferent brands (BI), objects (O), and people (P). The path-weights are color-coded to enhance their importance. The first 15 ICs concentrate on the higher path-weights. The complete lists and values are reported in Supplementary Tables S1–S4. The complete list of the connection weights between the ICs (inputs) and the hidden nodes is reported in Supplementary Table S5, and the complete list containing the weights between the hidden nodes and the outputs is in Supplementary Table S6.

A global appreciation of Figure 4 permits the identification of different patterns for the four categories. In a more detailed way, the hidden nodes 1, 7, and 10 are sufficient to disentangle among the four categories. Hidden node 1 is similar between BP and BI, but hidden nodes 7 and 10 have opposite signals. O’s hidden nodes 1 and 7 are similar to BP (although some ICs are missing in hidden node 7). For P, hidden node 1 is almost non-existent, and hidden node 7 is similar to BP but with the opposite signal. However, hidden node 10 is similar between P and BP and opposite to BI and O.

Other hidden nodes exhibit pertinent particularities. For example, hidden node 2 has an expression for BI only, and hidden node 3 for P. Hidden node 4 has a similar expression for O and P, but it is non-existent for BP and BI. Hidden node 5 is similar for BP, BI, and P but non-existent for O. Hidden nodes 6 and 9 do not have expressions for any of the categories, except for IC7 in P. In any case, its magnitude is low.

Considering the inputs, either because they may contribute with information for three or more categories or because they may highly contribute to a specific category, the ICs that originate path-weights important for the hidden nodes 1, 7, and 10 are IC1, IC2, IC3, IC4, IC5, IC9, IC14, and IC15. Although mostly with negative valence, IC7 participates in important path-weights (mostly passing by hidden node 8) in the four categories and, therefore, should be retained for consideration. IC6 participates in path-weights high for BP and P, and IC12 participates in important path-weights for BP and O.

Even though they are not included in Figure 4, other ICs must be considered due to their importance for specific categories. This is the case of IC40 and IC47, due to their contribution to BP classification, IC78, due to its participation in path-weights important for BI, and IC57 for both categories.

### 3.3 SHAP values

**Figure 5:**
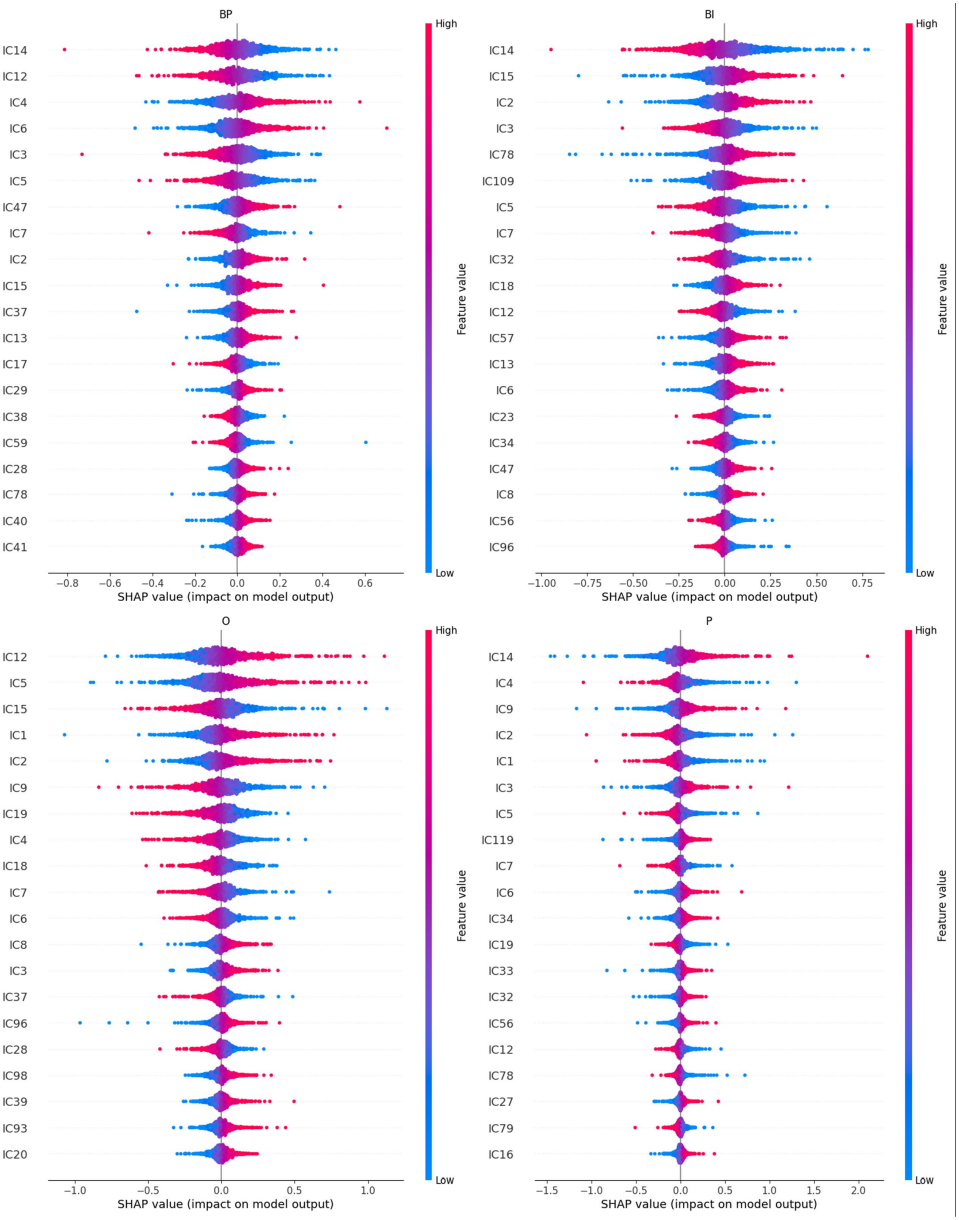
A summary plot containing the SHAP values of the retrained network for the 20th higher-ranked ICs (inputs) for each category (BP, BI, O, and P). Higher rank means more influence in the output computation, either positively (red on the right) or negatively (blue on the right). For example, IC4 and IC6 are the inputs that most positively contribute to the BP output selection, whereas IC15 and IC2 are the inputs that most contribute to BI.

For each category and considering the 20th higher ranked only, the SHAP values of the retrained network are depicted in Figure 5. The full plots of the 125 ICs are reported in the Supplementary materials (Supplementary Figure S2 for BP and BI, and Supplementary Figure S3 for O and P).

Several ICs are in the four categories: IC2, IC3, IC5, IC6, IC7, and IC12. However, a closer look at their polarities reveals that their contribution to the final prediction is not coincidental:

- IC2 is positive in BP, BI, and O and negative in P;
- IC3 is negative in BP and BI and positive in O and P;
- IC5 is negative in BP, BI, and P and positive in O;
- IC6 is positive in BP, BI, and P and negative in O;
- IC 7 is negative in all cases;
- IC12 is negative in BP, BI, and P and positive in O.

With few exceptions, these results overlap with the path-weights analysis (*cf.* Figure 4). Other ICs are in three categories: IC4, IC14, IC15, and IC18. Again, their polarities should be also considered:

- IC4: positive in BP and negative in O and P;
- IC14: negative in BP and BI and positive in P;
- IC15: positive in BP and BI and negative in O;
- IC18: positive in BP and BI and negative in O.

Similarly, with few exceptions, these results reproduce the ones obtained in the path-weights analysis. It should be noted that, despite the different polarities, IC14 is the input that first emerges from Figure 5 because it occupies the first position in three categories: BP, BI, and P. It has negative polarity for the brands.

Other ICs are in two categories and combinations pertinent to the model’s explainability regarding brands. It is the case of IC13 and IC47, which are positive in BP and BI.

**Figure 6:**
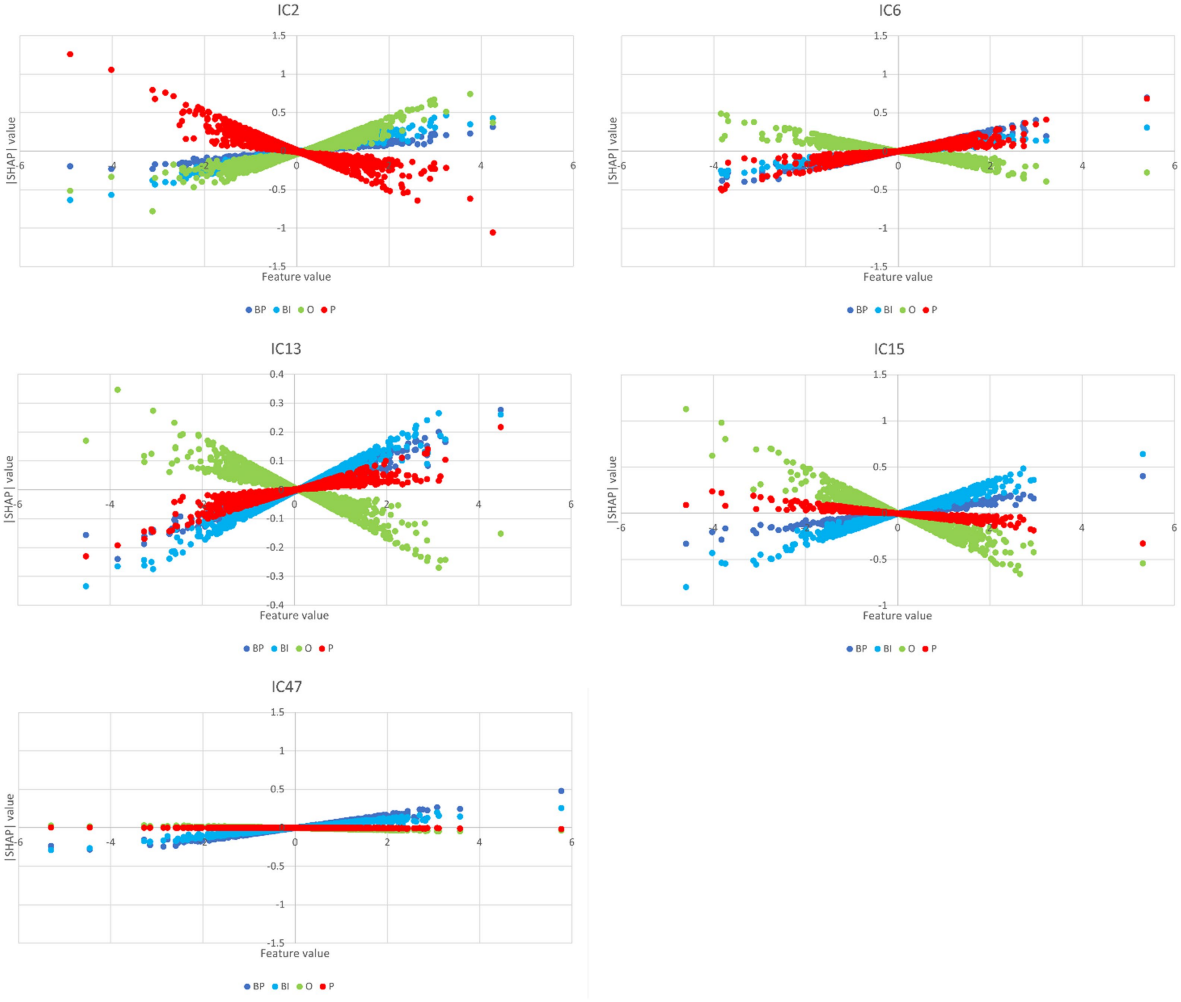
Partial dependence plots of IC2, IC6, IC13, IC15, and IC47. In all these plots, BP and BI have remarkable positive gradients, i.e., these ICs represent brain networks that contain information for correct predictions in brand categories. Thus, these ICs are the most important for BP and BI classification, although IC2, IC6, and IC13 also have a role in O, P, and P classification, respectively.

**Figure 7:**
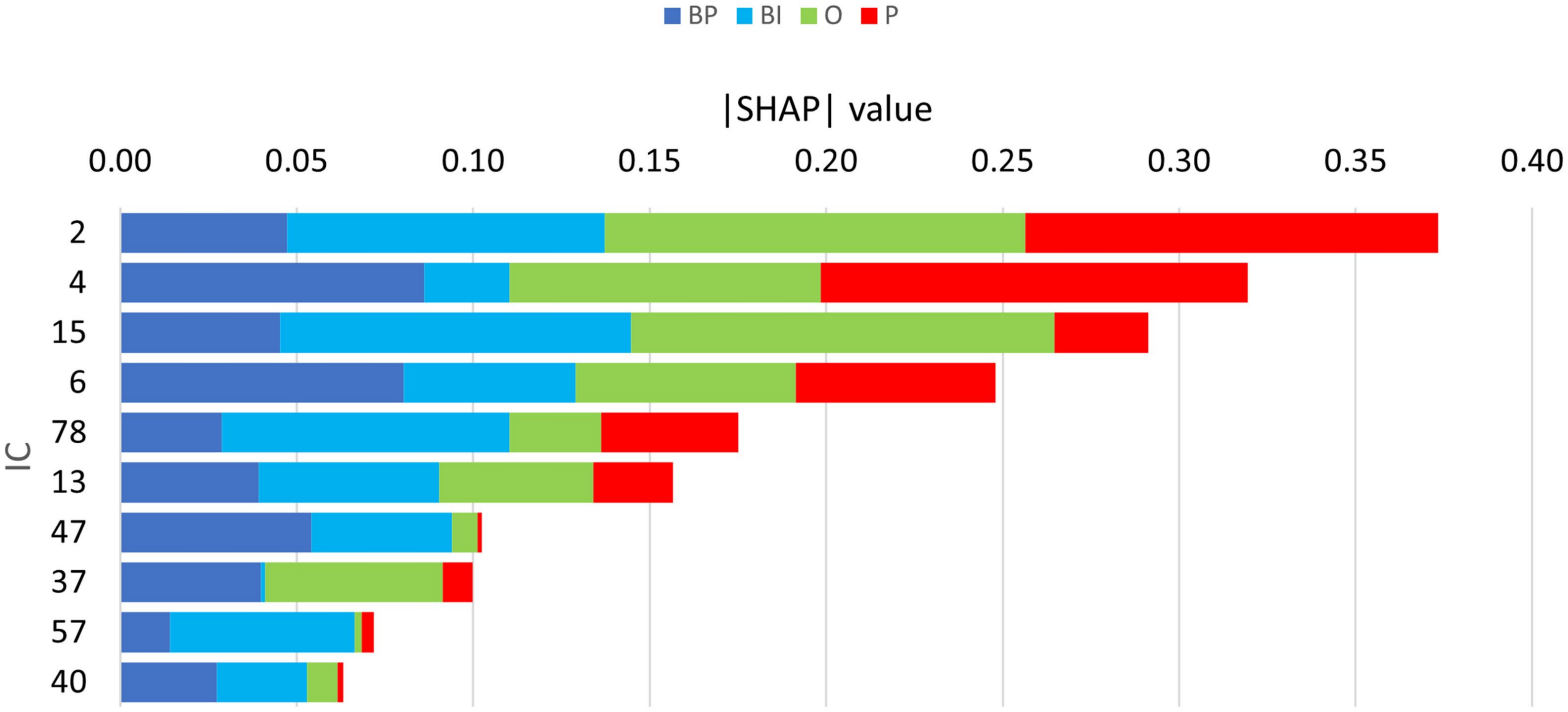
Bar plots of the absolute SHAP values of the retrained network, depicting the 10 ICs considered for focused evaluation. The width of each colored segment is proportional to the SHAP value, i.e., the wider it is, the more important it is for classification, contributing either positively or negatively.

The partial dependence plots in Figure 6 represent the ICs (inputs) that have a higher positive impact on the network decision. Lines with positive gradients represent inputs that contain information pertinent to making correct predictions of the target category. All the five depicted ICs have positive SHAP values impacting BP and BI decisions, i.e., IC2, IC6, IC13, IC15, and IC47. These ICs represent networks containing information that can be correctly classified in BP and BI categories, although some may also contribute to correctly classifying in other categories. Only IC15 and IC47 are positive for BP and BI. Figure 7 depicts the bar plots of these ICs. The bar plots are drawn with absolute SHAP values; therefore, polarity is absent.

**Figure 8:**
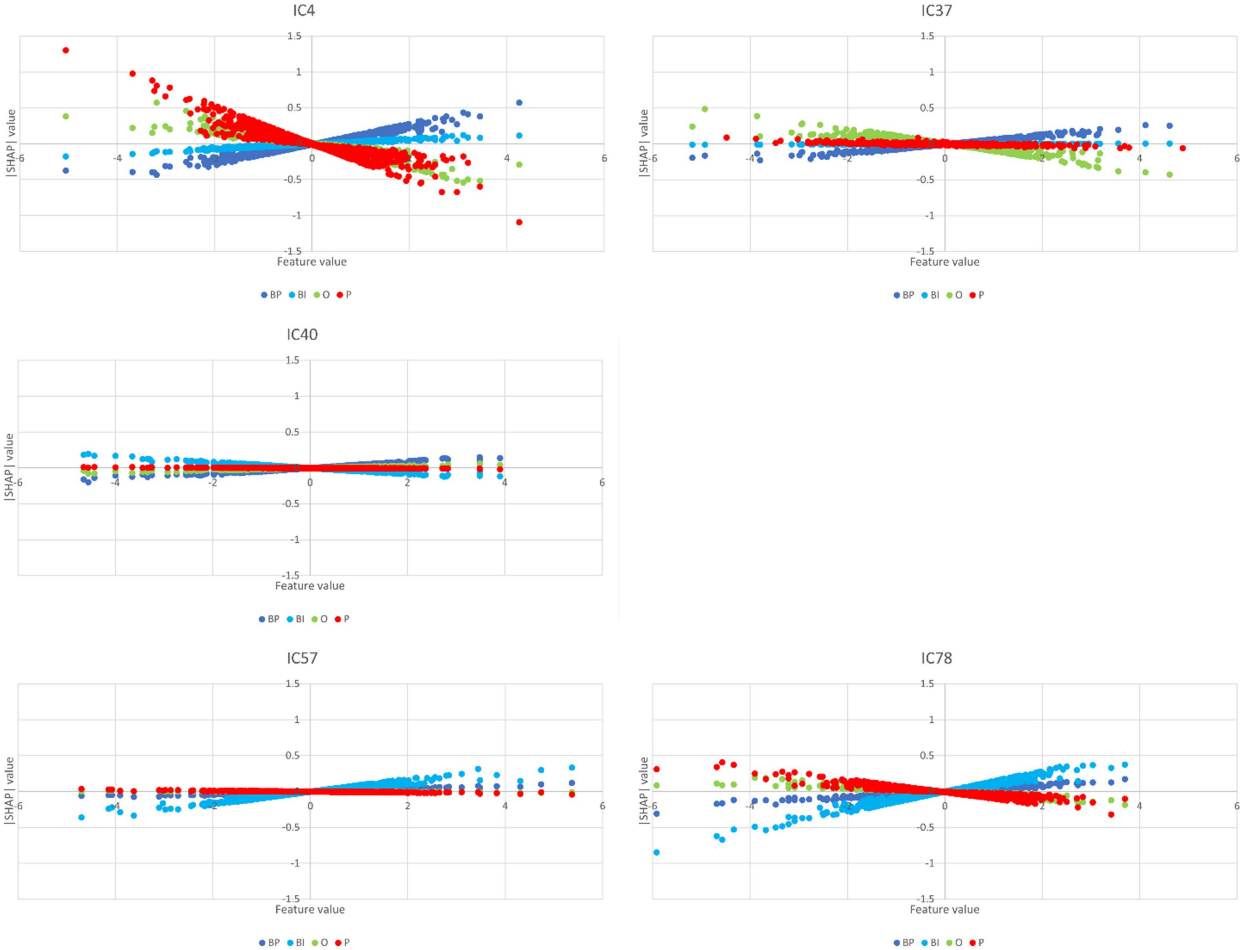
Partial dependence plots of IC4, IC37, IC40, IC57, and IC78. The ICs in the top two rows, IC4, IC37, and IC40, represent brain networks that contain information more pertinent to correct BP predictions (the BP lines formed by dots have positive gradients and are dominant) than the other categories. In the bottom row, the IC57 and IC78 are more important for BI than for BP, O, and P.

Other ICs tend to contribute to a single category, with underrepresentation of the remaining categories or with the other categories exhibiting opposite polarity. That is the case of IC4, IC37, and IC40 for BP and IC57 and IC78 for BI. The partial dependence plots of these ICs are depicted in Figure 8. IC4, IC37, and IC40, in the top two rows, are inputs that represent brain networks containing information pertinent for correct BP predictions (BP lines have positive gradients and are dominant over the three other categories). IC57 and 7IC8, in the bottom row, are important for BI with the same rationale. Figure 7 depicts the bar plots of these five ICs. It is worth noting that this graph is plotted with SHAP absolute values, i.e., polarity is absent.

**Figure 9:**
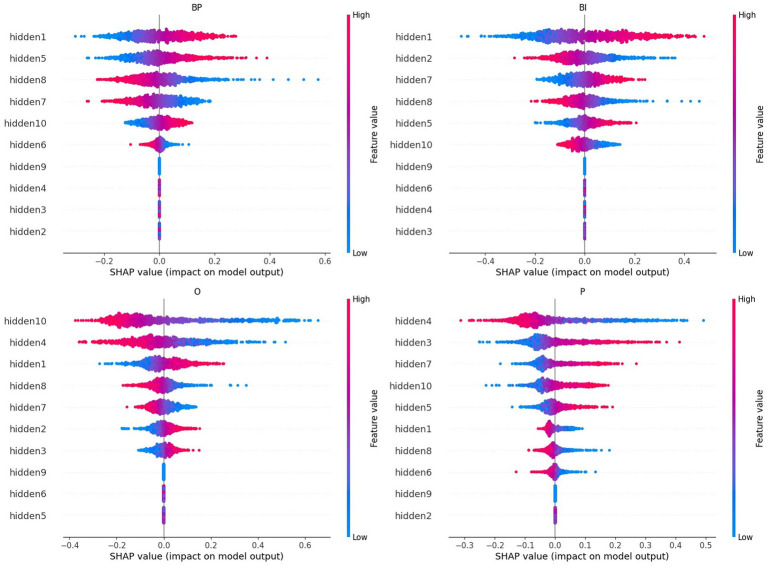
A summary plot containing the absolute SHAP values of the retrained network for the hidden nodes. Higher rank means more influence in the output computation, either positively (red on the right) or negatively (blue on the right). For example, hidden node 1, hidden node 5, and hidden node 10 are the nodes that most positively contribute to the BP output selection, whereas hidden nodes 1, 7, and 5 are the nodes that most contribute to BI.

**Figure 10:**
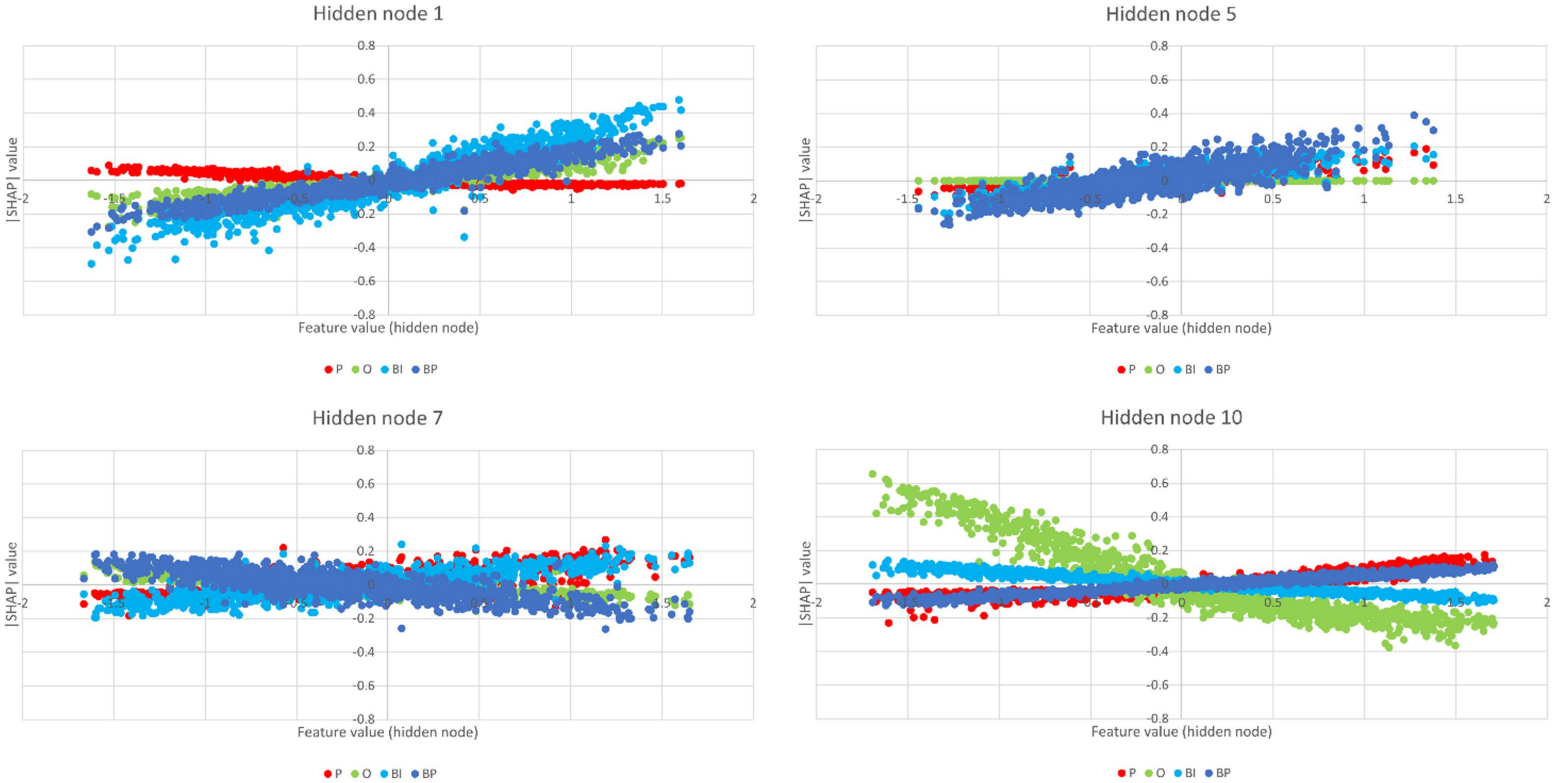
Partial dependence plots of the hidden nodes 1, 5, 7, and 10. Hidden nodes 1, 5, and 10 have positive gradients for BP and, thus, are more important for BP classification, and hidden nodes 1 and 10 have positive gradients for BI, i.e., are important for BI classification.

The SHAP values for the hidden nodes, i.e., the measures that reflect each hidden node’s importance in the final prediction of every category, are depicted in Figure 9. The features, i.e., the hidden nodes, are ranked by the decreasing absolute SHAP value. The respective partial dependence plots that are more important for the two brand categories are depicted in Figure 10.

Considering brands, the hidden nodes 1 and 5 emerge with high-ranked positive SHAP values in both categories, BP and BI. On the negative influence side, hidden node 8 stands out in both categories. Hidden node 7 is high ranked in both cases. However, its polarity is reversed: negative in BP but positive in BI. Hidden node 10 has a lower rank and is reversed: positive in BP and negative in BI. Hidden nodes 2 and 6 have identical polarities, but their rank is inversed: hidden node 2 is higher in BI, and hidden node 6 is higher in BP. Hidden nodes 3, 4, and 9 are irrelevant for classification in both cases (they are null).

Negative valences are in the highest places in O: hidden nodes 10, 4, and 8. Hidden node 1 is O’s highest positive SHAP value, followed by hidden nodes 2 and 3, but with lower magnitudes. Hidden node 4, with negative valence, is the top one for P. The next higher-ranked P’s SHAP values are hidden nodes 3, 7, 10, and 5. The remaining hidden nodes are negative, and hidden nodes 9 and 2 are null (pruned).

Finally, comparing the SHAP values for hidden nodes and the results of the path-weights, one verifies that everything said in the previous section still holds (*cf.* Figure 4). For example, hidden nodes 1, 7, and 10 and their polarities are also sufficient to disentangle the four categories. Hidden node 2 is important for BI, hidden node 3 for P, and hidden node for O and P, but not for the brands.

### 3.4 IC’s spatial maps

**Figure 11:**
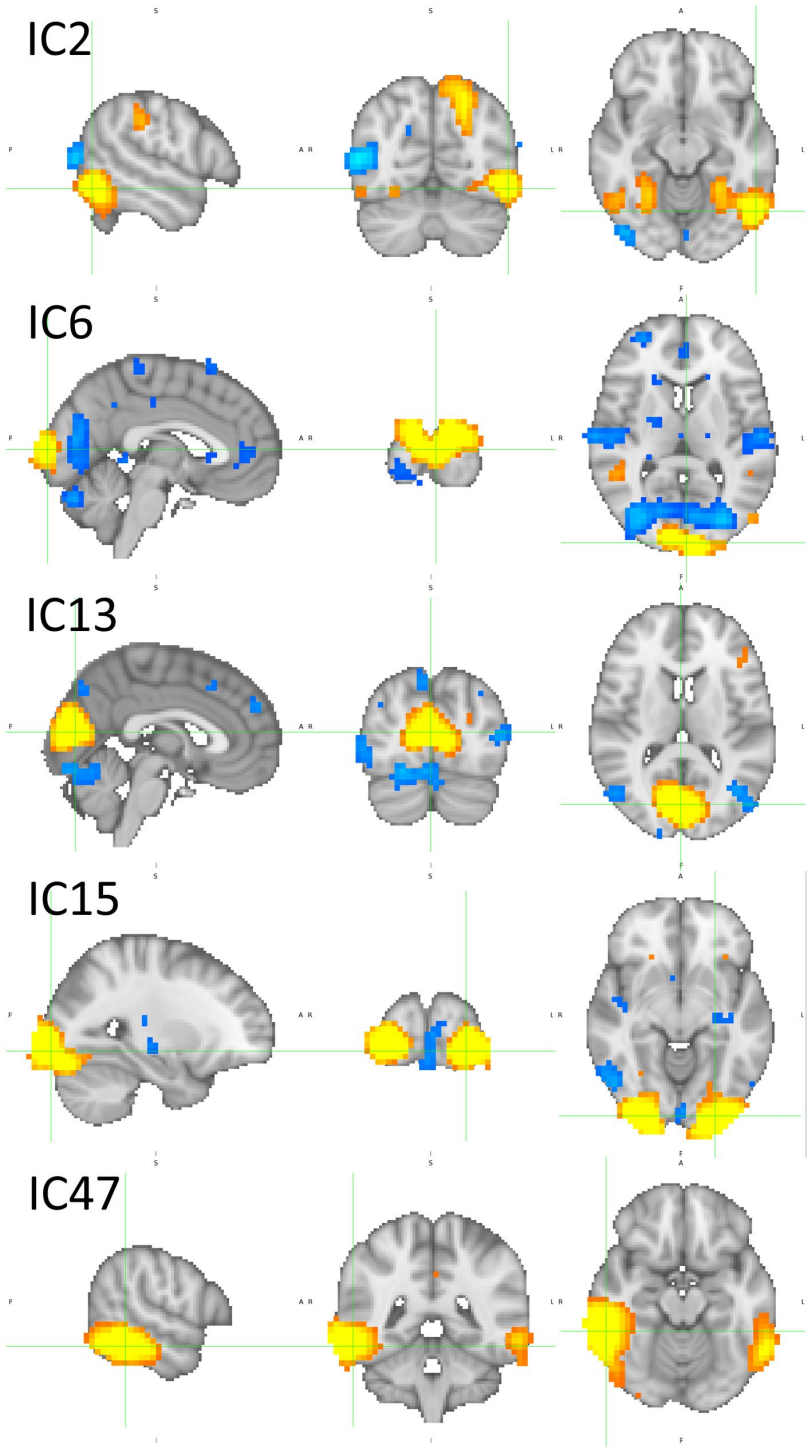
Sagittal, coronal, and axial views of IC2 (x = −54, y = −66, z = −12), IC6 (x = −2, y = −98, z = 8), IC13 (x = 2, y = −78, z = 12), IC15 (x = −26, y = −94, z = −8), and IC47 (x = 58, y = −42, z = −16). These ICs are important in global brand perception. There is a large participation of caudal parts of the brain involved in visual and visual associative processing. Table 2 reports in detail the brain regions involved. Z-values are color-coded in the range − 6.0 (light blue) to −2.6 (dark blue) and 2.6 (red) to 6.0 (yellow). MNI152 standard space. Radiological convention.

Figure 11 depicts five ICs important in brand global perception: IC2, IC6, IC13, IC15, and IC47. Supplementary images of these ICs are provided in Supplementary Figure S6. Both activations and deactivations exist mainly in the caudal parts of the brain, whose function is traditionally connected to visual processing and visual stimuli associations. IC6, however, exhibits a more distributed network, including caudal brain regions and deactivation in the planum temporale and about the superior temporal gyrus. IC13 is characterized by an extensive activation in the caudal-medial brain belonging to the visual cortex and a left-lateralized activation in brain areas involving the temporal lobe’s posterior areas. A more detailed identification of the brain regions activated or deactivated is provided in Table 2.

**Table 2:** Coordinates and brain regions description of the ICs depicted in Figure 11.

IC	x	y	z	Activ/Deact	Regions
IC2	−26	−50	−16	Activation	79% Temporal Occipital Fusiform Cortex
	−54	−66	−12	Activation	64% Lateral Occipital Cortex, inferior division 17% Inferior Temporal Gyrus, temporooccipital part 8% Middle Temporal Gyrus, temporooccipital part
	−22	−74	48	Activation	66% Lateral Occipital Cortex, superior division
	54	−66	8	Deactivation	75% Lateral Occipital Cortex, inferior division
IC6	−2	−98	8	Activation	68% Occipital Pole
	−26	−78	24	Deactivation	59% Lateral Occipital Cortex, superior division
	−58	−18	8	Deactivation	31% Planum Temporale 20% Heschel’s Gyrus 13% Central Opercular Cortex 7% Superior Temporal Gyrus, posterior division
	26	54	24	Deactivation	85% Frontal Pole
IC13	2	−78	12	Activation	56% Supracalcarine Cortex 19% Intracalcarine Cortex 10% Lingual Gyrus 5% Cuneal Cortex
	−50	−66	−8	Activation	69% Lateral Occipital Cortex, inferior division 10% Inferior Temporal Gyrus, temporooccipital part 8% Middle Temporal Gyrus, temporooccipital part
IC15	−26	−94	−8	Activation	49% Occipital Pole 10% Lateral Occipital Cortex, inferior division
	42	−70	8	Deactivation	44% Lateral Occipital Cortex, inferior division 5% Lateral Occipital Cortex, superior division
IC47	58	−42	−16	Activation	34% Inferior Temporal Gyrus, temporooccipital part 10% Middle Temporal Gyrus, temporooccipital part 7% Inferior Temporal Gyrus, posterior division

MNI152 standard space. Brain regions are extracted from the probabilistic Harvard-Oxford Cortical Structural Atlas.

**Figure 12:**
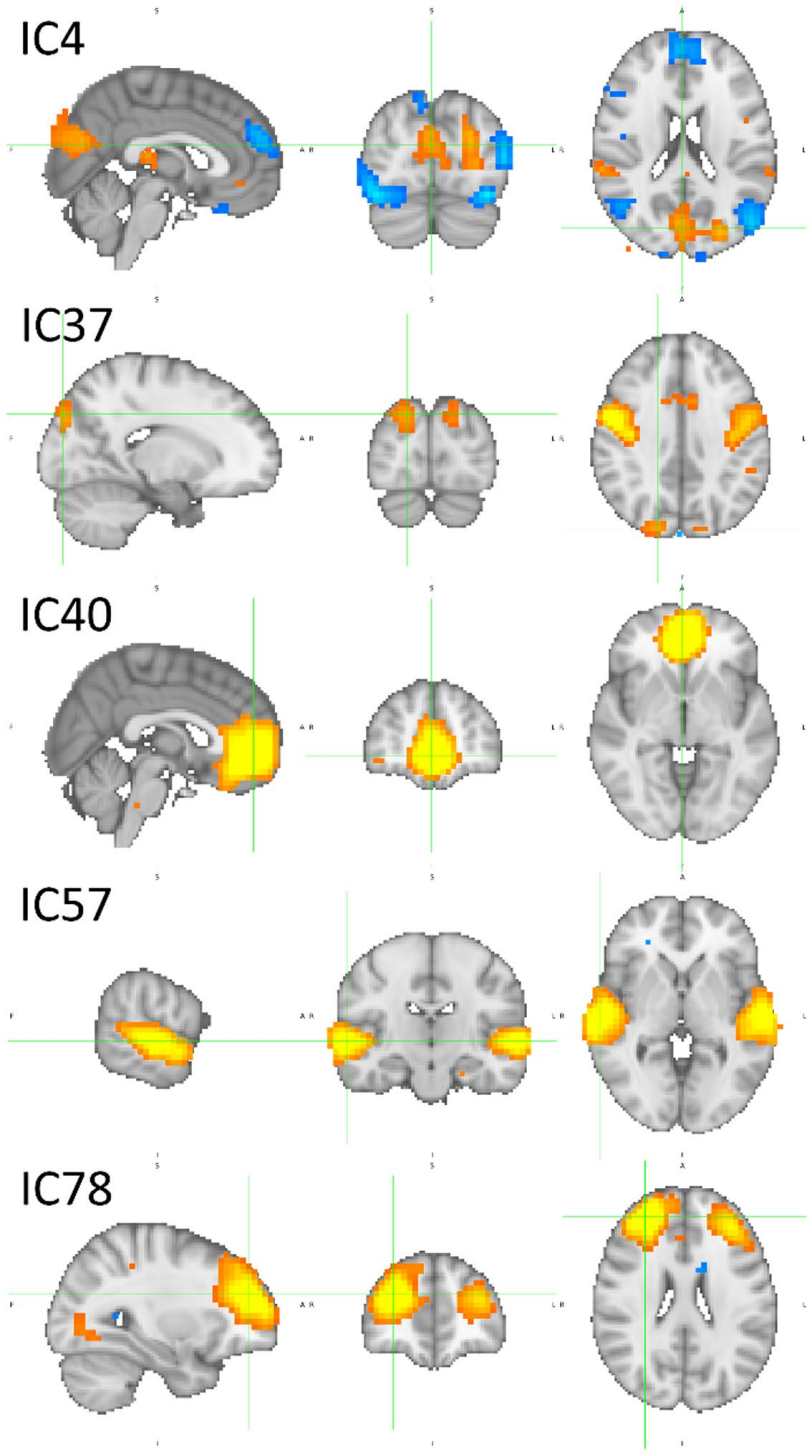
Sagittal, coronal, and axial views of inputs important for BP classification in the top three rows, IC4 (x = 2, y = −78, z = 20), IC37 (x = 18, y = −86, z = 36) and IC40 (x = 2, y = 50, z = −4), and for BI classification in the two bottom rows, IC57 (x = 62, y = −18, z = 0) and IC78 (x = 30, y = 46, z = 24). For BP classification, IC4 involves several parts of the brain, caudal, medial, and rostral, IC37 is mostly medial, and IC40 is rostral. For BI classification, caudal areas are almost absent. The large activations are in the medial and rostral parts of the brain. Table 3 reports in detail the brain regions involved. Z-values are color-coded in the range − 6.0 (light blue) to −2.6 (dark blue) and 2.6 (red) to 6.0 (yellow). MNI152 standard space. Radiological convention.

Figure 12 encompasses statistical parametric maps of ICs that are individually important for brand categories, BP and BI. Supplementary images of these ICs are provided in Supplementary Figures S7, S8. The criterion is to select ICs whose absolute SHAP value is high, which influences the decision, but must have positive gradients solely for that category. The gradients of the remaining three categories should be negative or null (*cf.* Figure 8). In that way, one may assume that the target IC (which represents a brain network) may have an important and unique role when the brain processes the stimulus. The top three rows in Figure 12 refer to BP, i.e., IC4, IC37, and IC40, which represent brain networks mostly devoted to BP classification. The bottom two rows refer to BI. Therefore, IC57 and IC78 mainly participate in BI classification.

Considering the ICs important for BP, IC4 has a complex pattern. It is characterized by a bilateral deactivation in the fusiform, an activation in the cuneal cortex accompanied by two lateral deactivations in the LOC, although the left inferior LOC also has an activation. In addition to the visual and visual associative areas, IC4 also encompasses deactivations in both temporal poles and medial deactivation in the superior frontal gyrus. IC37 also exhibits a complex pattern, containing a deactivation in the central opercular cortex and a prolonged activation occupying bilaterally both precentral gyri, with two maxima: one near the Sylvian fissure and the other much more dorsal. Between the activations in the precentral gyri, there is an activation in the juxtapositional lobule cortex (supplementary motor cortex). Finally, IC 37 also encompasses an activation in the superior LOC, extending to the occipital pole. The IC40 is much simpler. An extensive activation in the ventral medial part of the prefrontal cortex forms it. Table 3 describes in more detail the anatomies of these ICs.

**Table 3:** Coordinates and brain regions description of the ICs depicted in Figure 12.

IC	x	y	z	Activ/Deact	Regions
IC4	2	−78	20	Activation	53% Cuneal Cortex 37% Supracalcarine Cortex
	−50	−74	20	Deactivation	77% Lateral Occipital Cortex, superior division 13% Lateral Occipital Cortex, inferior division
	−46	14	−36	Deactivation	78% Temporal Pole
	−46	−66	−4	Activation	57% Lateral Occipital Cortex, inferior division
	38	−50	−20	Deactivation	88% Temporal Occipital Fusiform Cortex
	6	54	28	Deactivation	58% Superior Frontal Gyrus 16% Frontal Pole
IC37	−54	6	0	Activation	36% Precentral Gyrus 21% Central Opercular Cotex 11% Inferior Frontal Gyrus, pars opercularis 10% Temporal Pole
	−50	−22	16	Deactivation	60% Central Opercular Cortex 25% Parietal Operculum Cortex 6% Heschl’s Gyrus
	18	−86	36	Activation	30% Lateral Occipital Cortex, superior division 25% Occipital Pole
	54	−2	44	Activation	72% Precentral Gyrus
	2	−2	64	Activation	70% Juxtapositional Lobule Cortex (SMC) 5% Precentral Gyrus
IC40	2	50	−4	Activation	68% Paracingulate Gyrus 20% Frontal Medial Cortex
IC57	62	−18	0	Activation	22% Superior Temporal Gyrus, posterior division 9% Planum Temporale
IC78	30	46	24	Activation	79% Frontal Pole

MNI152 standard space. Brain regions are extracted from the probabilistic Harvard-Oxford Cortical Structural Atlas.

Concerning BI, the bilateral superior temporal gyri are the main brain parts encompassed by IC57. These two activations are large, spanning other regions in their vicinity, such as the planum temporale. IC78 pattern is also simple, including two large activations in the rostral zones of the prefrontal cortex. They are lateralized, one on the left and the other on the right hemisphere, occupying areas also known by the dorsolateral prefrontal cortex bilaterally. The detailed information of the two ICs is in Table 3.

## 4 Discussion

Before entering the discussion of the results, three aspects deserve previous consideration: first, all the data analysis procedure is model-free; second, in the fMRI paradigm, subjects were not forced to choose brands on a virtual shelf or similar scenario explicitly; and, third, subjects did not have to record their choices through a button box.

The former aspect is not so common in the fMRI data analysis. Currently, the software packages for fMRI data analysis are based on a general linear model (GLM). GLM, however, requires previous model postulation. The outputs are statistics that, voxel-by-voxel, inform how the data followed the model. Thus, the outputs have a correlational basis. However, the brain functions based on internal networks, and a voxel-by-voxel approach may miss capturing such global dynamics. Multi-voxel pattern analysis (MVPA) and its advantages, focusing on brain decoding, were tackled by Haynes and Rees (2006), and other authors also have been addressing its advantages (Mitchell et al., 2004; Norman et al., 2006; O'Toole et al., 2007; Pereira and Botvinick, 2011; Schwarzkopf and Rees, 2011; Smith, 2013). Du et al. (2018) salient the advantages of data-driven methods as disregarding previous knowledge necessary for model building, running over the whole brain, identifying global networks, detecting overlapping components, and capturing intersubject variability. The approach in the present study is similar, i.e., avoid pre-established models. The model must emerge from data. This strategy aims to avoid biases due to framing with previous conceptions. ICA, a model-free method, is first employed to reduce data dimensionality and extract the functional neural networks. Then, ANNs are model-free also. ANNs build a model from data. Hence, the complete data analysis is model-free, and the resulting model emerges from the data. In addition, the procedure has a global brain approach, capturing its natural dynamics.

Second, the paradigm design prevented prompting unnatural deliberative-based decision-making. Although an fMRI scanner room is not common for consumers, we think this study design could capture brand perception without explicitly calling for it. Consumers were asked to make impressions about the brand, taking into consideration a caption exhibited at the same time in the slide. Thus, subjects did not think if they liked the brand or were indifferent to it, but one may admit that they did that during the process because there are neural differences between BP and BI, even when resorting to the implicit approach employed.

Third and in addition to the previous point, there is no participation of the motor system. Because the motor system signal tends to be dominant in fMRI, the ANN could favor modeling based on it rather than cognitive functions, as in Santos and Moutinho (2011). Hence, the paradigm aimed to capture implicit perceptions of brands and avoid contamination with motor recruitments.

The analysis method also deserves consideration. Reducing the dimensionality with ICA, besides limiting the number of inputs and, mainly, keeping the number of connection weights low enough to guarantee convenient ANN training, also ensures independence among features. Dependent features could lead to weird interpretations of the model and its biological counterparts. Contrarily, in the study by Marques dos Santos et al. (2014), the ICs were screened using a GLM analysis before training the ANN, and in the present study, all the ICs yielded by ICA participated in the ANN training. The screening process is done in the pruning stage, where path-weights of approximately 0 are removed from the model. Avoiding the GLM-based screening, this procedure stresses the model-free nature of the process employed.

### 4.1 ANN’s model and performance

Compared to a similar analysis of the same data (Marques dos Santos et al., 2014), the present study achieved improved accuracy of the fully connected ANN (41.4% in 2014 vs. 54.6% today; *cf.* Table 1). The improvement is more pronounced in O and P than in brands, although upgrading different architectures and procedures may contribute to further developments. For example, pruning and retraining the network pushed accuracy to 55.9%, probably due to sparsity and weight fine-tuning. Nevertheless, other techniques are being developed, especially in the field of explainable artificial intelligence (xAI), which may contribute to further advancements in fMRI-based brain modeling.

In any case, one should consider that (1) the procedure employed here does not rely on previous assumptions about the pertinent constructs, interrelations, and linking processes, i.e., it is model-free, (2) there is not an inference of cognitive processes that could be contaminated by motor actions, and (3) still, the accuracies achieved is well above mere randomness, tested with out-of-sample subjects.

Although the current mainstream calls for deep neural networks, the present study revealed that shallow neural networks (SNNs) are still effective. Because of their frugal parameters, SNNs are specially adapted to analyze current fMRI studies, which tend to involve approximately 30 subjects and a few hundred instances. Otherwise, the risk is to yield undertrained, overfitted models that are poor for knowledge extraction from data.

### 4.2 A neural system for brand perception

The amount of results generated by the two analysis methods is considerable. Therefore, although important results for the process were disclosed in the previous section, the discussion focuses on brand perception, i.e., focus on the categories BP (brand|preferred) and BI (brand|indifferent). There are references to O (objects) and P (people) only when necessary for brand perception comprehension.

A previous important note should be addressed before discussing the results. Although the analysis methods rely on recent advances in artificial intelligence, more specifically in machine learning, an established issue still holds: reverse inference. A common approach in cognitive neuroscience is to conclude about the participation of a psychological process because a specific brain region activates (or deactivates). That would be sustainable if that brain region, and no one more, supports the psychological process (Poldrack, 2006). However, such scenarios are uncommon. To strengthen the reverse inference approach, one may use meta-analysis that previously studied the range of psychological processes supported by the brain region and/or resort to multi-voxel pattern analysis (MVPA), which largely are machine learning methods to support the findings (Poldrack, 2008; Nathan and Del Pinal, 2017). Nonetheless, certain authors claim that reverse inference is not a gross fallacy and that their findings may still be helpful in scientific progress, depending on the analysis circumstances (Hutzler, 2014). So, the results here are not considered to be single brain regions but networks.

IC2 is an input that encompasses information important for correctly making predictions on BP, BI, and O categories. Its SHAP values are high and positive in these three cases but negative in P (*cf.* Figures 5, 6). Its path-weights have a similar pattern (*cf.* Figure 3). IC6 is also important for brands. The SHAP values are positive for BP, BI, and P but negative for O, and their path-weights replicate this pattern. Both brands’ path-weights and SHAP values are high for IC13. The path-weights are positive for BP, BI, and P (*cf.* Figure 4), and the SHAP values are positive for BP and BI (*cf.* Figures 5, 6). IC15 distinguishes brands more sharply from non-brands. It seems to be the IC that most contributes to this chasm. Their path-weights are positive for BP and BI but include negatives for O and P (*cf.* Figure 4). In addition to the signals, O and P path-weights negative magnitudes are very high (*cf.* Supplementary Tables S3, S4). The SHAP values are coherent with this scenario. They have high positive magnitudes for BP and BI and high (O)-to-moderate (P) negative magnitudes for brand stimuli (*cf.* Figures 5–7). IC47 is another important feature in correctly predicting BP and BI. Its SHAP values are high and positive (*cf.* Figure 5). Their path-weights that survived pruning are as follows: IC47 → h5 → BP 0.257, IC47 → h8 → BP 0.252 (*cf.* Supplementary Table S1); IC47 → h5 → BI 0.157, IC47 → h8 → BI 0.201 (*cf.* Supplementary Table S2); IC47 → h4 → O -0.155, IC47 → h8 → O 0.182 (*cf.* Supplementary Table S3); and IC47 → h4 → P -0.194, IC47 → h5 → P 0.196 (*cf.* Supplementary Table S4). These results suggest that IC2, IC6, IC13, IC15, and IC47 are important in brand global perception.

IC2, IC6, IC13, IC15, and IC47 exhibit a complex pattern of activations and deactivations. However, a joint appreciation of this ensemble reveals the participation of the occipital pole (IC6 and IC15) and the supracalcarine cortex (IC13), which are known to have a function in visual processing, for example, processing low-level elements as colors, shapes, distance, and depth. This is because it includes the primary visual cortex and object location and recognition in neighborhood regions (Rehman and Al Khalili, 2023). Another brain region known to contribute to visual processing present in these five ICs is the lateral occipital cortex (LOC). However, the participation of the LOC is rather complex. In IC2, the left superior and inferior LOC have activations, and the right LOC has a deactivation. In IC6, the left superior LOC has a deactivation. In IC13, there is an activation in the left hemisphere in the inferior LOC, spanning temporooccipital areas. Finally, IC15 encompasses a deactivation in the right inferior LOC. These results reveal an extensive participation of the LOC in brand perception. Grill-Spector et al. (2001) characterize the LOC as a high-level complex involved in object recognition, a visual associative brain area. High level in the sense that the object formation in the mind/brain is independent of low-level characteristics. These authors propose a hierarchical process, spatially oriented from LOC’s caudal zones to the rostral, with increased shape and object definition, inscribed in a general-purpose mechanism devoted to object recognition but composed of category-specific zones. IC2 includes the participation of the fusiform gyri, IC13 regions of temporooccipital zones, and IC47 of caudal parts of the inferior temporal gyri. These brain areas also process high-level visual information, i.e., they are domain-specific and contribute to perceptual expertise (Jacques et al., 2016; Weiner and Zilles, 2016).

Recently, Op de Beeck et al. (2019) proposed a theoretical framework based on three pillars to explain the emergence of category-specific areas in the visual system:(i) the pre-existence of selectivity for the stimulus category, (ii) appropriate processing hierarchy in the visual system, and (iii) category-specific connectivity to non-visual brain areas. There are already category-specific areas identified for faces, which include the fusiform face area (Kanwisher et al., 1997), words, bodies, hands, scenes, tools, and numerals. So far, there is no evidence of a brand logotypes-specific area, but considering the requirement is “images of ecologically meaningful categories” (Op de Beeck et al., 2019) for long, many theories and examples may be taken from marketing to support the clause. This is the case of the role brands have in self-construal (Sirgy, 1982; Belk, 1988; Elliott and Wattanasuwan, 1998; Escalas and Bettman, 2005; Swaminathan et al., 2007); brands are used to protect and repair the self (Sivanathan and Pettit, 2010), provide information about other individuals (Berger and Heath, 2007), promote the formation of social groups and their cohesiveness in the long-range (Reingen et al., 1984; Muñiz and O'Guinn, 2001; Cova and Cova, 2002; Veloutsou and Moutinho, 2009), and structure social relationships (Ahuvia, 2005). Hence, brands have a role in subjects’ self-construal or integration into social groups. Brands, represented by their logos, have a role in social navigation, and this image category is ecologically meaningful. Concerning the second pillar, it was already shown in the present study that the extensive participation of regions involved in hierarchical visual processing aims to identify the target object. Such regions largely participate in BP and BI impression formation. Finally, regarding the third pillar, the connection between category-specific brain areas in the visual system and non-visual brain areas is addressed in the next paragraph.

The IC6 network has a deactivation in superior temporal areas (right and left planum temporale and Heschel’s gyri) and another deactivation in the central pole. These non-visual areas exhibit deactivations. However, they are functionally connected in the same network, including the visual system in general and category-specific visual associative areas in particular. Nonetheless, IC4, IC37, and IC40, networks whose path-weights and SHAP values differentiate BP from the remaining stimuli, exhibit large activations and deactivations beyond the visual regions. IC4 has deactivations in the temporal poles and anterior prefrontal cortex. IC37 encompasses activation and deactivations in the dorsal banks of the Sylvian fissure, extending dorsally until the longitudinal fissure. Conspicuously, IC37 also includes parts of the LOC and the occipital pole in its network, i.e., again the connection between visual associative areas with non-visual regions, as previewed in the third pillar in Op de Beeck et al. (2019). IC40 includes an activation in rostral areas of the medial prefrontal cortex, spanning the paracingulate gyrus, the anterior division of the cingulate gyrus, the superior frontal gyrus, the frontal pole, and the frontal medial cortex. In addition, IC78, with a large activation in the frontal pole, is another non-visual region activated for BI. Hence, these results identify the components and functionally characterize a brain network involved in brand perception, which encompasses several sequential stages, starting with low-level decoding in caudal structures of the visual system, then hierarchically passing to high-level object identification in the extrastriate and temporooccipital regions, probably with domain-specific zones doing fine processing in the latter, and, finally, connecting to non-visual areas for further processing.

Tyler and Moss (2001) refused “concept containers” in the brain and proposed that conceptual knowledge representation has a distributed schema. When a concept is recruited, a pattern of activations of semantic features orchestrates into the concept formation. Visual object recognition depends on a hierarchical pathway starting in the occipital cortex and ventrally extending to the temporal lobe (Carlson et al., 2014). Along the route, the nature of the conceptualization process is increasingly complex (Tyler et al., 2013), but it remains feature-based (Taylor et al., 2011). The theory is extended further. Object understanding relies on an evolving flux starting with low-level visual input in ventral occipital areas, passing by categorical organization in intermediate ventral brain structures, and culminating in specific conceptual representations in structures of the anterior temporal cortex (Clarke and Tyler, 2015). Ayzenberg and Behrmann (2022) question that the ventral visual pathway processes the global object shape, which fueled the discussion in the academic community (Ayzenberg and Behrmann, 2023; Goodale and Milner, 2023). From their perspective, the role of the dorsal visual pathway and the ventral pathway processes objects’ local features. IC2, IC6, IC13, IC15, and IC47 depict all these dynamics. IC2 seems to assume a pivotal role in these theories of meaning attribution in visual-based information, as it encompasses structures in the ventral pathway and the dorsal pathway. If one considers a brand as a memeplex (Blackmore, 1998; Barnett, 2002), then it fits into these theories of object understanding, as brand memes are suited to be considered a basic-level concept or local features. The global shape would be the brand itself.

This system devoted to brand perception, recruiting the ventral visual pathway (Goodale and Milner, 1992; Milner and Goodale, 2008), was initially described in the study by Marques dos Santos and Moutinho (2015). However, the supporting data were essentially correlational and not a machine learning-based model where performance is tested for validation, as in the present study.

### 4.3 Is brand preference encoded in visual areas?

IC4, IC37, and IC40 are networks important for correct predictions of BP, either because they have high positive path-weights or high positive SHAP values, while these values are low or not very relevant in the remaining categories (because they are pruned). Hence, these networks are candidates for supporting processes that are dominant for BP.

IC4’s path-weights to BP that survived pruning are positive. They are high in magnitude and involve the hidden nodes 1, 5, and 7, which contribute most to BP decisions (*cf.* Figure 9), although hidden node 7 has a negative polarity with lesser magnitude. IC4 has positive and negative path-weights in the remaining categories. Concerning the SHAP values, IC4 is high (ranking second) and positive for BP, almost null for BI, and highly negative for O and P. Regarding IC37, it has positive path-weights involving the hidden nodes 5 and 7 (IC37 → h5 → BP 0.195; IC37 → h7 → BP 0.082). BI has no afferences coming from IC37, and the path-weights reaching O and P coming from IC37 are all negative. The SHAP value is positive for BP, null for BP and P, and negative for O. IC40’s path-weights are similar to IC37: IC40 → h5 → BP 0.099 and IC40 → h7 → BP 0.174. They are negative for BP (hidden nodes 2 and 7), O (hidden node 10), and P (hidden node 7). The SHAP values are positive for BP, almost null for O and P, and negative for BI. One may then conclude that IC4, IC37, and IC40 represent brain networks that mostly do BP processing.

IC4 exhibits a complex pattern of activations and deactivations in visual and visual associative areas (*cf.* Table 3): activation in the cuneal cortex, activation and deactivations in areas belonging to the inferior and superior LOC, and bilateral deactivations in the fusiform cortices. IC37 also has an activation in the superior LOC, which extends from the occipital pole. These results reveal that brain structures in visual and visual associative areas contain information that may be used to correctly classify preferred brands (BP). Such findings, however, suggest a brain process composed of networks in tandem working solely for BP processing. Consequently, it seems that there is no pipeline that decides if one prefers a brand or is indifferent to it. On the contrary, it seems that preferred and indifferent brands are screened early in the visual stream, and such meanings, preference and indifferent, are assigned (“tagged”) to the stimuli long before the information reaches the prefrontal cortex, which the brain structure accepted as a decision-making processor many times (Grabenhorst and Rolls, 2011). One may then speculate that the “decision” about a preferred brand takes place in visual and visual associative areas, which is more of a “tagging” process.

These results find support in scientific literature. Years ago, in one of the earliest attempts to use machine learning methods to analyze fMRI data, Hanson et al. (2004) could extract information from the fusiform gyri to classify correctly houses, chairs, scissors, shoes, and bottles, even with a tiny sample of subjects. Similarly, the present study reconfirms the findings by Marques dos Santos et al. (2014), adding increased accuracy, brain networks supporting BP detail and complexity, and supporting methodological procedures. Studying the brain substrates of national brands vs. own-labels, Marques dos Santos et al. (2016) found “the most surprising finding is that visual and visual associative areas are involved in the contrasts between branded products marked with switched prices and marked with real market prices,” i.e., visual and visual associative areas, such as the lingual gyrus, lateral occipital cortex, occipital fusiform gyrus, and occipital pole, have a role in the association between a brand and its market price. The product adds extensive activations in the same areas, plus the temporal occipital fusiform gyrus. It seems then that visual and visual associative areas contain important information for classifying visual stimuli and brands. In the latter case, its meaningful characteristics seem to be decoded in these ventral caudal regions of the brain.

These results may suggest alternative interpretations of past experiments. For example, Deppe et al. (2005) found a “winner-take-all” effect when target beer and coffee brands were contrasted with diverse others. When subjects were stimulated with their “first-choice brand,” their brains exhibited a complex system of activations and deactivations. Interestingly, the authors report these activations and deactivations in several areas of the posterior parietal and occipital cortices for the “first-choice brand.” There is a conspicuous similarity between the concept of “first-choice brand” and “brand|preferred”; hence, one may parallel the two systems of activations and deactivation, emphasizing those in visual and visual associative areas. In light of the present study’s findings, maybe the “first-choice brands” are signaled in visual associative areas because they are preferred in subjects’ minds, generating activations and deactivations in those brain areas.

More studies have reported the participation of visual areas in fMRI studies involving preferred brands. For example, Santos et al. (2011) compared preferred brands with indifferent brands and fictitious logos. Using an analysis method combining ICA and GLM, these authors could identify an IC that correlates significantly more with preferred brand stimulation than the two others. The IC identified encompasses large activations in visual and visual associative areas. In a paradigm aiming to study implicit and explicit brand impression formation, Santos et al. (2012) report the participation of the fusiform gyri in the conjunction analysis, i.e., in both situations of impression formation. One important IC resulting from the ICA analysis also reports the activation of the fusiform gyri. Hence, the participation of visual and visual associative areas in brand perception seems to be recurrent in Consumer Neuroscience studies since the beginning of the discipline. However, an increasing number of studies find different processes for preferred versus indifferent brands located in visual and visual associative areas, i.e., the differentiation between the types of brands is suggested to be encoded in early visual processing.

### 4.4 Model interpretation in the hidden nodes

One important purpose of the path-weights analysis is to study how the network training dynamics aggregates specific processes in the nodes of the hidden layer (*cf.* Figure 4). In addition, the SHAP values are calculated between the hidden layer and the output, informing which hidden nodes most contribute to decisions in each category (*cf.* Figures 9, 10; Supplementary Figure S5).

Considering brands only, i.e., BP and BI, it is possible to summarize the important SHAP values as follows:

- BP: hidden nodes 1 and 5 are very important, and hidden node 10 is important; hidden nodes 7 and 8 are negative;
- BI: hidden node 1 is very important, and hidden nodes 5 and 7 are important; hidden nodes 2 and 8 are highly negative, and hidden node 10 is negative.

Hidden node 1 is highly positive both for BP and BI. Among its afferents, one may find IC1, IC2, IC4, IC8, IC15, and IC78 (weights greater than 0.200, *cf.* Supplementary Table S5). Some of these ICs have already been identified as representing networks that contribute to global brand appraisal. This is the case of IC2 and IC15 (*cf.* Figure 11). Nonetheless, hidden node 1 also encompasses contributions from ICs that are considered important for specific brand category classification. This is the case of IC4 for BP and IC78 for BI (*cf.* Figure 12). However, hidden node 1 is not important only for brand classification. It is also important for O. Therefore, one may not conclude that it concentrates on a global brand appraisal, but the results suggest that this node concentrates information on non-human stimuli. Conspicuously, both IC2 and IC15, and IC4 and IC78 form a complex network of networks following the requirements previewed by Op de Beeck et al. (2019): it encompasses components from the visual and visual associative areas (IC2, IC4, and IC15) and from other non-visual system areas for further processing (IC4 and IC78 involving the frontal medial cortex and the dorso lateral prefrontal cortex, respectively). All these brain regions contribute information to hidden node 1 processing (among others, though).

Regarding brands, hidden node 5 is similar to hidden node 1. Just the weights’ magnitudes are reversed. Hidden node 5 weight is higher for BP than for BI. IC4, IC6, IC9, IC13, IC15, IC16, IC37, IC47, and IC78 are among those with higher weights, besides IC40. IC6, IC13, IC15, and IC47 are ICs already identified in the process of global brand appraisal (*cf.* Figure 11). IC4, IC37, and IC40 were already identified as being important for BP classification (*cf.* Figure 12), and thus, with no surprise, they contribute to the hidden node that mostly weights for BP classification. Again, one may not conclude that hidden node 5 is exclusive of brand perception, as it also has a high weight for classification in the P bin (the second highest; *cf.* Supplementary Table S6). Nonetheless, these results support the inference that hidden node 5 is involved in more “organic” stimuli, with an emphasis on brands|preferred, because it also participates in the processing of human faces (P). A common denominator is brand anthropomorphizing, whose evidence and impacts on consumer behavior have been studied for a long time (Aggarwal and McGill, 2007; Brown, 2010; Aggarwal and McGill, 2012). Similar to hidden node 1, hidden node 5 concentrates a complex pattern of networks of networks, again following the theory outlined by Op de Beeck et al. (2019). There is the participation of visual and visual associative regions (IC4, IC6, IC13, IC15, and IC47) and other regions outside the visual system as the anterior prefrontal cortex (IC4), the precentral gyrus and the central opercular cortex (IC37), or the dorso lateral prefrontal cortex (IC40), the latter supporting classification in BP.

Hidden nodes 7 and 10 have opposite roles respecting BP and BI classification. In both cases, however, they also contribute positively to P, which means what was said in the previous paragraph about anthropomorphisms still applies here. IC6, IC13, and IC15 were identified as positive contributors to the global brand appraisal (*cf.* Figure 11) and also have important positive contributions to the hidden node 10. All ICs identified for BP-specific classification, i.e., IC4, IC37, and IC40 (*cf.* Figure 12), have positive contributions to this hidden node, but none of the ICs identified for BI classification has afferences here (*cf.* Supplementary Table S6) because they were pruned. The substrate of the natural neural network replicates hidden node 5, i.e., involving the participation of visual and visual associative areas associated with non-visual areas such as the precentral gyrus and the central opercular cortex.

In hidden node 7, all the ICs identified for BP-specific classification contribute negatively here. Yet, none of the ICs identified for BI classification, i.e., IC57 and 78 (*cf.* Figure 12), have contributions to hidden node 7 because they were pruned. According to Supplementary Table S6 and Supplementary Figure S2, the IC with positive weights afferent to hidden node 7 and high positive SHAP values is IC15 only. Thus, one may admit that the contributions to BI classification in the hidden node 7 also get support on negative afferences by ICs containing deactivations.

## 5 Conclusion, limitations, application, and further work

The study modeled brain data related to brand perception. The model is sound in neuroscientific terms. The initial participation of the primary visual system, with successive layers of visual processing aiming at meaning attribution, was expected and is followed. From this point on, the processed stimulus reaches other parts of the brain for further processing, sometimes integrating such processing for a complete stimulus description. For example, the participation of anterior areas of the prefrontal cortex in brand appraisal is either preferred or indifferent (Santos et al., 2011). However, the most important finding here is that early processing stages, still in the visual system, contain information that suggests a split between processing brands|preferred from brands|indifferent. Results from previous studies have already made that suggestion. The present one reveals such networks and their participation in brands|preferred and brands|indifferent classification. The evidence produced does not have a correlational basis. It results from the predictions of a model, which emerged from data with no prior theory influences, trained in a different cohort of subjects to ensure generalizability. Hence, the finding that brand preference exists in the visual system is robust in the sense that it is not merely correlational, and the model creation was prevented by influences of previous theories.

However, the findings deserve a word of warning. The temporal dimension of the data acquisition technique used, fMRI, is not optimal. The TR equals 2.5 s. Many processes arise in the brain during this time gap, and fMRI is not suited to sequence them. Although fMRI strictly informs that there is an activation in the lateral occipital cortex, for example, it does not inform as accurately when it happens. Other neuroscientific techniques with high temporal resolution, such as EEG (electroencephalography), are better suited and should be employed for such purposes.

Machine learning methods are evolving remarkably nowadays, notably in terms of knowledge extraction. One may expect that the analysis pipeline depicted in Figure 1 will be improved soon. This does not mean that the methods, results, and findings reported here are not valid. They are valid, but in light of the current state of knowledge, this should regulate the interpretations of all these aspects of the article.

The theory discussed here may be used to interpret other situations related to brand perception, especially when preferred brands are involved. For example, the concepts of “first-choice brands” (Deppe et al., 2005) and “brand|preferred” overlap with the concepts of “consideration sets” or “evoked sets” in marketing. A consideration set is the group of brands in the consumer’s mind, which are the first to be prompted, i.e., the “first-choice brand.” For a long time now, the question has remained whether consideration sets exist or not (Petrof and Daghfous, 1996), despite several attempts over the years to screen such lauded group of brands (Nordfält et al., 2004; Hauser et al., 2010; Hauser, 2014; Bremer et al., 2017). A significant hurdle is that consideration sets may be behind consumers’ awareness, i.e., this is the kind of information consumers use in everyday life but behind consciousness. Supposedly, the information behind consciousness may not be addressed by deliberative reasoning, which precludes traditional data acquisition methods in marketing, such as surveys and focus groups, because they position the consumer respondent in a deliberative plane, missing ecological validity, as consumers do not go to supermarkets optimizing utility (Fitzsimons et al., 2002; Lee et al., 2009; Cisek and Kalaska, 2010). Consumers use frugal heuristics (Gigerenzer and Gaissmaier, 2011). Nonetheless, the theory produced in the present study supports the existence of consideration sets. It paves the way for identifying the consideration set elements objectively and not requiring explicit verbalizations from consumers, i.e., surpassing the explicit-deliberative plane. One must scan the consumer’s brain while being stimulated with brand logos and use the model to screen those “tagged” in visual areas of the brain as brand|preferred.

## Data availability statement

The raw data supporting the conclusions of this article will be made available by the authors, without undue reservation.

## Ethics statement

Ethical approval was not required for the studies involving humans because the study is a re-analysis of more than one decade-old fMRI data. The original consents were already destroyed, and all the subjects’ data as well. Only the anonymized raw data files exist. The studies were conducted in accordance with the local legislation and institutional requirements. The participants provided their written informed consent to participate in this study.

## Author contributions

JPMS: Writing – review & editing, Writing – original draft, Visualization, Software, Project administration, Methodology, Funding acquisition, Formal analysis, Data curation, Conceptualization. JDMS: Writing – original draft, Visualization, Software, Formal analysis, Data curation.

## Footnotes

^1^ https://nist.mni.mcgill.ca/atlases/

^2^ https://github.com/shap/shap

^3^ https://pytorch.org
