# Supplementary material for: Explainable artificial intelligence (xAI) in neuromarketing/consumer neuroscience: an fMRI study on brand perception
Source: Front Hum Neurosci. 2024 Mar 22;18:1305164. doi: 10.3389/fnhum.2024.1305164 (PMC10995351; doi:10.3389/fnhum.2024.1305164)
Supplement: Supplementary file 1 [file Data_Sheet_1.docx]

Supplementary Materials

# Exploration of the ANN’s Architecture

The initial ANN’s architecture strategy is to be frugal at the beginning and introduce complexity afterwards with parsimony. The objective is not accuracy itself. The level of accuracy required is the sufficient to ensure that the process extracts information relevant to correct classification comfortably above mere randomness. Once this level is achieved, there would already be a model from which it is possible to extract information.

The starting data consists of 125 input nodes and 4 output nodes. Considering a single hidden layer, the number of weights equals 129n, where n is the number of hidden nodes. A current rule of thumb is that there should be ten instances per parameter. In this case, acknowledging that there are 2399 instances, the first approach to the number of hidden nodes for proper training would be 2. If there are two hidden layers, assuming the same number of hidden nodes in each one, the number of weights equals n(129+n). In such a case, the number of hidden nodes would be 2 again. Of course, this is just a rule of thumb. How the data represents the problem’s complexity judges the minimum data for proper training. In any case, the first approaches yielded by the rule-of-thumb signals caution, suggesting that if the model is complex, i.e., if the network has several hidden layers and hidden nodes per layer, there may be severe problems with training it.

Three scenarios are explored: the ANN has one, two, and three hidden layers. If the ANN has two or more layers, the network has an equal number of hidden nodes in all layers. Then, the total number of correct predictions is calculated varying the number of hidden nodes from one to 20, as depicted in Supplementary Figure 1. From three hidden nodes on, both the architectures encompassing 2 and 3 hidden layers decrease in correct predictions, which means that more hidden layers and more hidden nodes signify lesser model performance. The results confirm the suggestion that the number of training instances is insufficient to support complex network architectures. Hence, the ANN has only one hidden layer.

Because the single-layer architecture performance remains stable after three hidden nodes (cf. Supplementary Figure 1), the exploration for one hidden layer is extended to 500 hidden nodes. With 50 hidden nodes, the total correct predictions drop to 529, with 100 hidden nodes to 510, and with 500 hidden nodes, it drops to 490, meaning that performance decays slowly but consistently.

The strategy is to select the most frugal network, as stated above. The procedure implemented for choosing the number of hidden nodes is to start with three and stop when the pruned network has at least one hidden node completely severed, i.e., there is at least one hidden node that has no connections from any of the input nodes, neither connections to the output nodes. Such is achieved with 10 hidden nodes, as demonstrated in Supplementary Tables 1 to 6. The underlying reason is that this network has more than enough hidden nodes to ensure correct predictions, thus dismissing more complex architectures. Hence, the ANN selected has a single hidden layer containing 10 hidden nodes.


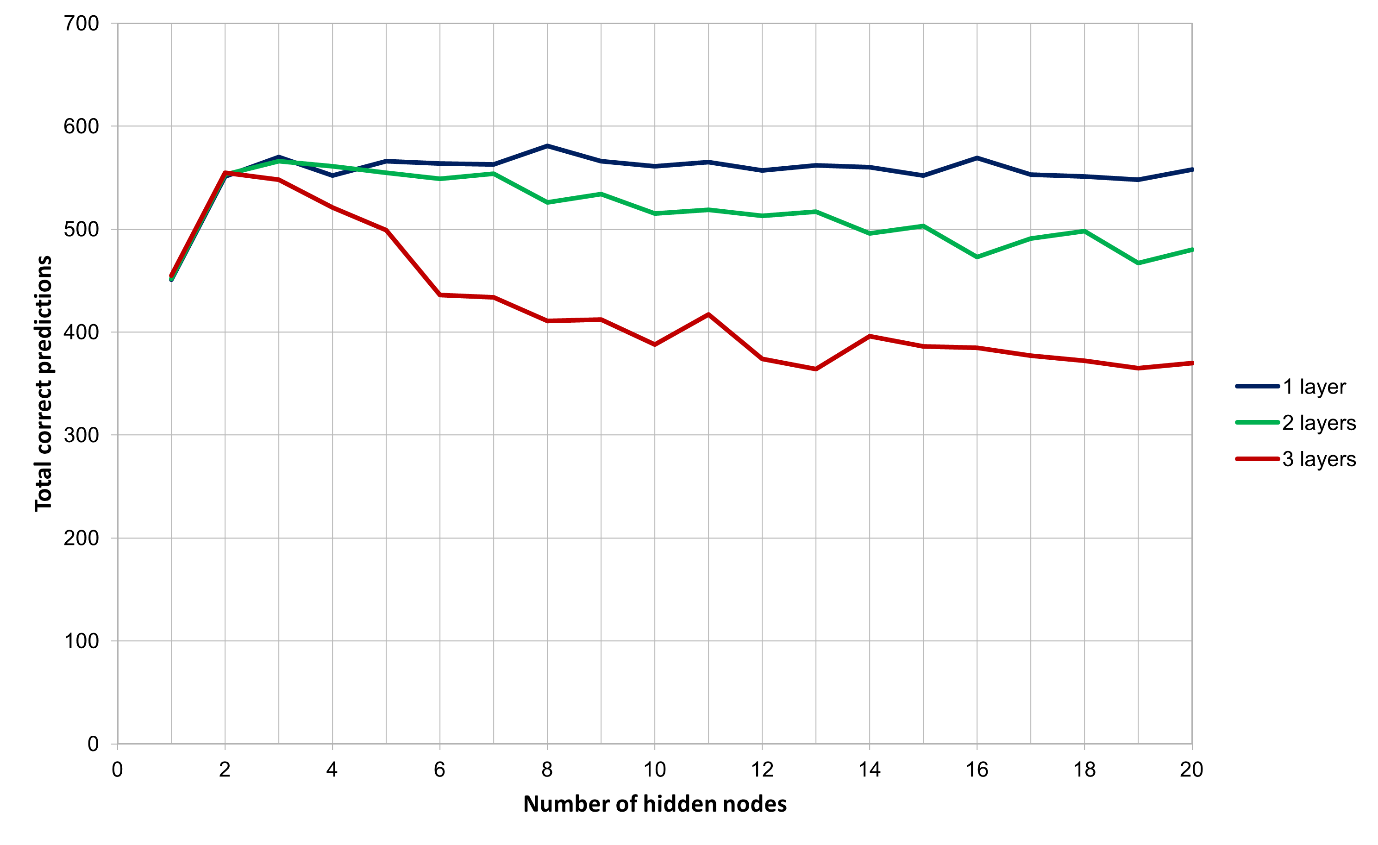


Supplementary Figure 1. Total correct predictions of each model as a function of the number of hidden layers (one, two, and three) and the number of hidden nodes per layer. If two or more layers, the number of hidden nodes is the same in all layers.

# Path-Weights Calculation and Pruning

Supplementary Table 1 contains the values of the path-weights that survived pruning for category BP (brands|preferred). The symbol “---” means the path-weight was pruned. For example, the path-weight IC1 (input) → Hidden node 2 → BP (output) is pruned, and, therefore, no magnitude is presented. The table cells are color-coded. Darker red cells mean higher path-weights, i.e., connections that favor the information fed in the respective input and, therefore, contribute more to the final decision (model predictions).

Supplementary Table 1. Path-weights corresponding to the connections that take to output BP, which survived pruning. Inputs are in rows. Darker red means higher path-weights values.

| **IC** | **Hidden nodes** | | | | | | | | | |
| --- | --- | --- | --- | --- | --- | --- | --- | --- | --- | --- |
|  | **1** | **2** | **3** | **4** | **5** | **6** | **7** | **8** | **9** | **10** |
| 1 | 0.2632 | --- | --- | --- | -0.2847 | --- | 0.1686 | --- | --- | -0.1600 |
| 2 | 0.4076 | --- | --- | --- | --- | --- | 0.2192 | --- | --- | -0.1737 |
| 3 | -0.2501 | --- | --- | --- | -0.1500 | --- | -0.1191 | --- | --- | --- |
| 4 | 0.1648 | --- | --- | --- | 0.2333 | --- | 0.2600 | --- | --- | --- |
| 5 | --- | --- | --- | --- | -0.5148 | --- | 0.2579 | --- | --- | -0.1583 |
| 6 | --- | --- | --- | --- | 0.3205 | --- | --- | 0.1551 | --- | 0.1190 |
| 7 | --- | --- | --- | --- | --- | -0.1144 | 0.1083 | -0.4786 | --- | --- |
| 8 | 0.1750 | --- | --- | --- | --- | --- | 0.1032 | --- | --- | --- |
| 9 | -0.2700 | --- | --- | --- | 0.3120 | --- | -0.2273 | --- | --- | 0.1526 |
| 10 | --- | --- | --- | --- | --- | --- | --- | 0.2345 | --- | --- |
| 11 | --- | --- | --- | --- | --- | --- | --- | -0.2094 | --- | --- |
| 12 | --- | --- | --- | --- | -0.3937 | --- | 0.1327 | --- | --- | -0.1685 |
| 13 | --- | --- | --- | --- | 0.2615 | --- | --- | --- | --- | --- |

Supplementary Table 1. (cont.)

| **IC** | **Hidden nodes** | | | | | | | | | |
| --- | --- | --- | --- | --- | --- | --- | --- | --- | --- | --- |
|  | **1** | **2** | **3** | **4** | **5** | **6** | **7** | **8** | **9** | **10** |
| 14 | -0.5391 | --- | --- | --- | --- | --- | -0.2075 | --- | --- | 0.0841 |
| 15 | 0.1890 | --- | --- | --- | 0.1788 | --- | -0.1374 | --- | --- | 0.1020 |
| 16 | --- | --- | --- | --- | 0.2222 | --- | --- | -0.3435 | --- | --- |
| 17 | --- | --- | --- | --- | -0.1742 | --- | --- | --- | --- | --- |
| 18 | --- | --- | --- | --- | 0.1176 | --- | --- | --- | --- | --- |
| 19 | 0.1432 | --- | --- | --- | --- | --- | --- | -0.3246 | --- | --- |
| 20 | --- | --- | --- | --- | -0.2072 | --- | --- | 0.1009 | --- | --- |
| 21 | --- | --- | --- | --- | 0.1140 | --- | --- | -0.1668 | --- | --- |
| 22 | --- | --- | --- | --- | --- | --- | --- | 0.1258 | --- | --- |
| 23 | --- | --- | --- | --- | --- | --- | 0.1543 | --- | --- | --- |
| 24 | --- | --- | --- | --- | --- | --- | --- | 0.1812 | --- | --- |
| 25 | --- | --- | --- | --- | --- | --- | --- | 0.2029 | --- | --- |
| 26 | --- | --- | --- | --- | --- | --- | --- | --- | --- | --- |
| 27 | --- | --- | --- | --- | 0.0882 | --- | --- | 0.1874 | --- | --- |
| 28 | --- | --- | --- | --- | 0.2093 | --- | --- | --- | --- | --- |
| 29 | --- | --- | --- | --- | 0.1418 | --- | --- | 0.1333 | --- | --- |
| 30 | --- | --- | --- | --- | --- | --- | --- | --- | --- | --- |
| 31 | --- | --- | --- | --- | --- | --- | --- | 0.1574 | --- | --- |
| 32 | -0.1707 | --- | --- | --- | --- | --- | --- | --- | --- | --- |
| 33 | --- | --- | --- | --- | --- | --- | -0.1491 | --- | --- | --- |
| 34 | -0.1821 | --- | --- | --- | --- | --- | --- | --- | --- | --- |
| 35 | --- | --- | --- | --- | --- | --- | --- | --- | --- | --- |
| 36 | --- | --- | --- | --- | --- | --- | --- | -0.1925 | --- | --- |
| 37 | --- | --- | --- | --- | 0.1951 | --- | 0.0817 | --- | --- | --- |
| 38 | --- | --- | --- | --- | -0.1519 | --- | --- | --- | --- | --- |
| 39 | --- | --- | --- | --- | --- | --- | --- | --- | --- | --- |
| 40 | --- | --- | --- | --- | 0.0986 | --- | 0.1736 | --- | --- | --- |
| 41 | --- | --- | --- | --- | 0.1175 | --- | --- | --- | --- | --- |
| 42 | --- | --- | --- | --- | --- | --- | --- | 0.1785 | --- | --- |
| 43 | --- | --- | --- | --- | --- | --- | --- | 0.1415 | --- | --- |
| 44 | --- | --- | --- | --- | --- | --- | --- | --- | --- | --- |
| 45 | --- | --- | --- | --- | --- | --- | --- | --- | --- | --- |
| 46 | --- | --- | --- | --- | 0.1391 | --- | 0.0993 | --- | --- | --- |
| 47 | --- | --- | --- | --- | 0.2572 | --- | --- | 0.2522 | --- | --- |
| 48 | -0.1099 | --- | --- | --- | --- | --- | --- | 0.0933 | --- | --- |
| 49 | --- | --- | --- | --- | --- | --- | --- | --- | --- | --- |
| 50 | --- | --- | --- | --- | --- | --- | --- | --- | --- | --- |
| 51 | --- | --- | --- | --- | --- | --- | --- | --- | --- | --- |
| 52 | --- | --- | --- | --- | --- | --- | --- | --- | --- | --- |
| 53 | --- | --- | --- | --- | --- | --- | --- | --- | --- | --- |
| 54 | --- | --- | --- | --- | --- | --- | --- | --- | --- | --- |
| 55 | --- | --- | --- | --- | --- | --- | --- | --- | --- | --- |
| 56 | -0.1482 | --- | --- | --- | --- | --- | --- | --- | --- | --- |
| 57 | 0.1142 | --- | --- | --- | --- | --- | --- | --- | --- | --- |
| 58 | --- | --- | --- | --- | --- | --- | --- | 0.1517 | --- | --- |
| 59 | 0.1185 | --- | --- | --- | -0.1118 | --- | --- | -0.1950 | --- | --- |
| 60 | --- | --- | --- | --- | 0.1057 | --- | --- | --- | --- | --- |
| 61 | --- | --- | --- | --- | --- | --- | --- | --- | --- | --- |
| 62 | --- | --- | --- | --- | --- | --- | -0.1127 | 0.1143 | --- | --- |
| 63 | --- | --- | --- | --- | --- | --- | --- | --- | --- | --- |
| 64 | --- | --- | --- | --- | 0.1025 | --- | --- | --- | --- | --- |
| 65 | --- | --- | --- | --- | --- | --- | --- | --- | --- | --- |
| 66 | --- | --- | --- | --- | --- | --- | 0.1008 | --- | --- | --- |
| 67 | --- | --- | --- | --- | 0.1375 | --- | --- | -0.1420 | --- | --- |
| 68 | --- | --- | --- | --- | --- | --- | 0.0931 | --- | --- | --- |
| 69 | --- | --- | --- | --- | --- | --- | --- | --- | --- | --- |
| 70 | --- | --- | --- | --- | --- | --- | --- | --- | --- | --- |
| 71 | --- | --- | --- | --- | --- | --- | --- | --- | --- | --- |
| 72 | --- | --- | --- | --- | --- | --- | --- | --- | --- | --- |
| 73 | --- | --- | --- | --- | --- | --- | --- | --- | --- | --- |
| 74 | --- | --- | --- | --- | --- | --- | --- | --- | --- | --- |
| 75 | --- | --- | --- | --- | --- | --- | --- | --- | --- | --- |
| 76 | --- | --- | --- | --- | 0.1022 | --- | --- | -0.1415 | --- | --- |
| 77 | --- | --- | --- | --- | --- | --- | --- | --- | --- | --- |
| 78 | 0.2064 | --- | --- | --- | --- | --- | --- | --- | --- | --- |
| 79 | --- | --- | --- | --- | -0.1434 | --- | --- | --- | --- | --- |

Supplementary Table 1. (cont.)

| **IC** | **Hidden nodes** | | | | | | | | | |
| --- | --- | --- | --- | --- | --- | --- | --- | --- | --- | --- |
|  | **1** | **2** | **3** | **4** | **5** | **6** | **7** | **8** | **9** | **10** |
| 80 | --- | --- | --- | --- | --- | --- | 0.1566 | -0.1160 | --- | --- |
| 81 | --- | --- | --- | --- | --- | --- | --- | --- | --- | --- |
| 82 | --- | --- | --- | --- | --- | --- | --- | --- | --- | --- |
| 83 | --- | --- | --- | --- | --- | --- | --- | --- | --- | --- |
| 84 | --- | --- | --- | --- | --- | --- | --- | --- | --- | --- |
| 85 | --- | --- | --- | --- | --- | --- | --- | 0.0887 | --- | --- |
| 86 | --- | --- | --- | --- | --- | --- | --- | --- | --- | --- |
| 87 | 0.1104 | --- | --- | --- | --- | --- | --- | -0.1452 | --- | --- |
| 88 | --- | --- | --- | --- | --- | --- | --- | --- | --- | --- |
| 89 | -0.1184 | --- | --- | --- | --- | --- | 0.0995 | 0.0901 | --- | --- |
| 90 | --- | --- | --- | --- | --- | --- | --- | --- | --- | --- |
| 91 | --- | --- | --- | --- | --- | --- | --- | --- | --- | --- |
| 92 | --- | --- | --- | --- | --- | --- | --- | --- | --- | --- |
| 93 | --- | --- | --- | --- | --- | --- | --- | 0.0892 | --- | --- |
| 94 | --- | --- | --- | --- | 0.1080 | --- | --- | --- | --- | --- |
| 95 | 0.1120 | --- | --- | --- | --- | --- | --- | 0.1027 | --- | --- |
| 96 | --- | --- | --- | --- | -0.1170 | --- | --- | 0.1383 | --- | --- |
| 97 | --- | --- | --- | --- | --- | --- | --- | --- | --- | --- |
| 98 | --- | --- | --- | --- | --- | --- | --- | --- | --- | --- |
| 99 | --- | --- | --- | --- | --- | --- | --- | --- | --- | --- |
| 100 | --- | --- | --- | --- | --- | --- | --- | --- | --- | --- |
| 101 | --- | --- | --- | --- | --- | --- | --- | --- | --- | --- |
| 102 | --- | --- | --- | --- | --- | --- | --- | --- | --- | --- |
| 103 | --- | --- | --- | --- | --- | --- | 0.1053 | --- | --- | --- |
| 104 | --- | --- | --- | --- | --- | --- | --- | --- | --- | --- |
| 105 | 0.1275 | --- | --- | --- | --- | --- | --- | --- | --- | --- |
| 106 | --- | --- | --- | --- | --- | --- | --- | --- | --- | --- |
| 107 | --- | --- | --- | --- | --- | --- | --- | --- | --- | --- |
| 108 | --- | --- | --- | --- | --- | --- | --- | --- | --- | --- |
| 109 | --- | --- | --- | --- | --- | --- | -0.1399 | 0.2020 | --- | --- |
| 110 | --- | --- | --- | --- | --- | --- | --- | --- | --- | --- |
| 111 | --- | --- | --- | --- | --- | --- | --- | --- | --- | --- |
| 112 | --- | --- | --- | --- | --- | --- | --- | 0.1083 | --- | --- |
| 113 | --- | --- | --- | --- | --- | --- | --- | --- | --- | --- |
| 114 | --- | --- | --- | --- | --- | --- | --- | --- | --- | --- |
| 115 | --- | --- | --- | --- | --- | --- | --- | -0.1175 | --- | --- |
| 116 | --- | --- | --- | --- | --- | --- | --- | --- | --- | --- |
| 117 | --- | --- | --- | --- | --- | --- | --- | --- | --- | --- |
| 118 | --- | --- | --- | --- | -0.1661 | --- | --- | --- | --- | --- |
| 119 | --- | --- | --- | --- | --- | --- | -0.1485 | --- | --- | --- |
| 120 | --- | --- | --- | --- | --- | --- | --- | --- | --- | --- |
| 121 | --- | --- | --- | --- | --- | --- | --- | --- | --- | --- |
| 122 | --- | --- | --- | --- | --- | --- | --- | --- | --- | --- |
| 123 | --- | --- | --- | --- | --- | --- | --- | 0.1270 | --- | --- |
| 124 | --- | --- | --- | --- | --- | --- | --- | --- | --- | --- |
| 125 | --- | --- | --- | --- | --- | --- | --- | --- | --- | --- |

Supplementary Table 2, Supplementary Table 3, and Supplementary Table 4 represent the path-weights values that survived pruning, respectively, for the category BI (brand|indifferent), O (objects), and p (people).

Supplementary Table 2. Path-weights corresponding to the connections that take to output BI, which survived pruning. Inputs are in rows. Darker red means higher path-weights values.

| **IC** | **Hidden nodes** | | | | | | | | | |
| --- | --- | --- | --- | --- | --- | --- | --- | --- | --- | --- |
|  | **1** | **2** | **3** | **4** | **5** | **6** | **7** | **8** | **9** | **10** |
| 1 | 0.4332 | --- | --- | --- | -0.1738 | --- | -0.1669 | --- | --- | 0.2289 |
| 2 | 0.6709 | --- | --- | --- | --- | --- | -0.2169 | --- | --- | 0.2485 |
| 3 | -0.4116 | -0.1910 | --- | --- | --- | --- | --- | --- | --- | --- |

Supplementary Table 2. (cont.)

| **IC** | **Hidden nodes** | | | | | | | | | |
| --- | --- | --- | --- | --- | --- | --- | --- | --- | --- | --- |
|  | **1** | **2** | **3** | **4** | **5** | **6** | **7** | **8** | **9** | **10** |
| 4 | 0.2712 | --- | --- | --- | 0.1425 | --- | -0.2574 | --- | --- | --- |
| 5 | 0.1289 | -0.2490 | --- | --- | -0.3144 | --- | -0.2552 | --- | --- | 0.2265 |
| 6 | --- | 0.1639 | --- | --- | 0.1957 | --- | --- | --- | --- | -0.1703 |
| 7 | --- | --- | --- | --- | --- | --- | --- | -0.3814 | --- | --- |
| 8 | 0.2880 | --- | --- | --- | --- | --- | --- | --- | --- | --- |
| 9 | -0.4443 | --- | --- | --- | 0.1905 | --- | 0.2249 | --- | --- | -0.2183 |
| 10 | --- | --- | --- | --- | --- | --- | --- | 0.1869 | --- | --- |
| 11 | --- | --- | --- | --- | --- | --- | --- | -0.1669 | --- | --- |
| 12 | --- | -0.2271 | --- | --- | -0.2404 | --- | -0.1313 | --- | --- | 0.2411 |
| 13 | --- | 0.2311 | --- | --- | 0.1597 | --- | --- | --- | --- | --- |
| 14 | -0.8874 | -0.3160 | --- | --- | --- | --- | 0.2054 | --- | --- | -0.1203 |
| 15 | 0.3110 | 0.3030 | --- | --- | --- | --- | 0.1360 | --- | --- | -0.1459 |
| 16 | --- | --- | --- | --- | 0.1357 | --- | --- | -0.2737 | --- | --- |
| 17 | --- | --- | --- | --- | --- | --- | --- | --- | --- | --- |
| 18 | --- | 0.1621 | --- | --- | --- | --- | --- | --- | --- | --- |
| 19 | 0.2357 | 0.2218 | --- | --- | --- | --- | --- | -0.2587 | --- | --- |
| 20 | --- | --- | --- | --- | -0.1265 | --- | --- | --- | --- | --- |
| 21 | --- | --- | --- | --- | --- | --- | --- | -0.1329 | --- | --- |
| 22 | --- | --- | --- | --- | --- | --- | --- | --- | --- | --- |
| 23 | --- | -0.1552 | --- | --- | --- | --- | -0.1527 | --- | --- | --- |
| 24 | --- | --- | --- | --- | --- | --- | --- | 0.1444 | --- | --- |
| 25 | --- | --- | --- | --- | --- | --- | --- | 0.1617 | --- | --- |
| 26 | --- | --- | --- | --- | --- | --- | --- | --- | --- | --- |
| 27 | -0.1671 | --- | --- | --- | --- | --- | --- | 0.1493 | --- | --- |
| 28 | --- | --- | --- | --- | 0.1278 | --- | --- | --- | --- | --- |
| 29 | --- | --- | --- | --- | --- | --- | --- | --- | --- | --- |
| 30 | --- | -0.1393 | --- | --- | --- | --- | --- | --- | --- | --- |
| 31 | --- | --- | --- | --- | --- | --- | --- | --- | --- | --- |
| 32 | -0.2809 | -0.1488 | --- | --- | --- | --- | --- | --- | --- | --- |
| 33 | --- | --- | --- | --- | --- | --- | 0.1475 | --- | --- | --- |
| 34 | -0.2997 | --- | --- | --- | --- | --- | --- | --- | --- | --- |
| 35 | --- | -0.1921 | --- | --- | --- | --- | --- | --- | --- | --- |
| 36 | --- | -0.1418 | --- | --- | --- | --- | --- | -0.1534 | --- | --- |
| 37 | --- | --- | --- | --- | --- | --- | --- | --- | --- | --- |
| 38 | --- | --- | --- | --- | --- | --- | --- | --- | --- | --- |
| 39 | --- | -0.1292 | --- | --- | --- | --- | --- | --- | --- | --- |
| 40 | --- | -0.2163 | --- | --- | --- | --- | -0.1718 | --- | --- | --- |
| 41 | --- | --- | --- | --- | --- | --- | --- | --- | --- | --- |
| 42 | --- | --- | --- | --- | --- | --- | --- | 0.1423 | --- | --- |
| 43 | -0.1251 | --- | --- | --- | --- | --- | --- | --- | --- | --- |
| 44 | --- | --- | --- | --- | --- | --- | --- | --- | --- | --- |
| 45 | --- | --- | --- | --- | --- | --- | --- | --- | --- | --- |
| 46 | --- | 0.2615 | --- | --- | --- | --- | --- | --- | --- | --- |
| 47 | --- | --- | --- | --- | 0.1571 | --- | --- | 0.2010 | --- | --- |
| 48 | -0.1808 | --- | --- | --- | --- | --- | --- | --- | --- | --- |
| 49 | --- | --- | --- | --- | --- | --- | --- | --- | --- | --- |
| 50 | --- | -0.2098 | --- | --- | --- | --- | --- | --- | --- | --- |
| 51 | --- | --- | --- | --- | --- | --- | --- | --- | --- | --- |
| 52 | --- | --- | --- | --- | --- | --- | --- | --- | --- | --- |
| 53 | --- | --- | --- | --- | --- | --- | --- | --- | --- | --- |
| 54 | -0.1380 | --- | --- | --- | --- | --- | --- | --- | --- | --- |
| 55 | --- | --- | --- | --- | --- | --- | --- | --- | --- | --- |
| 56 | -0.2439 | --- | --- | --- | --- | --- | --- | --- | --- | --- |
| 57 | 0.1880 | 0.1540 | --- | --- | --- | --- | --- | --- | --- | --- |
| 58 | --- | -0.1541 | --- | --- | --- | --- | --- | --- | --- | --- |
| 59 | 0.1950 | --- | --- | --- | --- | --- | --- | -0.1554 | --- | --- |
| 60 | --- | --- | --- | --- | --- | --- | --- | --- | --- | --- |
| 61 | --- | 0.2099 | --- | --- | --- | --- | --- | --- | --- | --- |
| 62 | --- | --- | --- | --- | --- | --- | --- | --- | --- | --- |
| 63 | --- | --- | --- | --- | --- | --- | --- | --- | --- | --- |
| 64 | -0.1505 | --- | --- | --- | --- | --- | --- | --- | --- | --- |
| 65 | --- | --- | --- | --- | --- | --- | --- | --- | --- | --- |
| 66 | 0.1257 | -0.1358 | --- | --- | --- | --- | --- | --- | --- | --- |
| 67 | --- | --- | --- | --- | --- | --- | --- | --- | --- | --- |
| 68 | --- | -0.1679 | --- | --- | --- | --- | --- | --- | --- | --- |
| 69 | --- | --- | --- | --- | --- | --- | --- | --- | --- | --- |

Supplementary Table 2. (cont.)

| **IC** | **Hidden nodes** | | | | | | | | | |
| --- | --- | --- | --- | --- | --- | --- | --- | --- | --- | --- |
|  | **1** | **2** | **3** | **4** | **5** | **6** | **7** | **8** | **9** | **10** |
| 70 | --- | --- | --- | --- | --- | --- | --- | --- | --- | --- |
| 71 | --- | --- | --- | --- | --- | --- | --- | --- | --- | --- |
| 72 | --- | --- | --- | --- | --- | --- | --- | --- | --- | --- |
| 73 | --- | --- | --- | --- | --- | --- | --- | --- | --- | --- |
| 74 | --- | --- | --- | --- | --- | --- | --- | --- | --- | --- |
| 75 | 0.1283 | --- | --- | --- | --- | --- | --- | --- | --- | --- |
| 76 | -0.1423 | --- | --- | --- | --- | --- | --- | --- | --- | --- |
| 77 | --- | --- | --- | --- | --- | --- | --- | --- | --- | --- |
| 78 | 0.3398 | 0.1665 | --- | --- | --- | --- | --- | --- | --- | --- |
| 79 | --- | -0.1310 | --- | --- | --- | --- | --- | --- | --- | --- |
| 80 | --- | --- | --- | --- | --- | --- | -0.1550 | --- | --- | --- |
| 81 | --- | --- | --- | --- | --- | --- | --- | --- | --- | --- |
| 82 | --- | --- | --- | --- | --- | --- | --- | --- | --- | --- |
| 83 | --- | --- | --- | --- | --- | --- | --- | --- | --- | --- |
| 84 | --- | --- | --- | --- | --- | --- | --- | --- | --- | --- |
| 85 | --- | --- | --- | --- | --- | --- | --- | --- | --- | --- |
| 86 | --- | --- | --- | --- | --- | --- | --- | --- | --- | --- |
| 87 | 0.1816 | --- | --- | --- | --- | --- | --- | --- | --- | --- |
| 88 | --- | --- | --- | --- | --- | --- | --- | --- | --- | --- |
| 89 | -0.1949 | --- | --- | --- | --- | --- | --- | --- | --- | --- |
| 90 | --- | --- | --- | --- | --- | --- | --- | --- | --- | --- |
| 91 | --- | --- | --- | --- | --- | --- | --- | --- | --- | --- |
| 92 | --- | --- | --- | --- | --- | --- | --- | --- | --- | --- |
| 93 | --- | --- | --- | --- | --- | --- | --- | --- | --- | --- |
| 94 | --- | --- | --- | --- | --- | --- | --- | --- | --- | --- |
| 95 | 0.1844 | -0.1342 | --- | --- | --- | --- | --- | --- | --- | --- |
| 96 | -0.1728 | -0.2199 | --- | --- | --- | --- | --- | --- | --- | --- |
| 97 | --- | --- | --- | --- | --- | --- | --- | --- | --- | --- |
| 98 | --- | --- | --- | --- | --- | --- | --- | --- | --- | --- |
| 99 | --- | --- | --- | --- | --- | --- | --- | --- | --- | --- |
| 100 | --- | --- | --- | --- | --- | --- | --- | --- | --- | --- |
| 101 | --- | --- | --- | --- | --- | --- | --- | --- | --- | --- |
| 102 | -0.1404 | --- | --- | --- | --- | --- | --- | --- | --- | --- |
| 103 | --- | --- | --- | --- | --- | --- | --- | --- | --- | --- |
| 104 | --- | --- | --- | --- | --- | --- | --- | --- | --- | --- |
| 105 | 0.2099 | --- | --- | --- | --- | --- | --- | --- | --- | --- |
| 106 | --- | --- | --- | --- | --- | --- | --- | --- | --- | --- |
| 107 | --- | --- | --- | --- | --- | --- | --- | --- | --- | --- |
| 108 | --- | --- | --- | --- | --- | --- | --- | --- | --- | --- |
| 109 | --- | 0.2210 | --- | --- | --- | --- | 0.1384 | 0.1610 | --- | --- |
| 110 | --- | --- | --- | --- | --- | --- | --- | --- | --- | --- |
| 111 | --- | --- | --- | --- | --- | --- | --- | --- | --- | --- |
| 112 | --- | --- | --- | --- | --- | --- | --- | --- | --- | --- |
| 113 | --- | --- | --- | --- | --- | --- | --- | --- | --- | --- |
| 114 | --- | --- | --- | --- | --- | --- | --- | --- | --- | --- |
| 115 | --- | --- | --- | --- | --- | --- | --- | --- | --- | --- |
| 116 | --- | --- | --- | --- | --- | --- | --- | --- | --- | --- |
| 117 | --- | -0.1752 | --- | --- | --- | --- | --- | --- | --- | --- |
| 118 | --- | --- | --- | --- | --- | --- | --- | --- | --- | --- |
| 119 | --- | --- | --- | --- | --- | --- | 0.1469 | --- | --- | --- |
| 120 | --- | 0.1404 | --- | --- | --- | --- | --- | --- | --- | --- |
| 121 | --- | -0.1295 | --- | --- | --- | --- | --- | --- | --- | --- |
| 122 | --- | --- | --- | --- | --- | --- | --- | --- | --- | --- |
| 123 | --- | --- | --- | --- | --- | --- | --- | --- | --- | --- |
| 124 | --- | --- | --- | --- | --- | --- | --- | --- | --- | --- |
| 125 | --- | --- | --- | --- | --- | --- | --- | --- | --- | --- |

Supplementary Table 3. Path-weights corresponding to the connections that take to output O, which survived pruning. Inputs are in rows. Darker red means higher path-weights values.

| **IC** | **Hidden nodes** | | | | | | | | | |
| --- | --- | --- | --- | --- | --- | --- | --- | --- | --- | --- |
|  | **1** | **2** | **3** | **4** | **5** | **6** | **7** | **8** | **9** | **10** |
| 1 | 0.2616 | --- | --- | --- | --- | --- | --- | --- | --- | 1.0378 |
| 2 | 0.4052 | --- | --- | -0.2500 | --- | --- | --- | --- | --- | 1.1267 |
| 3 | -0.2486 | --- | --- | 0.6639 | --- | --- | --- | --- | --- | --- |
| 4 | --- | --- | -0.1378 | -0.7842 | --- | --- | 0.1987 | --- | --- | -0.2228 |
| 5 | --- | --- | --- | 0.2292 | --- | --- | 0.1971 | --- | --- | 1.0269 |
| 6 | --- | --- | --- | --- | --- | --- | --- | --- | --- | -0.7722 |
| 7 | --- | --- | --- | -0.2308 | --- | --- | --- | -0.3462 | --- | -0.3681 |
| 8 | --- | --- | --- | --- | --- | --- | --- | --- | --- | 0.3663 |
| 9 | -0.2683 | --- | --- | 0.2269 | --- | --- | -0.1737 | --- | --- | -0.9898 |
| 10 | --- | --- | --- | -0.2199 | --- | --- | --- | --- | --- | -0.1289 |
| 11 | --- | --- | --- | --- | --- | --- | --- | -0.1514 | --- | -0.2722 |
| 12 | --- | --- | --- | 0.3582 | --- | --- | --- | --- | --- | 1.0931 |
| 13 | --- | -0.1192 | --- | --- | --- | --- | --- | --- | --- | -0.2708 |
| 14 | -0.5359 | --- | --- | 0.8798 | --- | --- | -0.1586 | --- | --- | -0.5456 |
| 15 | 0.1878 | -0.1563 | --- | -0.5948 | --- | --- | --- | --- | --- | -0.6614 |
| 16 | --- | --- | --- | --- | --- | --- | --- | -0.2485 | --- | -0.4011 |
| 17 | --- | --- | --- | --- | --- | --- | --- | --- | --- | --- |
| 18 | --- | --- | --- | -0.4877 | --- | --- | --- | --- | --- | -0.3014 |
| 19 | --- | -0.1144 | --- | -0.4413 | --- | --- | --- | -0.2348 | --- | -0.2764 |
| 20 | --- | --- | --- | --- | --- | --- | --- | --- | --- | 0.3082 |
| 21 | --- | --- | --- | --- | --- | --- | --- | -0.1206 | --- | --- |
| 22 | --- | --- | --- | -0.2871 | --- | --- | --- | --- | --- | --- |
| 23 | --- | --- | --- | 0.2026 | --- | --- | --- | --- | --- | --- |
| 24 | --- | --- | --- | --- | --- | --- | --- | --- | --- | --- |
| 25 | --- | --- | --- | --- | --- | --- | --- | --- | --- | --- |
| 26 | --- | --- | --- | 0.1983 | --- | --- | --- | --- | --- | --- |
| 27 | --- | --- | --- | 0.2700 | --- | --- | --- | --- | --- | -0.1667 |
| 28 | --- | --- | --- | --- | --- | --- | --- | --- | --- | -0.4526 |
| 29 | --- | --- | --- | -0.1969 | --- | --- | --- | --- | --- | -0.1481 |
| 30 | --- | --- | --- | --- | --- | --- | --- | --- | --- | 0.2147 |
| 31 | --- | --- | --- | --- | --- | --- | --- | --- | --- | --- |
| 32 | -0.1696 | --- | --- | --- | --- | --- | --- | --- | --- | -0.3864 |
| 33 | --- | --- | --- | --- | --- | --- | --- | --- | --- | -0.2381 |
| 34 | -0.1810 | --- | --- | 0.2018 | --- | --- | --- | --- | --- | --- |
| 35 | --- | --- | --- | 0.2501 | --- | --- | --- | --- | --- | --- |
| 36 | --- | --- | --- | --- | --- | --- | --- | -0.1392 | --- | --- |
| 37 | --- | --- | --- | -0.1918 | --- | --- | --- | --- | --- | -0.3742 |
| 38 | --- | --- | --- | --- | --- | --- | --- | --- | --- | 0.3730 |
| 39 | --- | --- | --- | 0.2659 | --- | --- | --- | --- | --- | 0.2134 |
| 40 | --- | --- | --- | --- | --- | --- | --- | --- | --- | -0.1978 |
| 41 | --- | --- | --- | -0.1732 | --- | --- | --- | --- | --- | -0.1441 |
| 42 | --- | --- | --- | -0.2483 | --- | --- | --- | --- | --- | --- |
| 43 | --- | --- | --- | --- | --- | --- | --- | --- | --- | -0.2036 |
| 44 | --- | --- | --- | --- | --- | --- | --- | --- | --- | --- |
| 45 | --- | --- | --- | -0.1542 | --- | --- | --- | --- | --- | --- |
| 46 | --- | -0.1349 | --- | --- | --- | --- | --- | --- | --- | --- |
| 47 | --- | --- | --- | -0.1545 | --- | --- | --- | 0.1824 | --- | --- |
| 48 | --- | --- | --- | --- | --- | --- | --- | --- | --- | --- |
| 49 | --- | --- | --- | --- | --- | --- | --- | --- | --- | --- |
| 50 | --- | --- | --- | --- | --- | --- | --- | --- | --- | --- |
| 51 | --- | --- | --- | --- | --- | --- | --- | --- | --- | --- |
| 52 | --- | --- | --- | -0.2360 | --- | --- | --- | --- | --- | --- |
| 53 | --- | --- | --- | --- | --- | --- | --- | --- | --- | --- |
| 54 | --- | --- | --- | --- | --- | --- | --- | --- | --- | --- |
| 55 | --- | --- | --- | --- | --- | --- | --- | --- | --- | --- |
| 56 | -0.1473 | --- | --- | --- | --- | --- | --- | --- | --- | --- |
| 57 | --- | --- | --- | --- | --- | --- | --- | --- | --- | --- |
| 58 | --- | --- | --- | --- | --- | --- | --- | --- | --- | --- |
| 59 | --- | --- | --- | --- | --- | --- | --- | -0.1410 | --- | 0.4400 |
| 60 | --- | --- | --- | --- | --- | --- | --- | --- | --- | -0.1258 |
| 61 | --- | --- | --- | --- | --- | --- | --- | --- | --- | --- |
| 62 | --- | --- | --- | -0.1550 | --- | --- | --- | --- | --- | --- |
| 63 | --- | --- | --- | --- | --- | --- | --- | --- | --- | --- |
| 64 | --- | --- | --- | --- | --- | --- | --- | --- | --- | -0.4738 |
| 65 | --- | --- | --- | --- | --- | --- | --- | --- | --- | --- |

Supplementary Table 3. (cont.)

| **IC** | **Hidden nodes** | | | | | | | | | |
| --- | --- | --- | --- | --- | --- | --- | --- | --- | --- | --- |
|  | **1** | **2** | **3** | **4** | **5** | **6** | **7** | **8** | **9** | **10** |
| 66 | --- | --- | --- | -0.2422 | --- | --- | --- | --- | --- | 0.2226 |
| 67 | --- | --- | --- | --- | --- | --- | --- | --- | --- | -0.2275 |
| 68 | --- | --- | --- | --- | --- | --- | --- | --- | --- | --- |
| 69 | --- | --- | --- | --- | --- | --- | --- | --- | --- | --- |
| 70 | --- | --- | --- | 0.2147 | --- | --- | --- | --- | --- | --- |
| 71 | --- | --- | --- | --- | --- | --- | --- | --- | --- | --- |
| 72 | --- | --- | --- | --- | --- | --- | --- | --- | --- | --- |
| 73 | --- | --- | --- | --- | --- | --- | --- | --- | --- | --- |
| 74 | --- | --- | --- | -0.2170 | --- | --- | --- | --- | --- | 0.2201 |
| 75 | --- | --- | --- | --- | --- | --- | --- | --- | --- | --- |
| 76 | --- | --- | --- | --- | --- | --- | --- | --- | --- | -0.3732 |
| 77 | --- | --- | --- | --- | --- | --- | --- | --- | --- | --- |
| 78 | 0.2052 | --- | --- | -0.3679 | --- | --- | --- | --- | --- | --- |
| 79 | --- | --- | --- | -0.1611 | --- | --- | --- | --- | --- | 0.2879 |
| 80 | --- | --- | --- | --- | --- | --- | --- | --- | --- | --- |
| 81 | --- | --- | --- | --- | --- | --- | --- | --- | --- | -0.1266 |
| 82 | --- | --- | --- | --- | --- | --- | --- | --- | --- | -0.2390 |
| 83 | --- | --- | --- | --- | --- | --- | --- | --- | --- | -0.1373 |
| 84 | --- | --- | --- | --- | --- | --- | --- | --- | --- | --- |
| 85 | --- | --- | --- | --- | --- | --- | --- | --- | --- | --- |
| 86 | --- | --- | --- | --- | --- | --- | --- | --- | --- | --- |
| 87 | --- | --- | --- | --- | --- | --- | --- | --- | --- | --- |
| 88 | --- | --- | --- | --- | --- | --- | --- | --- | --- | 0.2381 |
| 89 | -0.1177 | --- | --- | --- | --- | --- | --- | --- | --- | --- |
| 90 | --- | --- | --- | -0.1345 | --- | --- | --- | --- | --- | --- |
| 91 | --- | --- | --- | --- | --- | --- | --- | --- | --- | --- |
| 92 | --- | --- | --- | --- | --- | --- | --- | --- | --- | 0.2995 |
| 93 | --- | --- | --- | --- | --- | --- | --- | --- | --- | 0.3785 |
| 94 | --- | --- | --- | -0.1463 | --- | --- | --- | --- | --- | --- |
| 95 | --- | --- | --- | --- | --- | --- | --- | --- | --- | --- |
| 96 | --- | --- | --- | --- | --- | --- | --- | --- | --- | 0.2199 |
| 97 | --- | --- | --- | --- | --- | --- | --- | --- | --- | --- |
| 98 | --- | --- | --- | --- | --- | --- | --- | --- | --- | 0.2553 |
| 99 | --- | --- | --- | --- | --- | --- | --- | --- | --- | -0.2181 |
| 100 | --- | --- | --- | --- | --- | --- | --- | --- | --- | --- |
| 101 | --- | --- | --- | --- | --- | --- | --- | --- | --- | --- |
| 102 | --- | --- | --- | --- | --- | --- | --- | --- | --- | --- |
| 103 | --- | --- | --- | --- | --- | --- | --- | --- | --- | -0.1592 |
| 104 | --- | --- | --- | --- | --- | --- | --- | --- | --- | --- |
| 105 | --- | --- | --- | -0.2446 | --- | --- | --- | --- | --- | 0.2058 |
| 106 | --- | --- | --- | --- | --- | --- | --- | --- | --- | --- |
| 107 | --- | --- | --- | --- | --- | --- | --- | --- | --- | --- |
| 108 | --- | --- | --- | -0.1211 | --- | --- | --- | --- | --- | 0.1921 |
| 109 | --- | --- | --- | -0.2151 | --- | --- | --- | --- | --- | 0.3301 |
| 110 | --- | --- | --- | --- | --- | --- | --- | --- | --- | --- |
| 111 | --- | --- | --- | --- | --- | --- | --- | --- | --- | -0.3401 |
| 112 | --- | --- | --- | --- | --- | --- | --- | --- | --- | -0.1811 |
| 113 | --- | --- | --- | --- | --- | --- | --- | --- | --- | --- |
| 114 | --- | --- | --- | --- | --- | --- | --- | --- | --- | --- |
| 115 | --- | --- | --- | --- | --- | --- | --- | --- | --- | --- |
| 116 | --- | --- | --- | --- | --- | --- | --- | --- | --- | --- |
| 117 | --- | --- | --- | --- | --- | --- | --- | --- | --- | -0.2194 |
| 118 | --- | --- | --- | --- | --- | --- | --- | --- | --- | --- |
| 119 | --- | --- | --- | 0.2788 | --- | --- | --- | --- | --- | -0.4034 |
| 120 | --- | --- | --- | --- | --- | --- | --- | --- | --- | -0.1735 |
| 121 | --- | --- | --- | --- | --- | --- | --- | --- | --- | -0.1787 |
| 122 | --- | --- | --- | --- | --- | --- | --- | --- | --- | --- |
| 123 | --- | --- | --- | --- | --- | --- | --- | --- | --- | --- |
| 124 | --- | --- | --- | --- | --- | --- | --- | --- | --- | --- |
| 125 | --- | --- | --- | --- | --- | --- | --- | --- | --- | --- |

Supplementary Table 4. Path-weights corresponding to the connections that take to output P, which survived pruning. Inputs are in rows. Darker red means higher path-weights values.

| **IC** | **Hidden nodes** | | | | | | | | | |
| --- | --- | --- | --- | --- | --- | --- | --- | --- | --- | --- |
|  | **1** | **2** | **3** | **4** | **5** | **6** | **7** | **8** | **9** | **10** |
| 1 | --- | --- | -0.3355 | --- | -0.2165 | --- | -0.2306 | --- | --- | -0.4058 |
| 2 | -0.1766 | --- | -0.3508 | -0.3133 | --- | --- | -0.2997 | --- | --- | -0.4405 |
| 3 | --- | --- | 0.4218 | 0.8321 | --- | --- | 0.1628 | --- | --- | --- |
| 4 | --- | --- | -0.4618 | -0.9829 | 0.1775 | --- | -0.3555 | --- | --- | --- |
| 5 | --- | --- | --- | 0.2872 | -0.3916 | --- | -0.3526 | --- | --- | -0.4015 |
| 6 | --- | --- | 0.1719 | --- | 0.2438 | --- | --- | --- | --- | 0.3019 |
| 7 | --- | --- | -0.2111 | -0.2892 | --- | -0.1863 | -0.148 | -0.2405 | --- | --- |
| 8 | --- | --- | --- | --- | --- | --- | --- | --- | --- | --- |
| 9 | --- | --- | 0.2946 | 0.2844 | 0.2373 | --- | 0.3107 | --- | --- | 0.387 |
| 10 | --- | --- | --- | -0.2756 | --- | --- | --- | --- | --- | --- |
| 11 | --- | --- | --- | --- | --- | --- | --- | --- | --- | --- |
| 12 | --- | --- | --- | 0.449 | -0.2995 | --- | -0.1814 | --- | --- | -0.4274 |
| 13 | --- | --- | --- | --- | 0.1989 | --- | --- | --- | --- | --- |
| 14 | 0.2336 | --- | 0.6025 | 1.1027 | --- | --- | 0.2837 | --- | --- | 0.2133 |
| 15 | --- | --- | -0.1681 | -0.7455 | --- | --- | 0.1879 | --- | --- | 0.2586 |
| 16 | --- | --- | --- | 0.1803 | 0.169 | --- | --- | -0.1726 | --- | --- |
| 17 | --- | --- | --- | --- | --- | --- | --- | --- | --- | --- |
| 18 | --- | --- | --- | -0.6113 | --- | --- | --- | --- | --- | --- |
| 19 | --- | --- | --- | -0.5531 | --- | --- | --- | -0.1631 | --- | --- |
| 20 | --- | --- | --- | --- | -0.1576 | --- | --- | --- | --- | --- |
| 21 | --- | --- | --- | --- | --- | --- | --- | --- | --- | --- |
| 22 | --- | --- | --- | -0.3598 | --- | --- | --- | --- | --- | --- |
| 23 | --- | --- | --- | 0.2539 | --- | --- | -0.2109 | --- | --- | --- |
| 24 | --- | --- | --- | --- | --- | --- | --- | --- | --- | --- |
| 25 | --- | --- | --- | --- | --- | --- | --- | --- | --- | --- |
| 26 | --- | --- | --- | 0.2485 | --- | --- | --- | --- | --- | --- |
| 27 | --- | --- | --- | 0.3384 | --- | --- | --- | --- | --- | --- |
| 28 | --- | --- | --- | --- | 0.1592 | --- | --- | --- | --- | 0.177 |
| 29 | --- | --- | --- | -0.2468 | --- | --- | --- | --- | --- | --- |
| 30 | --- | --- | --- | --- | --- | --- | --- | --- | --- | --- |
| 31 | --- | --- | --- | --- | --- | --- | --- | --- | --- | --- |
| 32 | --- | --- | 0.1879 | 0.2014 | --- | --- | --- | --- | --- | --- |
| 33 | --- | --- | 0.1899 | 0.2266 | --- | --- | 0.2038 | --- | --- | --- |
| 34 | --- | --- | 0.3199 | 0.253 | --- | --- | --- | --- | --- | --- |
| 35 | --- | --- | --- | 0.3135 | --- | --- | --- | --- | --- | --- |
| 36 | --- | --- | --- | --- | --- | --- | --- | --- | --- | --- |
| 37 | --- | --- | --- | -0.2404 | --- | --- | --- | --- | --- | --- |
| 38 | --- | --- | --- | --- | --- | --- | --- | --- | --- | --- |
| 39 | --- | --- | --- | 0.3333 | --- | --- | --- | --- | --- | --- |
| 40 | --- | --- | --- | --- | --- | --- | -0.2374 | --- | --- | --- |
| 41 | --- | --- | --- | -0.2171 | --- | --- | --- | --- | --- | --- |
| 42 | --- | --- | --- | -0.3112 | --- | --- | --- | --- | --- | --- |
| 43 | --- | --- | --- | --- | --- | --- | --- | --- | --- | --- |
| 44 | --- | --- | --- | --- | --- | --- | --- | --- | --- | --- |
| 45 | --- | --- | --- | -0.1932 | --- | --- | --- | --- | --- | --- |
| 46 | --- | --- | --- | --- | --- | --- | --- | --- | --- | --- |
| 47 | --- | --- | --- | -0.1937 | 0.1957 | --- | --- | --- | --- | --- |
| 48 | --- | --- | --- | --- | --- | --- | --- | --- | --- | --- |
| 49 | --- | --- | --- | --- | --- | --- | --- | --- | --- | --- |
| 50 | --- | --- | --- | --- | --- | --- | --- | --- | --- | --- |
| 51 | --- | --- | --- | --- | --- | --- | --- | --- | --- | --- |
| 52 | --- | --- | --- | -0.2958 | --- | --- | --- | --- | --- | --- |
| 53 | --- | --- | --- | --- | --- | --- | --- | --- | --- | --- |
| 54 | --- | --- | --- | 0.2074 | --- | --- | --- | --- | --- | --- |
| 55 | --- | --- | --- | --- | --- | --- | --- | --- | --- | --- |
| 56 | --- | --- | 0.2363 | 0.2084 | --- | --- | --- | --- | --- | --- |
| 57 | --- | --- | --- | --- | --- | --- | --- | --- | --- | --- |
| 58 | --- | --- | --- | --- | --- | --- | --- | --- | --- | --- |
| 59 | --- | --- | --- | --- | --- | --- | --- | --- | --- | -0.172 |
| 60 | --- | --- | -0.1747 | --- | --- | --- | --- | --- | --- | --- |
| 61 | --- | --- | --- | --- | --- | --- | --- | --- | --- | --- |
| 62 | --- | --- | --- | -0.1943 | --- | --- | --- | --- | --- | --- |
| 63 | --- | --- | --- | --- | --- | --- | --- | --- | --- | --- |
| 64 | --- | --- | --- | --- | --- | --- | --- | --- | --- | 0.1852 |
| 65 | --- | --- | --- | --- | --- | --- | --- | --- | --- | --- |

Supplementary Table 4. (cont.)

| **IC** | **Hidden nodes** | | | | | | | | | |
| --- | --- | --- | --- | --- | --- | --- | --- | --- | --- | --- |
|  | **1** | **2** | **3** | **4** | **5** | **6** | **7** | **8** | **9** | **10** |
| 66 | --- | --- | --- | -0.3035 | --- | --- | --- | --- | --- | --- |
| 67 | --- | --- | --- | --- | --- | --- | --- | --- | --- | --- |
| 68 | --- | --- | --- | --- | --- | --- | --- | --- | --- | --- |
| 69 | --- | --- | --- | 0.2038 | --- | --- | --- | --- | --- | --- |
| 70 | --- | --- | --- | 0.269 | --- | --- | --- | --- | --- | --- |
| 71 | --- | --- | --- | --- | --- | --- | --- | --- | --- | --- |
| 72 | --- | --- | --- | --- | --- | --- | --- | --- | --- | --- |
| 73 | --- | --- | --- | --- | --- | --- | --- | --- | --- | --- |
| 74 | --- | --- | --- | -0.272 | --- | --- | --- | --- | --- | --- |
| 75 | --- | --- | --- | --- | --- | --- | --- | --- | --- | --- |
| 76 | --- | --- | --- | --- | --- | --- | --- | --- | --- | --- |
| 77 | --- | --- | --- | --- | --- | --- | --- | --- | --- | --- |
| 78 | --- | --- | --- | -0.4611 | --- | --- | --- | --- | --- | --- |
| 79 | --- | --- | --- | -0.202 | --- | --- | --- | --- | --- | --- |
| 80 | --- | --- | --- | --- | --- | --- | -0.2141 | --- | --- | --- |
| 81 | --- | --- | --- | --- | --- | --- | --- | --- | --- | --- |
| 82 | --- | --- | --- | --- | --- | --- | --- | --- | --- | --- |
| 83 | --- | --- | --- | --- | --- | --- | --- | --- | --- | --- |
| 84 | --- | --- | --- | --- | --- | --- | --- | --- | --- | --- |
| 85 | --- | --- | --- | 0.2228 | --- | --- | --- | --- | --- | --- |
| 86 | --- | --- | --- | --- | --- | --- | --- | --- | --- | --- |
| 87 | --- | --- | --- | --- | --- | --- | --- | --- | --- | --- |
| 88 | --- | --- | --- | --- | --- | --- | --- | --- | --- | --- |
| 89 | --- | --- | --- | --- | --- | --- | --- | --- | --- | --- |
| 90 | --- | --- | --- | -0.1686 | --- | --- | --- | --- | --- | --- |
| 91 | --- | --- | --- | --- | --- | --- | --- | --- | --- | --- |
| 92 | --- | --- | --- | --- | --- | --- | --- | --- | --- | --- |
| 93 | --- | --- | --- | --- | --- | --- | --- | --- | --- | --- |
| 94 | --- | --- | --- | -0.1833 | --- | --- | --- | --- | --- | --- |
| 95 | --- | --- | --- | --- | --- | --- | --- | --- | --- | --- |
| 96 | --- | --- | --- | 0.2013 | --- | --- | --- | --- | --- | --- |
| 97 | --- | --- | --- | --- | --- | --- | --- | --- | --- | --- |
| 98 | --- | --- | 0.1797 | 0.1856 | --- | --- | --- | --- | --- | --- |
| 99 | --- | --- | --- | 0.1851 | --- | --- | --- | --- | --- | --- |
| 100 | --- | --- | -0.1686 | --- | --- | --- | --- | --- | --- | --- |
| 101 | --- | --- | --- | --- | --- | --- | --- | --- | --- | --- |
| 102 | --- | --- | --- | --- | --- | --- | --- | --- | --- | --- |
| 103 | --- | --- | --- | --- | --- | --- | --- | --- | --- | --- |
| 104 | --- | --- | --- | --- | --- | --- | --- | --- | --- | --- |
| 105 | --- | --- | --- | -0.3065 | --- | --- | --- | --- | --- | --- |
| 106 | --- | --- | --- | --- | --- | --- | --- | --- | --- | --- |
| 107 | --- | --- | --- | --- | --- | --- | --- | --- | --- | --- |
| 108 | --- | --- | --- | -0.1518 | --- | --- | --- | --- | --- | --- |
| 109 | --- | --- | --- | -0.2696 | --- | --- | 0.1912 | --- | --- | --- |
| 110 | --- | --- | --- | --- | --- | --- | --- | --- | --- | --- |
| 111 | --- | --- | --- | --- | --- | --- | --- | --- | --- | --- |
| 112 | --- | --- | --- | --- | --- | --- | --- | --- | --- | --- |
| 113 | --- | --- | --- | --- | --- | --- | --- | --- | --- | --- |
| 114 | --- | --- | --- | --- | --- | --- | --- | --- | --- | --- |
| 115 | --- | --- | --- | 0.1646 | --- | --- | --- | --- | --- | --- |
| 116 | --- | --- | --- | --- | --- | --- | --- | --- | --- | --- |
| 117 | --- | --- | --- | --- | --- | --- | --- | --- | --- | --- |
| 118 | --- | --- | --- | --- | --- | --- | --- | --- | --- | --- |
| 119 | --- | --- | --- | 0.3494 | --- | --- | 0.203 | --- | --- | --- |
| 120 | --- | --- | --- | --- | --- | --- | --- | --- | --- | --- |
| 121 | --- | --- | --- | --- | --- | --- | --- | --- | --- | --- |
| 122 | --- | --- | --- | --- | --- | --- | --- | --- | --- | --- |
| 123 | --- | --- | --- | --- | --- | --- | --- | --- | --- | --- |
| 124 | --- | --- | --- | 0.2061 | --- | --- | --- | --- | --- | --- |
| 125 | --- | --- | --- | --- | --- | --- | --- | --- | --- | --- |

Supplementary Table 5 contains the values of the weights that survived pruning for the connections between the input layer and the hidden layer. For example, the weight IC1 (input) → Hidden node 2 is pruned, and, therefore, no magnitude is presented. The table cells are color-coded. Darker red cells mean higher weights, i.e., connections that favor the information fed in the respective input and, therefore, contribute more to the final decision (model predictions).

Supplementary Table 5. Weights corresponding to the connections between the input layer and the hidden layer of the pruned network. Inputs are in rows. Darker red means higher positive weights, and darker blue means higher negative weights. The symbol “---” means the weight was pruned.

| **IC** | **Hidden nodes** | | | | | | | | | |
| --- | --- | --- | --- | --- | --- | --- | --- | --- | --- | --- |
|  | **1** | **2** | **3** | **4** | **5** | **6** | **7** | **8** | **9** | **10** |
| Bias | -0.2139 | 0.0723 | -0.1866 | 0.2882 | -0.2597 | 0.1963 | -0.0465 | 0.3321 | --- | 0.1736 |
| 1 | 0.3214 | --- | -0.2687 | --- | -0.2748 | --- | -0.2291 | --- | --- | -0.5725 |
| 2 | 0.4897 | --- | -0.2830 | 0.2005 | --- | --- | -0.2752 | --- | --- | -0.6035 |
| 3 | -0.3020 | 0.2027 | 0.2944 | -0.5030 | -0.1690 | --- | 0.2074 | --- | --- | --- |
| 4 | 0.2169 | --- | -0.3701 | 0.6115 | 0.1600 | --- | -0.3291 | --- | --- | 0.1521 |
| 5 | 0.0921 | 0.2409 | --- | -0.1523 | -0.4740 | --- | -0.3395 | --- | --- | -0.5733 |
| 6 | --- | -0.1671 | 0.1245 | --- | 0.2409 | --- | --- | -0.1336 | --- | 0.4057 |
| 7 | --- | --- | -0.1428 | 0.1435 | --- | 0.1855 | -0.1518 | 0.3957 | --- | 0.1942 |
| 8 | 0.2251 | --- | --- | --- | --- | --- | -0.1421 | --- | --- | -0.2157 |
| 9 | -0.3232 | --- | 0.1812 | -0.1377 | 0.1913 | --- | 0.4538 | --- | --- | 0.5122 |
| 10 | --- | --- | --- | 0.2392 | --- | --- | --- | -0.1314 | --- | 0.0185 |
| 11 | --- | --- | --- | --- | --- | --- | --- | 0.1364 | --- | 0.1330 |
| 12 | --- | 0.1911 | --- | -0.2582 | -0.3735 | --- | -0.1099 | --- | --- | -0.6197 |
| 13 | --- | -0.2745 | --- | --- | 0.1764 | --- | --- | --- | --- | 0.1634 |
| 14 | -0.6754 | 0.3146 | 0.4085 | -0.6391 | --- | --- | 0.3589 | --- | --- | 0.2516 |
| 15 | 0.2624 | -0.3026 | -0.1183 | 0.4355 | 0.1301 | --- | 0.2238 | --- | --- | 0.3386 |
| 16 | --- | --- | --- | -0.1446 | 0.2375 | --- | --- | 0.2477 | --- | 0.2091 |
| 17 | --- | --- | --- | --- | -0.1986 | --- | --- | --- | --- | --- |
| 18 | --- | -0.3019 | --- | 0.3121 | 0.1094 | --- | --- | --- | --- | 0.0954 |
| 19 | 0.1835 | -0.2846 | --- | 0.3227 | --- | --- | --- | 0.3170 | --- | 0.1741 |
| 20 | --- | --- | --- | --- | -0.1541 | --- | --- | -0.1126 | --- | -0.2044 |
| 21 | --- | --- | --- | --- | 0.0990 | --- | --- | 0.1433 | --- | --- |
| 22 | --- | --- | --- | 0.1897 | --- | --- | --- | -0.1218 | --- | --- |
| 23 | --- | 0.1655 | --- | -0.1771 | --- | --- | -0.2389 | --- | --- | --- |
| 24 | --- | --- | --- | --- | --- | --- | --- | -0.1532 | --- | --- |
| 25 | --- | --- | --- | --- | --- | --- | --- | -0.0998 | --- | --- |
| 26 | --- | --- | --- | -0.1149 | --- | --- | --- | --- | --- | --- |
| 27 | -0.1484 | --- | --- | -0.1854 | 0.0113 | --- | --- | -0.1130 | --- | 0.0865 |
| 28 | --- | --- | --- | --- | 0.0784 | --- | --- | --- | --- | 0.2630 |
| 29 | --- | --- | --- | 0.1356 | 0.0894 | --- | --- | -0.1116 | --- | 0.0441 |
| 30 | --- | 0.0237 | --- | --- | --- | --- | --- | --- | --- | -0.1298 |
| 31 | --- | --- | --- | --- | --- | --- | --- | -0.1194 | --- | --- |
| 32 | -0.2080 | 0.1116 | 0.1310 | -0.1238 | --- | --- | --- | --- | --- | 0.1784 |
| 33 | --- | --- | 0.0975 | -0.1118 | --- | --- | 0.3127 | --- | --- | 0.1112 |
| 34 | -0.2203 | --- | 0.2736 | -0.1688 | --- | --- | --- | --- | --- | --- |
| 35 | --- | 0.1835 | --- | -0.1949 | --- | --- | --- | --- | --- | --- |
| 36 | --- | 0.1048 | --- | --- | --- | --- | --- | 0.0867 | --- | --- |
| 37 | --- | --- | --- | 0.1971 | 0.1428 | --- | -0.0944 | --- | --- | 0.1918 |
| 38 | --- | --- | --- | --- | -0.1018 | --- | --- | --- | --- | -0.2397 |
| 39 | --- | 0.1378 | --- | -0.1426 | --- | --- | --- | --- | --- | -0.1185 |
| 40 | --- | 0.1597 | --- | --- | 0.0783 | --- | -0.1523 | --- | --- | 0.0427 |
| 41 | --- | --- | --- | 0.0232 | 0.1190 | --- | --- | --- | --- | 0.0796 |
| 42 | --- | --- | --- | 0.1742 | --- | --- | --- | -0.1321 | --- | --- |
| 43 | -0.0979 | --- | --- | --- | --- | --- | --- | -0.0696 | --- | 0.0425 |
| 44 | --- | --- | --- | --- | --- | --- | --- | --- | --- | --- |
| 45 | --- | --- | --- | 0.1462 | --- | --- | --- | --- | --- | --- |
| 46 | --- | -0.2512 | --- | --- | 0.0098 | --- | -0.1149 | --- | --- | --- |
| 47 | --- | --- | --- | 0.1661 | 0.1467 | --- | --- | -0.1763 | --- | --- |
| 48 | -0.1020 | --- | --- | --- | --- | --- | --- | -0.0620 | --- | --- |
| 49 | --- | --- | --- | --- | --- | --- | --- | --- | --- | --- |
| 50 | --- | 0.1221 | --- | --- | --- | --- | --- | --- | --- | --- |
| 51 | --- | --- | --- | --- | --- | --- | --- | --- | --- | --- |
| 52 | --- | --- | --- | 0.1084 | --- | --- | --- | --- | --- | --- |
| 53 | --- | --- | --- | --- | --- | --- | --- | --- | --- | --- |
| 54 | -0.0458 | --- | --- | -0.0450 | --- | --- | --- | --- | --- | --- |

**Supplementary Table 5.** (cont.)

| **IC** | **Hidden nodes** | | | | | | | | | |
| --- | --- | --- | --- | --- | --- | --- | --- | --- | --- | --- |
|  | **1** | **2** | **3** | **4** | **5** | **6** | **7** | **8** | **9** | **10** |
| 55 | --- | --- | --- | --- | --- | --- | --- | --- | --- | --- |
| 56 | -0.1856 | --- | 0.1899 | -0.1210 | --- | --- | --- | --- | --- | --- |
| 57 | 0.1372 | -0.2214 | --- | --- | --- | --- | --- | --- | --- | --- |
| 58 | --- | 0.1325 | --- | --- | --- | --- | --- | -0.0698 | --- | --- |
| 59 | 0.1775 | --- | --- | --- | -0.0891 | --- | --- | 0.1667 | --- | -0.2397 |
| 60 | --- | --- | -0.2069 | --- | 0.0178 | --- | --- | --- | --- | 0.0536 |
| 61 | --- | -0.2154 | --- | --- | --- | --- | --- | --- | --- | --- |
| 62 | --- | --- | --- | 0.1182 | --- | --- | 0.1907 | -0.0809 | --- | --- |
| 63 | --- | --- | --- | --- | --- | --- | --- | --- | --- | --- |
| 64 | -0.0705 | --- | --- | --- | 0.0855 | --- | --- | --- | --- | 0.2189 |
| 65 | --- | --- | --- | --- | --- | --- | --- | --- | --- | --- |
| 66 | 0.0986 | 0.1117 | --- | 0.1849 | --- | --- | -0.0374 | --- | --- | -0.1037 |
| 67 | --- | --- | --- | --- | 0.0619 | --- | --- | 0.1521 | --- | 0.1198 |
| 68 | --- | 0.1589 | --- | --- | --- | --- | -0.0447 | --- | --- | --- |
| 69 | --- | --- | --- | -0.0924 | --- | --- | --- | --- | --- | --- |
| 70 | --- | --- | --- | -0.1147 | --- | --- | --- | --- | --- | --- |
| 71 | --- | --- | --- | --- | --- | --- | --- | --- | --- | --- |
| 72 | --- | --- | --- | --- | --- | --- | --- | --- | --- | --- |
| 73 | --- | --- | --- | --- | --- | --- | --- | --- | --- | --- |
| 74 | --- | --- | --- | 0.1891 | --- | --- | --- | --- | --- | -0.1767 |
| 75 | 0.0763 | --- | --- | --- | --- | --- | --- | --- | --- | --- |
| 76 | -0.0715 | --- | --- | --- | 0.0515 | --- | --- | 0.1251 | --- | 0.1655 |
| 77 | --- | --- | --- | --- | --- | --- | --- | --- | --- | --- |
| 78 | 0.2945 | -0.2578 | --- | 0.2736 | --- | --- | --- | --- | --- | --- |
| 79 | --- | 0.1191 | --- | 0.1410 | -0.0746 | --- | --- | --- | --- | -0.1953 |
| 80 | --- | --- | --- | --- | --- | --- | -0.1403 | 0.1562 | --- | --- |
| 81 | --- | --- | --- | --- | --- | --- | --- | --- | --- | 0.0403 |
| 82 | --- | --- | --- | --- | --- | --- | --- | --- | --- | 0.1027 |
| 83 | --- | --- | --- | --- | --- | --- | --- | --- | --- | 0.0825 |
| 84 | --- | --- | --- | --- | --- | --- | --- | --- | --- | --- |
| 85 | --- | --- | --- | -0.0974 | --- | --- | --- | -0.0188 | --- | --- |
| 86 | --- | --- | --- | --- | --- | --- | --- | --- | --- | --- |
| 87 | 0.1893 | --- | --- | --- | --- | --- | --- | 0.1472 | --- | --- |
| 88 | --- | --- | --- | --- | --- | --- | --- | --- | --- | -0.1214 |
| 89 | -0.1578 | --- | --- | --- | --- | --- | -0.0746 | -0.0841 | --- | --- |
| 90 | --- | --- | --- | 0.0380 | --- | --- | --- | --- | --- | --- |
| 91 | --- | --- | --- | --- | --- | --- | --- | --- | --- | --- |
| 92 | --- | --- | --- | --- | --- | --- | --- | --- | --- | -0.1471 |
| 93 | --- | --- | --- | --- | --- | --- | --- | -0.0570 | --- | -0.2335 |
| 94 | --- | --- | --- | 0.1408 | 0.0292 | --- | --- | --- | --- | --- |
| 95 | 0.1322 | 0.1180 | --- | --- | --- | --- | --- | 0.0031 | --- | --- |
| 96 | -0.0957 | 0.1664 | --- | -0.1314 | -0.1100 | --- | --- | -0.1138 | --- | -0.1444 |
| 97 | --- | --- | --- | --- | --- | --- | --- | --- | --- | --- |
| 98 | --- | --- | 0.1239 | -0.1052 | --- | --- | --- | --- | --- | -0.1668 |
| 99 | --- | --- | --- | -0.1124 | --- | --- | --- | --- | --- | 0.1340 |
| 100 | --- | --- | -0.1571 | --- | --- | --- | --- | --- | --- | --- |
| 101 | --- | --- | --- | --- | --- | --- | --- | --- | --- | --- |
| 102 | -0.0232 | --- | --- | --- | --- | --- | --- | --- | --- | --- |
| 103 | --- | --- | --- | --- | --- | --- | -0.0322 | --- | --- | 0.0082 |
| 104 | --- | --- | --- | --- | --- | --- | --- | --- | --- | --- |
| 105 | 0.1109 | --- | --- | 0.0715 | --- | --- | --- | --- | --- | -0.1236 |
| 106 | --- | --- | --- | --- | --- | --- | --- | --- | --- | --- |
| 107 | --- | --- | --- | --- | --- | --- | --- | --- | --- | --- |
| 108 | --- | --- | --- | 0.0857 | --- | --- | --- | --- | --- | -0.0997 |
| 109 | --- | -0.2240 | --- | 0.1792 | --- | --- | 0.2472 | -0.1355 | --- | -0.1893 |
| 110 | --- | --- | --- | --- | --- | --- | --- | --- | --- | --- |
| 111 | --- | --- | --- | --- | --- | --- | --- | --- | --- | 0.1660 |
| 112 | --- | --- | --- | --- | --- | --- | --- | -0.0489 | --- | 0.0627 |
| 113 | --- | --- | --- | --- | --- | --- | --- | --- | --- | --- |
| 114 | --- | --- | --- | --- | --- | --- | --- | --- | --- | --- |
| 115 | --- | --- | --- | -0.1416 | --- | --- | --- | 0.1366 | --- | --- |
| 116 | --- | --- | --- | --- | --- | --- | --- | --- | --- | --- |
| 117 | --- | 0.1551 | --- | --- | --- | --- | --- | --- | --- | 0.0713 |
| 118 | --- | --- | --- | --- | -0.0990 | --- | --- | --- | --- | --- |
| 119 | --- | --- | --- | -0.2263 | --- | --- | 0.3351 | --- | --- | 0.2438 |

**Supplementary Table 5.** (cont.)

| **IC** | **Hidden nodes** | | | | | | | | | |
| --- | --- | --- | --- | --- | --- | --- | --- | --- | --- | --- |
|  | **1** | **2** | **3** | **4** | **5** | **6** | **7** | **8** | **9** | **10** |
| 120 | --- | -0.1573 | --- | --- | --- | --- | --- | --- | --- | 0.0587 |
| 121 | --- | 0.0567 | --- | --- | --- | --- | --- | --- | --- | 0.0503 |
| 122 | --- | --- | --- | --- | --- | --- | --- | --- | --- | --- |
| 123 | --- | --- | --- | --- | --- | --- | --- | -0.0962 | --- | --- |
| 124 | --- | --- | --- | -0.1201 | --- | --- | --- | --- | --- | --- |
| 125 | --- | --- | --- | --- | --- | --- | --- | --- | --- | --- |

Supplementary Table 6 contains the values of the weights that survived pruning for the connections between the hidden layer and the output layer. For example, the weight Hidden node 2 → BP (output) is pruned, and, therefore, no magnitude is presented. The table cells are color-coded. Darker red cells mean higher weights, i.e., connections that favor the information fed in the respective node and, therefore, contribute more to the final decision (model predictions).

Supplementary Table 6. Weights corresponding to the connections between the hidden layer and the output layer of the pruned network. Darker red means higher positive weights, and darker blue means higher negative weights. The symbol “---” means the weight was pruned.

| **Hidden nodes** | **Outputs** | | | |
| --- | --- | --- | --- | --- |
|  | **BP** | **BI** | **O** | **P** |
| Bias | -0.1775 | -0.1339 | -0.2007 | -0.1204 |
| 1 | 0.8298 | 1.3821 | 0.8229 | -0.3506 |
| 2 | --- | -1.0079 | 0.5608 | --- |
| 3 | --- | --- | 0.4327 | 1.4130 |
| 4 | --- | --- | -1.4458 | -1.7186 |
| 5 | 1.2950 | 0.8543 | --- | 1.0185 |
| 6 | -0.5812 | --- | --- | -0.9403 |
| 7 | -0.6587 | 0.6380 | -0.5136 | 0.9164 |
| 8 | -1.1889 | -0.9859 | -0.9161 | -0.5881 |
| 9 | --- | --- | --- | --- |
| 10 | 0.3727 | -0.3633 | -1.8996 | 0.7708 |

# SHAP values

Supplementary Figure 1 and Supplementary Figure 2 depict the full lists of the SHAP values for BP, BI, O, and P in the retrained network. The ICs (features) are ranked by their importance. More important ICs for the model’s final prediction are plotted on the top.

Supplementary Figure 3 depicts a bar plot containing the absolute SHAP values for the retrained network's four categories (BP, BI, O, and P). The list is ordered from the higher SHAP values to the least. ICs missing a SHAP value means they were fully pruned, i.e., no connection subsists to the hidden nodes and outputs. Supplementary Figure 4 depicts a bar plot containing the absolute SHAP values for the hidden nodes in the retrained network. As previously, the list is ordered from the higher SHAP values to the least. Hidden nodes missing a SHAP value means they were pruned.


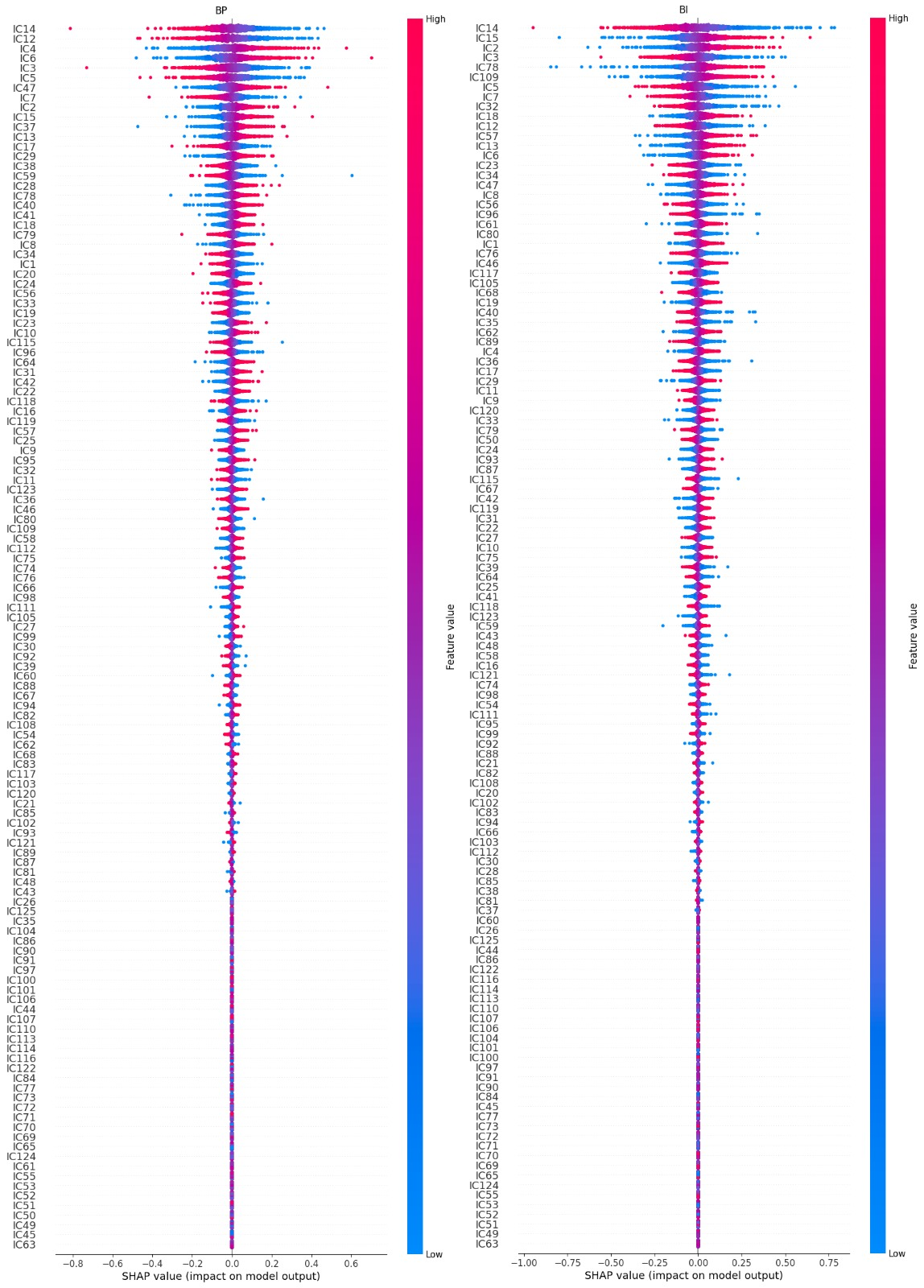


Supplementary Figure 2. Complete list of the SHAP values of the retrained network for BP (left) and BI (right). Higher rank means more influence in the output computation either positively (red on the right), or negatively (blue on the right). For example, IC4 and IC6 are the inputs that most positively contribute to the BP output selection, whereas IC15 and IC2 are the inputs that most contribute to BI.


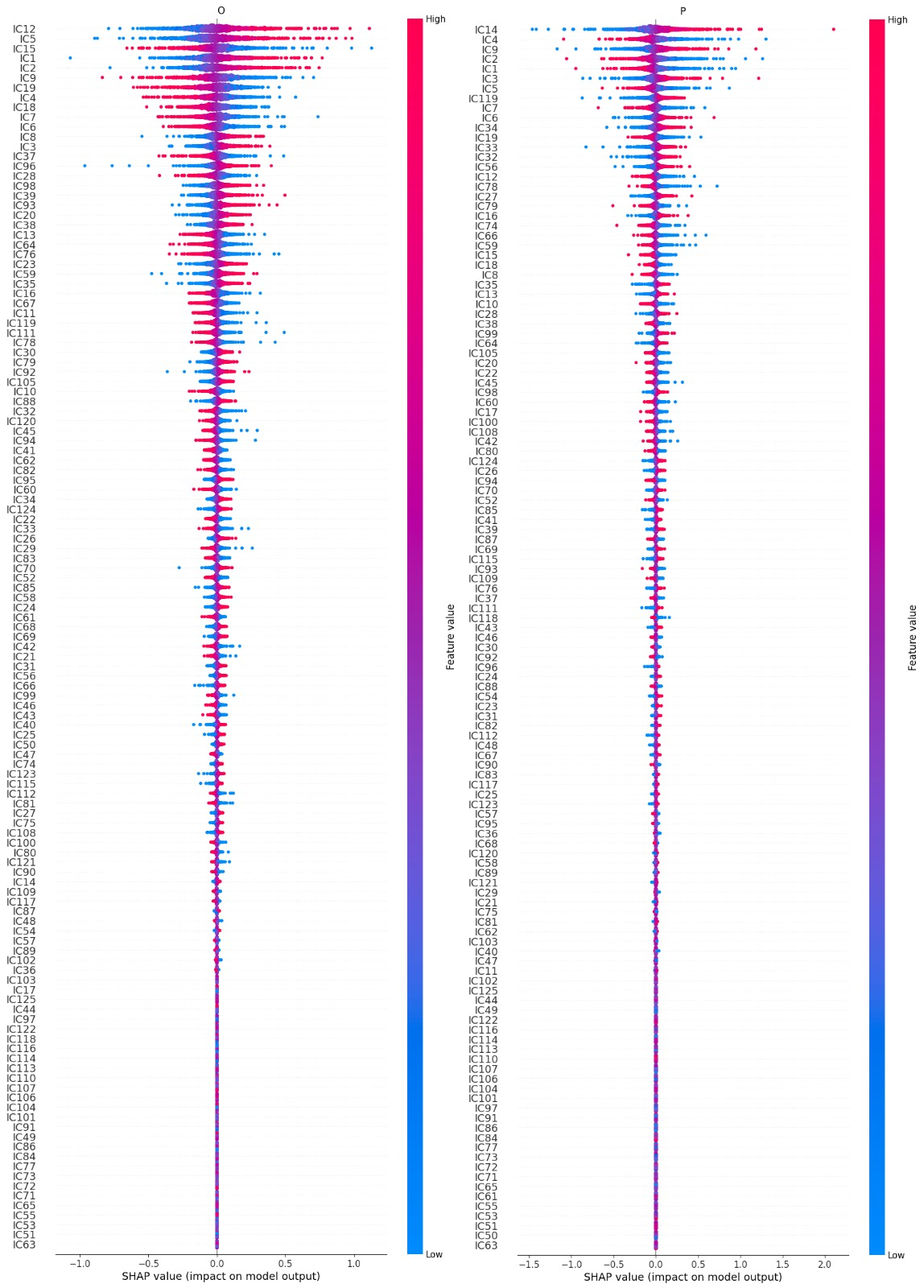


Supplementary Figure 3. Complete list of the SHAP values of the retrained network for O (left) and P (right). Higher rank means more influence in the output computation either positively (red on the right), or negatively (blue on the right). For example, IC12 and IC5 are the inputs that most positively contribute to the O output selection, whereas IC14 and IC9 are the inputs that most contribute to P.


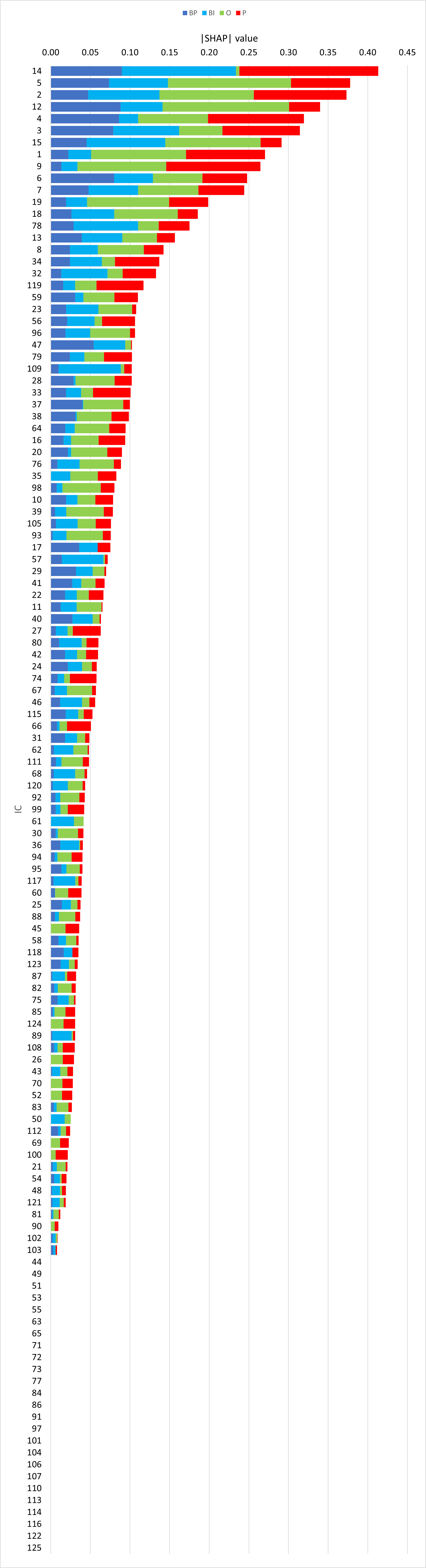


Supplementary Figure 4. Bar plot with the absolute SHAP values of all ICs in the retrained network. The width of each colored segment is proportional to the SHAP value, i.e., the wider it is, the more important it is for classification, contributing either positively or negatively.


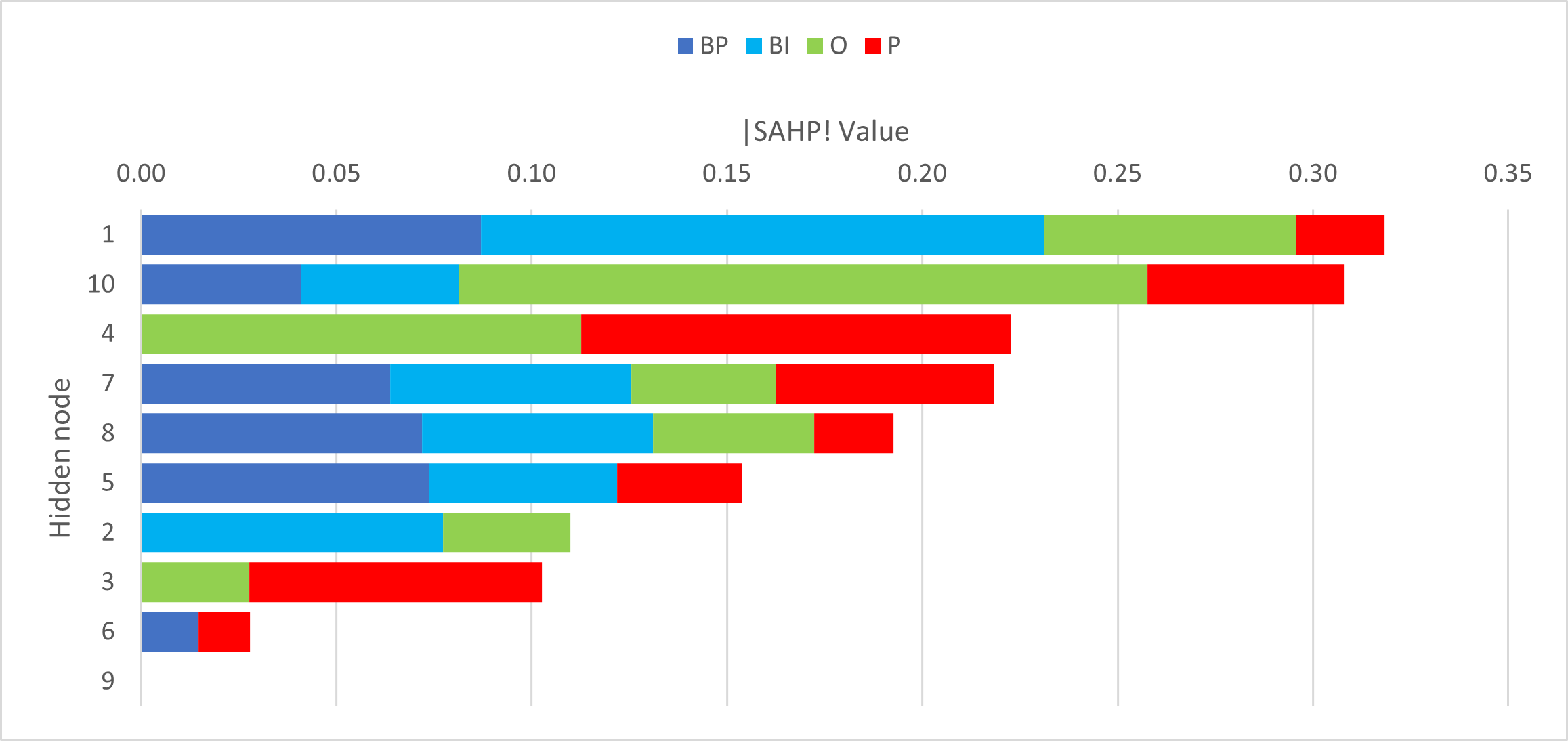


Supplementary Figure 5. Bar plot with the absolute SHAP values of all hidden nodes in the retrained network. The width of each colored segment is proportional to the SHAP value, i.e., the wider it is, the more important it is for classification, contributing either positively or negatively.

# IC’s Spatial Maps

Supplementary Figure 5 depicts extra images of the ICs that participate in the brand classification, i.e., ICs that are important both for BP and BI classification, which are identified in Table 2.

Supplementary Figure 6 and Supplementary Figure 7 depict extra images of the ICs that participate in brand|preferred classification, i.e., ICs that are important for BP classification, which are identified in Table 3.


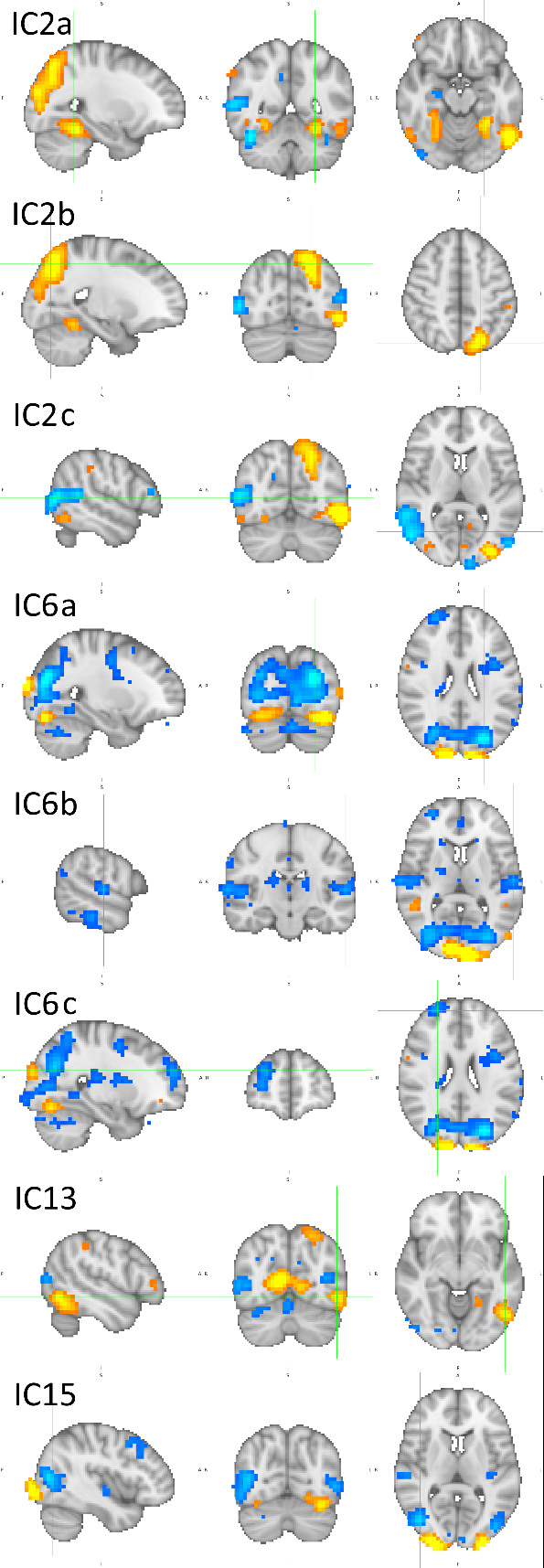


Supplementary Figure 6. Sagittal, coronal, and axial views of IC2a (x=-26, y=-50, z=-16), IC2b (x=-22, y=-74, z=48), IC2c (x=54, y=-66, z=8), IC6a (x=-26, y=-78, z=24), IC6b (x=-58, y=-18, z=8), IC6c (x=26, y=54, z=24), IC13 (x=-50, y=-66, z=-8), and IC15 (x=42, y=-70, z=8). These ICs are important in global brand perception. There is large participation of caudal parts of the brain involved in visual and visual associative processing. Table 2 reports in detail the brain regions involved. z-values are color-coded in the range -6.0 (light blue) to -2.6 (dark blue), and 2.6 (red) to 6.0 (yellow). MNI152 standard space. Radiological convention.


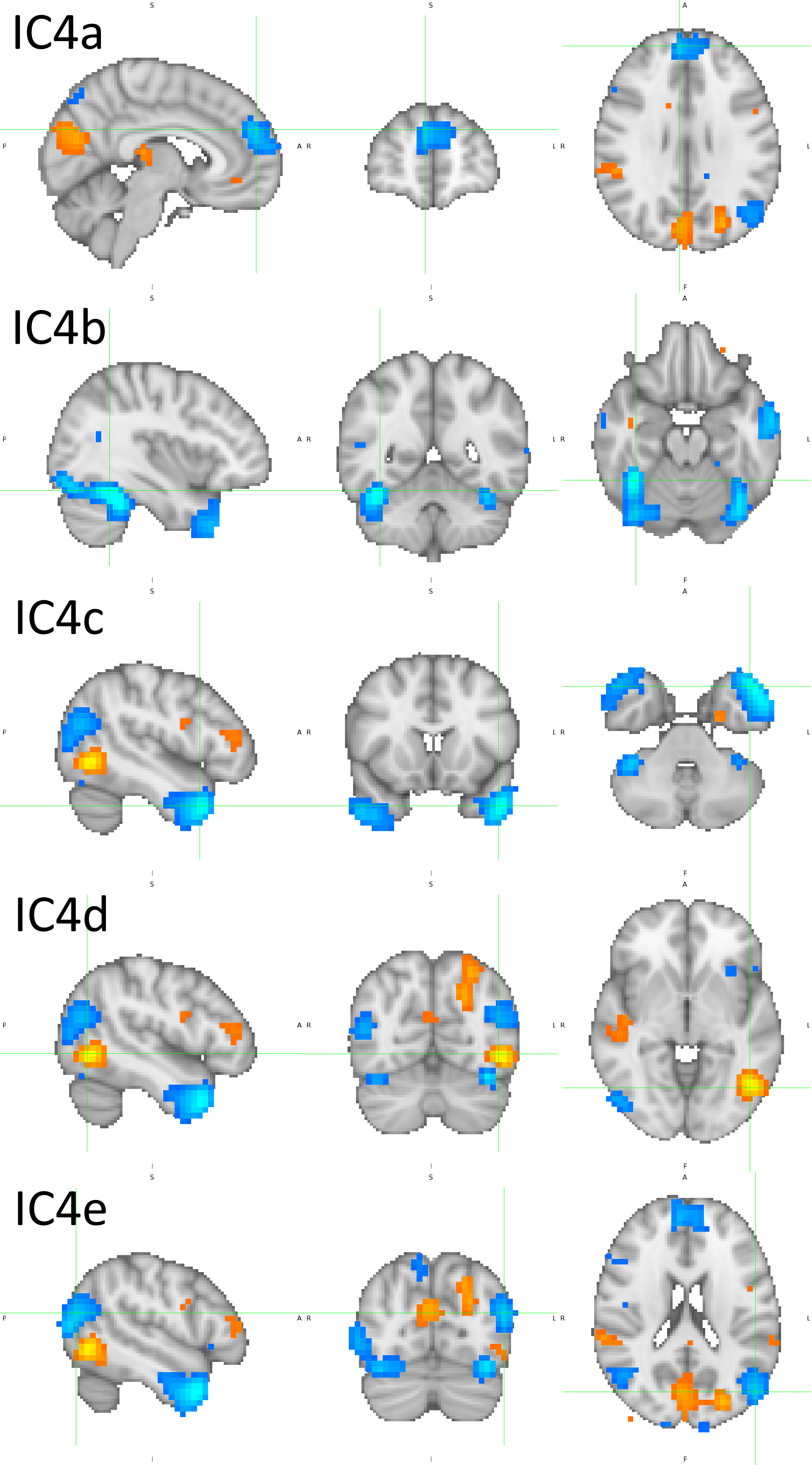


Supplementary Figure 7. Sagittal, coronal, and axial views of IC4a (x=6, y=54, z=28), IC4b (x=38, y=-50, z=-20), IC4c (x=-46, y=14, z=-36), IC4d (x=-46, y=-66, z=-4), and IC4e (x=-50, y=-74, z=20). IC4 is important in BP perception and involves several parts of the brain, caudal, medial, and rostral. Table 3 reports in detail the brain regions involved. z-values are color-coded in the range -6.0 (light blue) to -2.6 (dark blue), and 2.6 (red) to 6.0 (yellow). MNI152 standard space. Radiological convention.


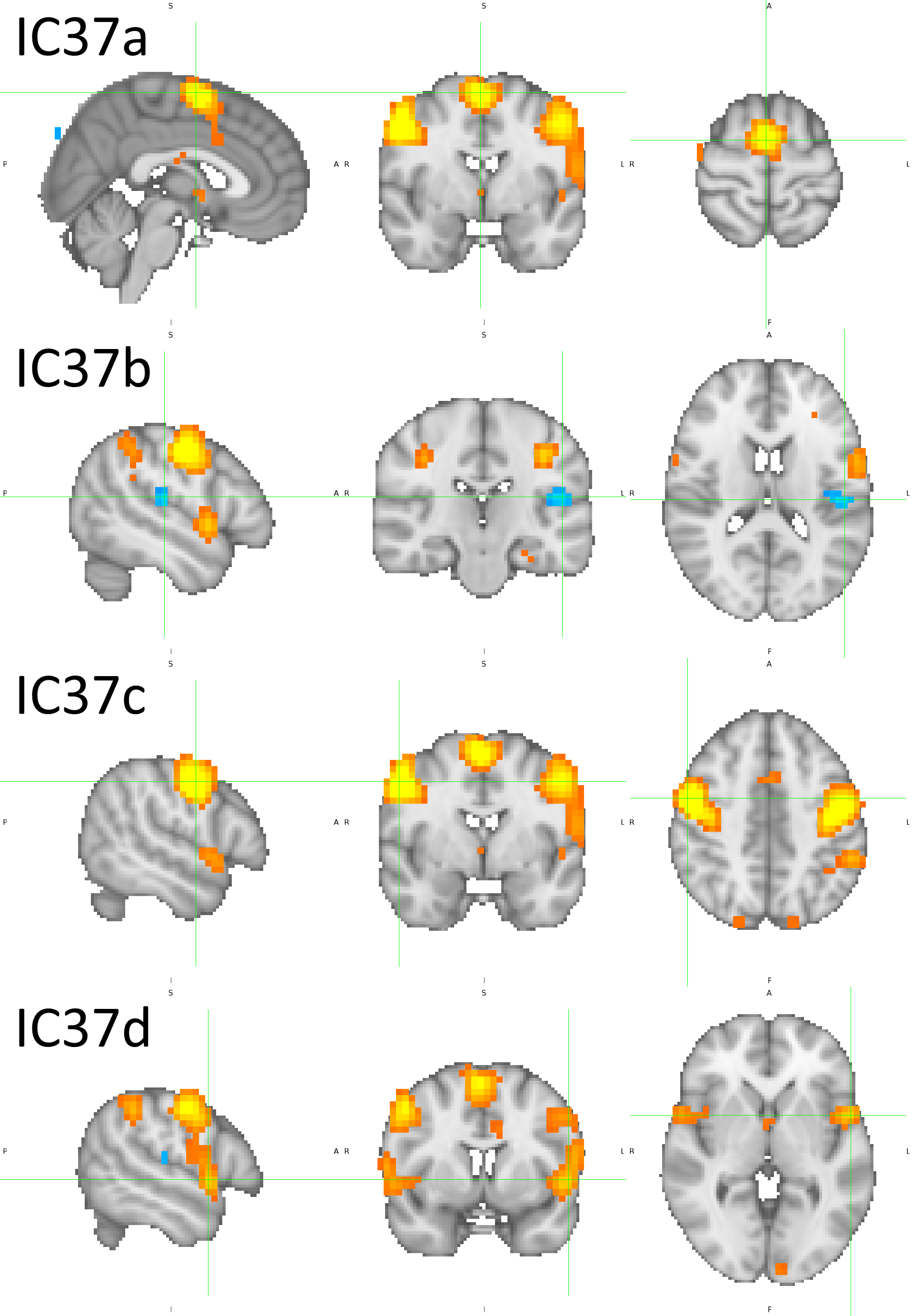


Supplementary Figure 8. Sagittal, coronal, and axial views of IC37a (x=2, y=-2, z=64), IC37b (x=-50, y=-22, z=16), IC37c (x=54, y=-2, z=44), and IC37d (x=-54, y=6, z=0). IC37 is important in BP perception and involves mostly medial parts of the brain. Table 3 reports in detail the brain regions involved. z-values are color-coded in the range -6.0 (light blue) to -2.6 (dark blue), and 2.6 (red) to 6.0 (yellow). MNI152 standard space. Radiological convention
